# Aminooxy Click Modification of a Periodate-Oxidized Immunoglobulin G: A General Approach to Antibody–Drug Conjugates with Dye-Mediated Expeditious Stoichiometry Control

**DOI:** 10.3390/ijms24065134

**Published:** 2023-03-07

**Authors:** Ksenia A. Sapozhnikova, Evgeny L. Gulyak, Vladimir A. Brylev, Vsevolod A. Misyurin, Sergey D. Oreshkov, Anastasiya V. Alexeeva, Dmitry Yu. Ryazantsev, Maria A. Simonova, Ekaterina V. Ryabukhina, Galina P. Popova, Nataliya A. Tikhonova, Natalia A. Lyzhko, Alexander E. Barmashov, Andrey V. Misyurin, Alexey V. Ustinov, Vera A. Alferova, Vladimir A. Korshun

**Affiliations:** 1 Shemyakin-Ovchinnikov Institute of Bioorganic Chemistry, Miklukho-Maklaya 16/10, 117997 Moscow, Russia; 2 Lumiprobe RUS Ltd., Kotsyubinskogo 4, 121351 Moscow, Russia; 3 N.N. Blokhin National Medical Cancer Research Center, Ministry of Health of Russia, Kashirskoye sh. 24, 115478 Moscow, Russia; 4 Department of Chemistry, Lomonosov Moscow State University, Leninskie Gory 1/3, 119991 Moscow, Russia; 5 GeneTechnology LLC, Profsoyuznaya 104, 117485 Moscow, Russia

**Keywords:** ADC, antibody, PRAME, periodate oxidation, oxime ligation, CuAAC, cyanine dyes, cleavable linkers, doxorubicin, MMAE

## Abstract

A universal approach to the construction of antibody–drug conjugates (ADCs) has been developed. It relies on periodate oxidation of naturally present glycans of immunoglobulin G, followed by oxime ligation and, optionally, copper(I)-catalyzed alkyne-azide cycloaddition for conjugation with a toxic payload. The introduction of highly absorbing cyanine dyes into the linker allows for facile determination of the drug–antibody ratio. We applied this methodology to the synthesis of cytotoxic conjugates of an antibody against the tumor-associated antigen PRAME with doxorubicin and monomethyl auristatin E (MMAE). The resultant conjugates retained their affinity to a large extent, yet their cytotoxicity in vitro varied dramatically: while the doxorubicin-based conjugate did not produce any effect on cells, the MMAE-based one demonstrated specific activity against PRAME-expressing cancer cell lines. Importantly, the latter conjugate constitutes the first reported example of a PRAME-targeting ADC.

## 1. Introduction

Antibody–drug conjugates (ADCs) combine the specificity of antibodies with the potency of small molecules. Despite the simplicity of the concept, designing clinically successful ADCs is rather challenging: after 50 years of research and development there are currently about a dozen approved ADC medicines, and over one hundred are in clinical trials [1,2]. The linker connecting the antibody with the toxic payload is pivotal for the stability of the ADC in systemic circulation and key to the efficient and selective release of the payload at the tumor site [3,4,5,6]. The methodology of antibody modification and ADC linker chemistry is a vast subject crucial for ADC development [7,8,9,10].

Our goal was to develop linkers and a methodology suitable for site-specific modification of standard monoclonal IgG antibodies without the need for engineered glycans or amino acids. Acylation of ε-amino side chains of lysine leads to nonspecific modification, occasionally affecting antigen-binding domains [11]. Controlled reduction of cysteine followed by reaction with maleimide or disulfide rebridging reagents is a common technique for ADC assembly [12,13,14,15,16,17,18]. This approach can be considered site-selective because interchain disulfides are more susceptible to reduction than intra-chain ones, and their number is well-known and strictly defined for each IgG subtype. In addition, they are located far from the antigen-binding site. However, this approach requires careful optimization to prevent the loss of integrity of IgG [11,19]. In addition to these classic well-known methods, there is also a whole class of new methods that allow for the introduction of functional groups into IgG with exceptional precision, but which require manipulation of its structure through genetic engineering. Such methods include genetically engineered introduction of unnatural amino acids (UAAs) [20] with biorthogonal functional groups [21], as well as the introduction of various peptide tags for subsequent selective enzymatic ligation [4,22,23,24]. These methods are complicated and do not allow using an “off the shelf” antibody.

Other approaches are based on the modification of immunoglobulin glycans. Mammalian IgG-class antibodies are glycosylated in the Fc region of each heavy chain. The glycans have an obligatory mannose core and are attached to the side chain of the amino acid residue asparagine-297 via two N-acetyl glucosamine residues. The glycans can also be decorated with fucose, galactose, and sialic acid residues [25,26].

Several enzymatic approaches have now been developed for conjugation, such as ligation with microbial transglutaminase (MTGase) [4] or various types of glycan engineering, including remodeling with mutant glycotransferases with unnaturally functionalized oligosaccharides [27]. However, all these approaches, despite their high site-specificity, are not very common and require special mutant enzymes.

In this light, simple chemical modification of glycans attached to Fc domains appears to be a favorable alternative for site-specific modification of monoclonal IgGs. In particular, periodate oxidation of Fc glycans generates aldehyde groups without affecting the antigen-binding properties of the antibody [28]. Modification of periodate-oxidized glycans has only once been used for ADC assembly, and no information on the cytotoxicity of the resultant conjugate was reported [29]. We aimed to further study the scope of applicability of this approach.

Oxime ligation of carbonyl compounds with oxyamines is a convenient synthetic pathway to stable bioconjugates [30]. Oxyamines form a strong, almost irreversible bond to the carbonyl group, stronger than that of hydrazides, hydrazines, or amines, and do so very quickly [30]. However, the high reactivity of oxyamines even in salt form reduces the panel of available reagents. Recently, we found that the 1-ethoxyethylidene-protecting group on oxyamines allows for performing oxime ligation on periodate-oxidized IgG in situ in an acidic acetate buffer with pH 3.6, with such protected oxyamine derivatives of fluorescent dyes having a virtually unlimited shelf lifetime [31]. These findings prompted us to apply protected dye oxyamines to the ADC technology. The elements of the simplest conjugate for a proof-of-concept study are as follows: a periodate-oxidized antibody, a 1-ethoxyethylidene-protected oxyamine linker containing a dye to control the stoichiometry, a fragment responsible for enzymatic cleavage in the endosome, and a cytotoxic drug.

For the present study, we choose a PRAME-specific monoclonal IgG1 [32,33] as the tumor-targeting antibody. Preferentially expressed antigen of melanoma (PRAME) is a small human protein which is seldom expressed in the cytosol of normal cells (it is found in small amounts in the cytosol of cells of the male reproductive system) but is present in the membrane of melanoma and acute leukemia tumor cells. PRAME can participate in the inhibition of the retinoic acid signaling pathway and retinoic acid-mediated differentiation and apoptosis. Overexpression of the membrane form of PRAME in tumors and its low levels in the cytosol of normal somatic tissues make it a promising target for cancer therapy. In our previous works, we showed the possibility of using fluorescent antibodies to the PRAME protein obtained by oxime ligation as a molecular diagnostic tool [31,34,35]. We demonstrated that fluorescent anti-PRAME antibodies allow for immunotyping bone marrow cells of patients with acute leukemia for the presence of the PRAME protein [31], which significantly affects the course of the disease and further prognosis [36,37,38,39,40,41,42].

We chose doxorubicin, a well-known anticancer drug, as the model payload. Doxorubicin intercalates into DNA and inhibits topoisomerase II, preventing transcription and replication [43,44]. In addition, it can generate free radicals and thus do damage to nearby DNA. Some papers have also reported that this drug can deregulate histone function in active chromatin [45]. Thus, doxorubicin induces apoptosis in actively dividing cells, such as tumor cells. The drug works at micromolar concentrations and can be used on its own without being part of an ADC, which typically requires a much more toxic payload (MMAE and MMAF, PBD-dimers, etc.) [46]. However, due to its combined mechanism of action, multiple potential modification sites, and hydrophilicity, it is a suitable drug for our proof-of-concept study. We also set out to try another drug with a different mechanism of action and higher cytotoxicity, such as monomethyl auristatin E (MMAE), for comparison [47]. This is an antimitotic drug which interrupts cell division by inhibition of tubulin assembly. This drug is well-known in the ADC field: many FDA-approved antibody–drug conjugates contain MMAE as the cytotoxic part [48].

**Figure 1:**
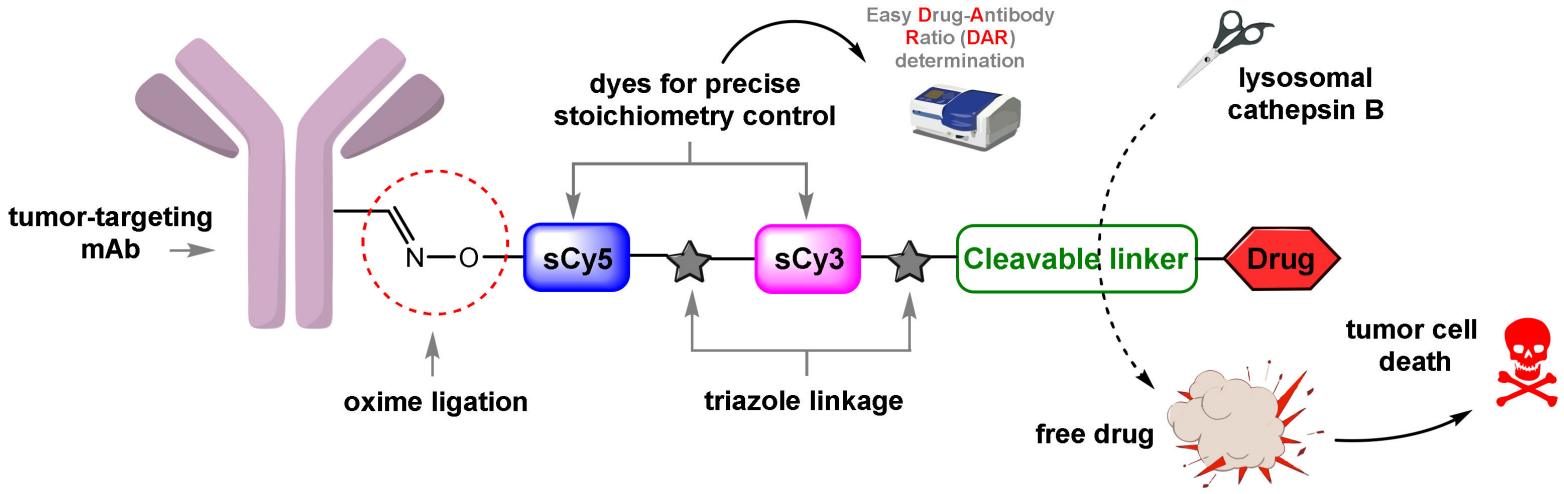
The methodology affords a site-specific ADC with facile stoichiometry control.

For the cleavable part, we chose the Val-Cit-PABC cathepsin B-cleavable linker. We decorated this structure in different ways with protected tetra ethylene glycol-based oxyamine derivatives and highly absorbing hydrophilic sulfo-cyanine dyes (sCy3 and sCy5). Cyanine dyes have two functional sites and are therefore well-suited for insertion into the linker. Herein, we report the synthesis of oxime-linked anti-PRAME ADCs that have cyanine dyes into expeditious their structure introduced for monitoring of the stoichiometry (Figure 1).

## 2. Results and Discussion

### 2.1. Approaches to the Assembly of ADCs

**Scheme 1:**
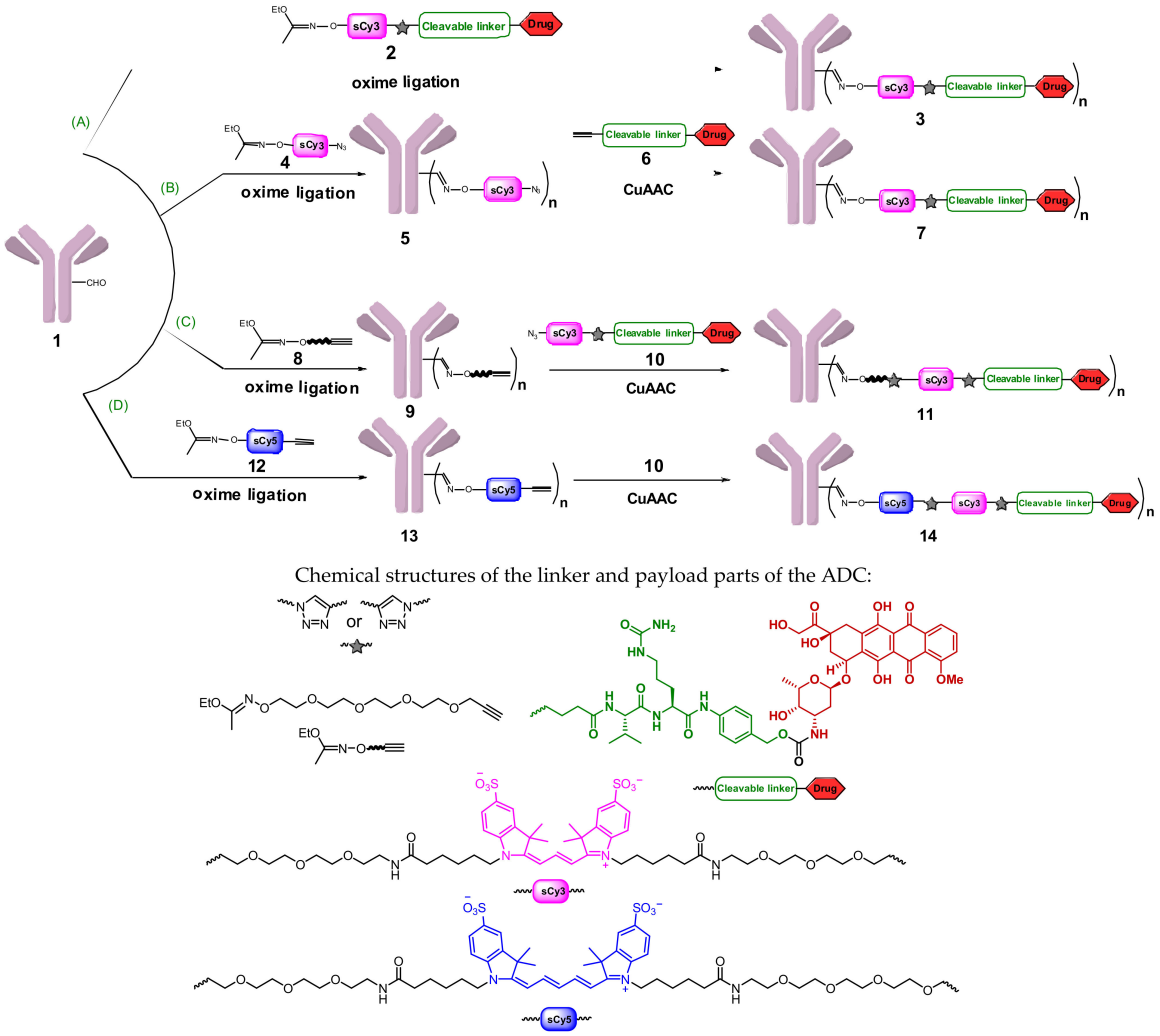
Four approaches (**A**–**D**) to the assembly of doxorubicin-based ADCs from periodate-oxidized IgG via oxime ligation with cyanine dyes in the linker.

We tested four different strategies for ADC assembly (Scheme 1). Approach A involves a reaction of the oxidized antibody **1** with conjugate **2** comprised of the linker dye oxyamine and Val-Cit-PABC-doxorubicin. Unfortunately, this approach proved incompatible with drug loads containing a carbonyl group, such as doxorubicin. To overcome this limitation, we developed approach B consisting of oxime ligation between oxyamine-azide dye-containing linker **4** and oxidized antibody **1** followed by a copper-catalyzed click reaction with reagent **6**. However, reagent **6** was not soluble enough in aqueous buffers, which made it unsuitable for conjugation. In approach C, payload solubility was markedly improved by the incorporation of a hydrophilic dye into azide linker **10**. The oxidized antibody **1** was modified with alkyne linker **8**, which was followed by CuAAC with **10**. In approach D, for better stoichiometry control at the first step, we decided to introduce an additional fluorescent dye (sCy5) into alkyne linker **12**. Oxime ligation with oxidized antibody **1** and subsequent CuAAC with **10** yielded the desired conjugate. Thus, we were able to control the stoichiometry of the assembly of the ADC with oxime ligation and CuAAC at every step. Finally, we used approach A to synthesize a more toxic 6H8-MMAE conjugate, **32**.

#### 2.1.1. Approach A

**Scheme 2:**
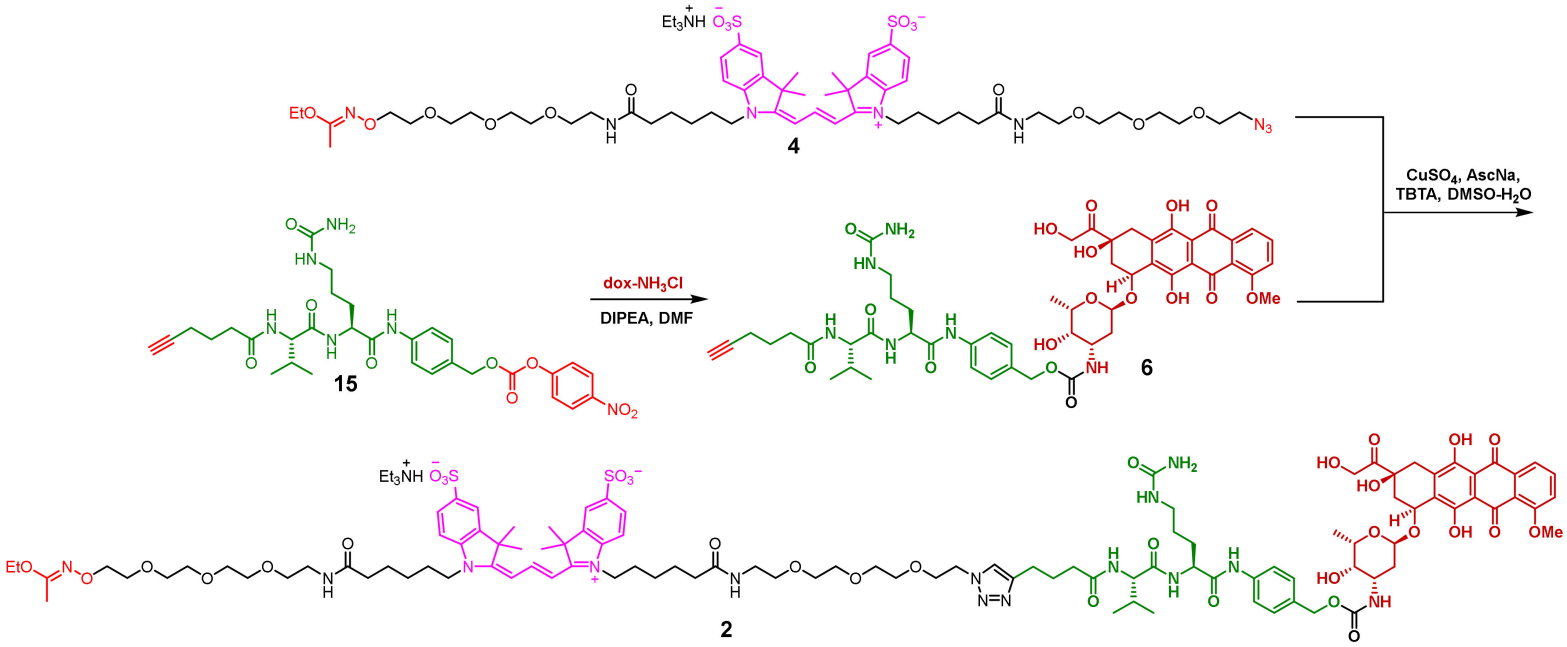
Synthetic scheme for approach A.

**Scheme 3:**
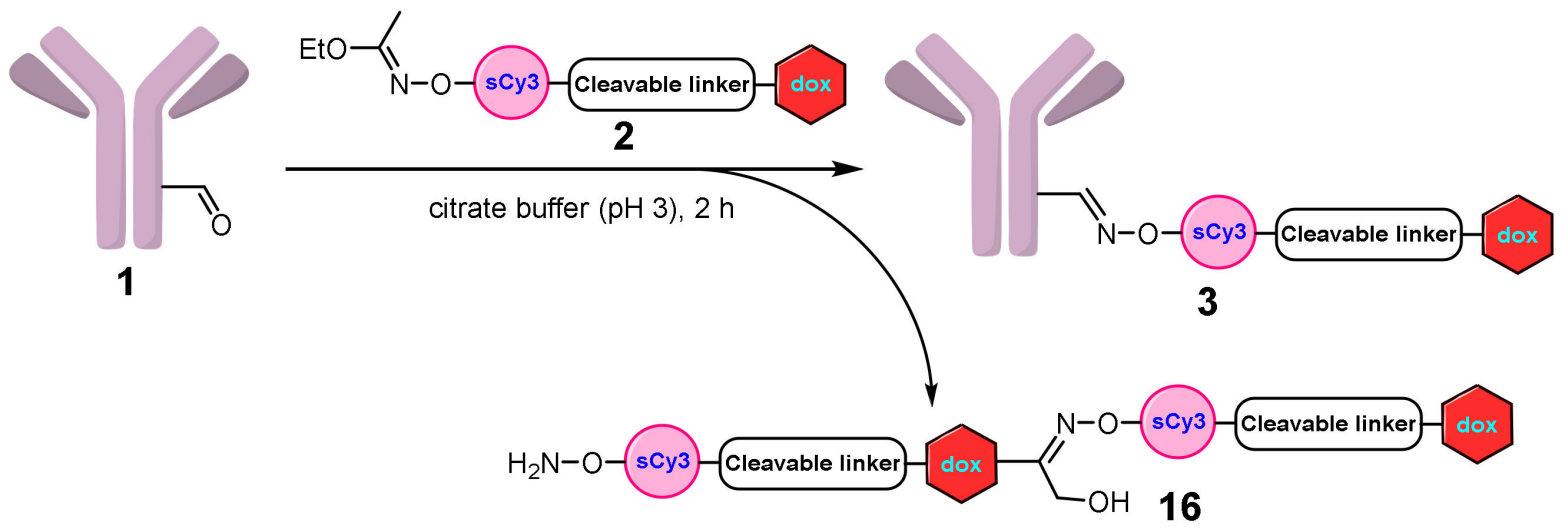
Side reaction in the course of ADC synthesis via approach A.

The most obvious idea was to synthesize the entire molecule containing a functional group for conjugation with the antibody (ethoxyethylidene-protected oxyamine), a stoichiometry-controlling dye (sCy3), and a drug (doxorubicin or MMAE) with a cleavable linker (Val-Cit-PABC) (Scheme 2). Such reagent **2** was supposed to react with oxidized antibody **1**, immediately giving ADC **3** (Scheme 1A). ADC stoichiometry should be easily controllable by comparing IgG absorbance at 280 nm (ε 210,000 M^−1^cm^−1^) and sCy3 absorbance at 548 nm (ε 162,000 M^−1^cm^−1^). Indeed, in an acidic buffer, the ethoxyethylidene protecting group of **2** was removed in situ and the free hydroxylamine reacted with the carbonyl group of the antibody to form the target conjugate. However, certain difficulties arose. Our hope was that the aldehyde groups of oxidized IgG **1** would be much more reactive than the ketone of doxorubicin. However, in practice it turned out that the difference in reactivity is insufficient. Upon incubation in an acidic buffer, oligomeric products were formed by oxime ligation of the linker molecule to the carbonyl group of doxorubicin belonging to a different linker molecule (Scheme 3). The oligomers were separated by gel filtration, but their formation reduced the efficiency of oxime ligation to antibodies and resulted in inability to obtain a DAR (drug-antibody ratio; the value showing how many payload molecules there are per antibody molecule) higher than 0.29. Therefore, this approach turned out to be imperfect for doxorubicin, although it has value for payloads that do not contain a reactive carbonyl group, such as MMAE (described in more detail in Section 2.1.5).

#### 2.1.2. Approach B

To eliminate the main drawback of approach A, we “split” the linker molecule **2** into two parts—the “bifunctional dye” and the “linker-payload”. First, we introduced azido-containing dye **4** [35] (Scheme 2) into the antibody molecule using oxime ligation as above. This step worked well and allowed us to accurately estimate the number of azido groups and was followed by “blind” labeling in CuAAC click conditions with weakly absorbing reagent **6**. In a CuAAC reaction [13,14], we can use a payload with a carbonyl group without any problem. However, since doxorubicin absorbs weakly (ε_480_ 11,500 M^−1^cm^−1^), the yield of the CuAAC reaction cannot be measured spectrophotometrically. Thus, direct evaluation of the DAR is impossible.

**Scheme 4:**
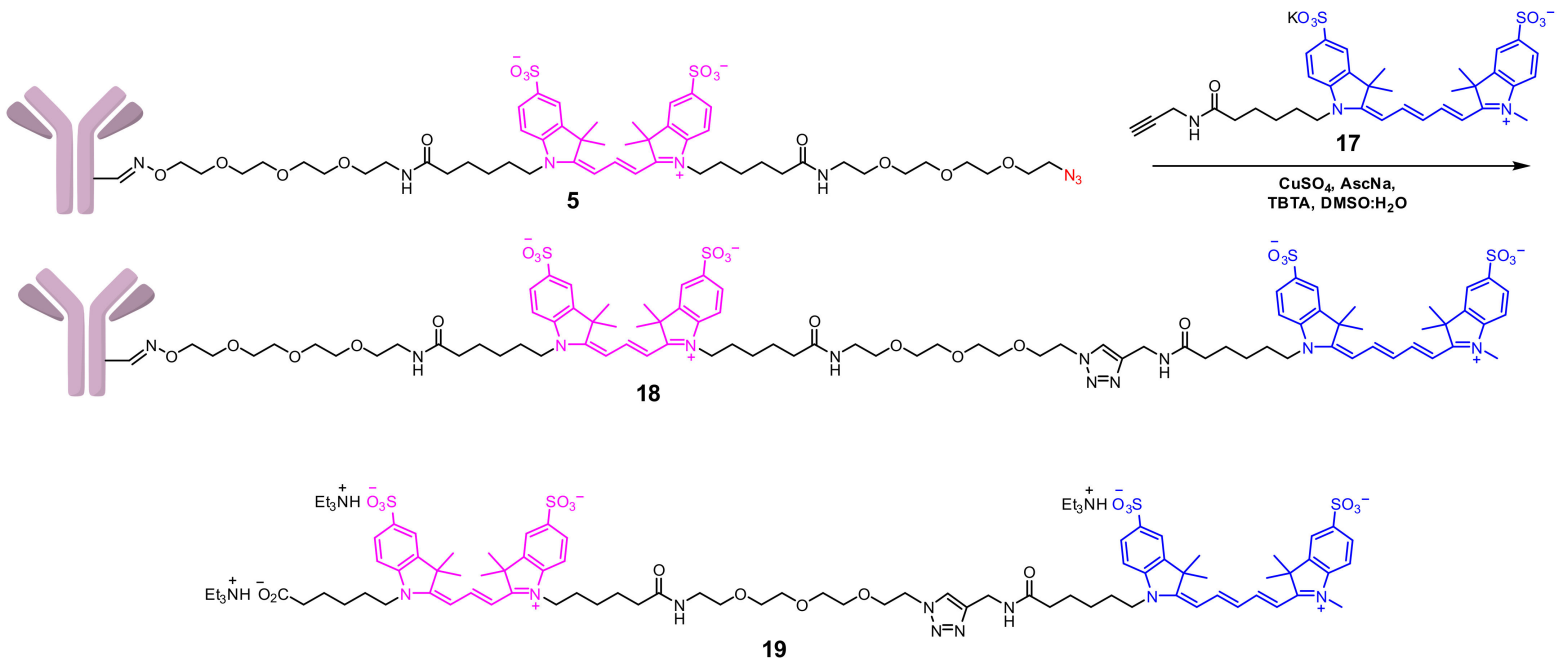
Model click reaction for drug–antibody ratio determination via approach B.

**Figure 2:**
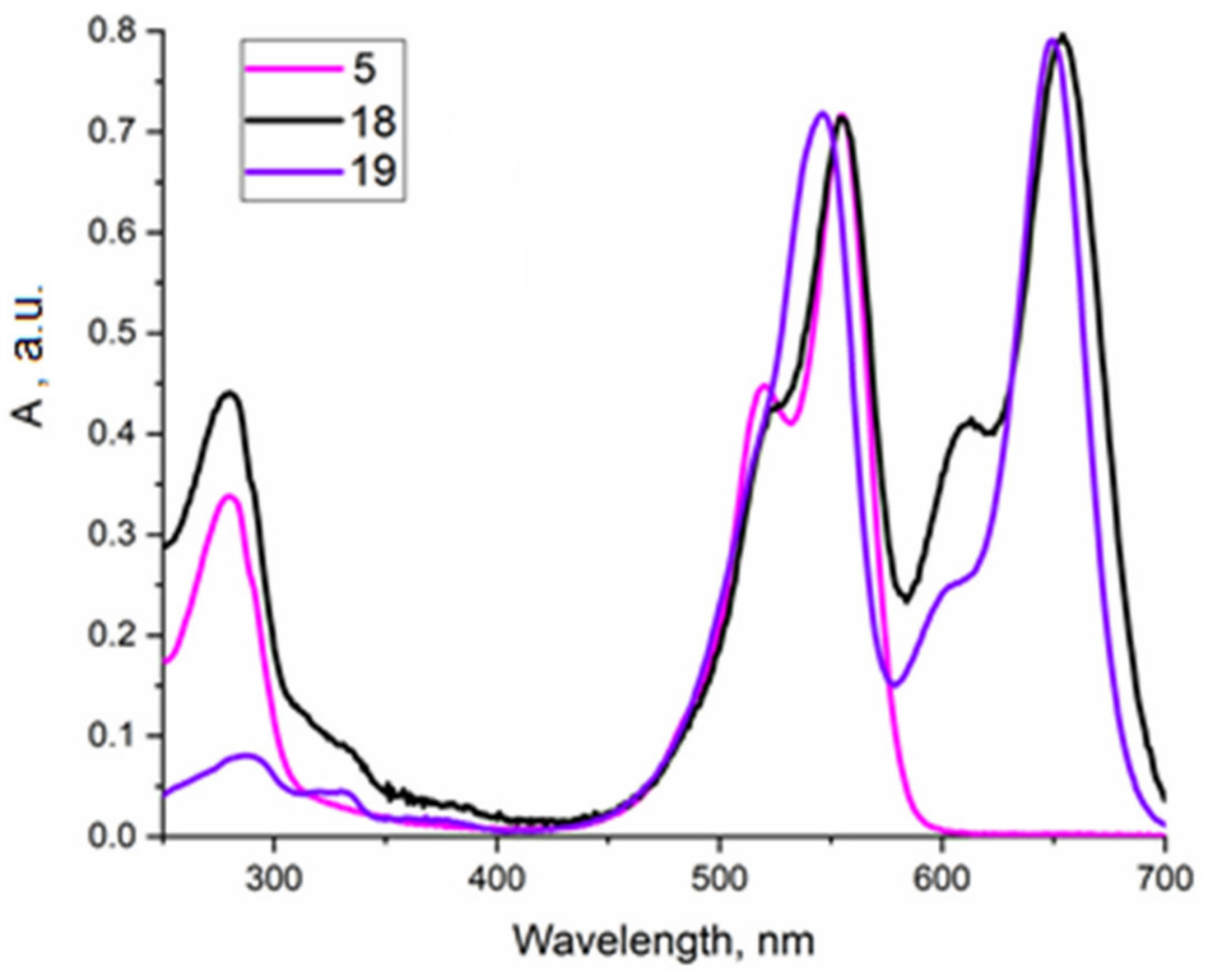
UV/Vis absorbance spectra of **5** and **18** compared to sCy3-sCy5 conjugate **19** in aqueous solution.

As we were not confident that the copper-click reaction in presence of such a complex and sensitive biomolecule [49] as an antibody was quantitative, we studied this question in some detail. We selected the mildest and most effective conditions for the click reaction: the optimal catalyst, ligand, their concentration, and time of reaction under which no aggregation of the antibody and no significant decrease in affinity were observed. We then performed a CuAAC reaction on conjugate **5** with sCy5 alkyne **17** and obtained a probable yield of the reaction (Scheme 4) by measuring the ratio of maximum absorbance of sCy3 to that of sCy5 (Figure 2). The low-molecular weight sCy3-sCy5 conjugate **19** [12] (Scheme 4) was used as a reference for 100% yield of the click reaction. Antibody conjugate **18** had almost the same sCy3/sCy5 absorbance ratio as the one observed for **19**, which indicated that CuAAC proceeded quantitatively (Figure 2).

However, yet another problem was the low solubility of reagent **6** in water. This made it difficult to work with under conditions suitable for biomolecules. To dissolve compound **6**, we had to add a large amount of DMSO (up to 30%), causing IgG denaturation. Although a useful and stoichiometry-controlled procedure of derivatization of carbonyl group-containing proteins with an aliphatic azide was developed within approach B, poor solubility of reagent **6** makes this approach useless for ADC synthesis. Another disadvantage, as mentioned earlier, is the lack of precise control at the CuAAC step.

#### 2.1.3. Approach C

**Scheme 5:**
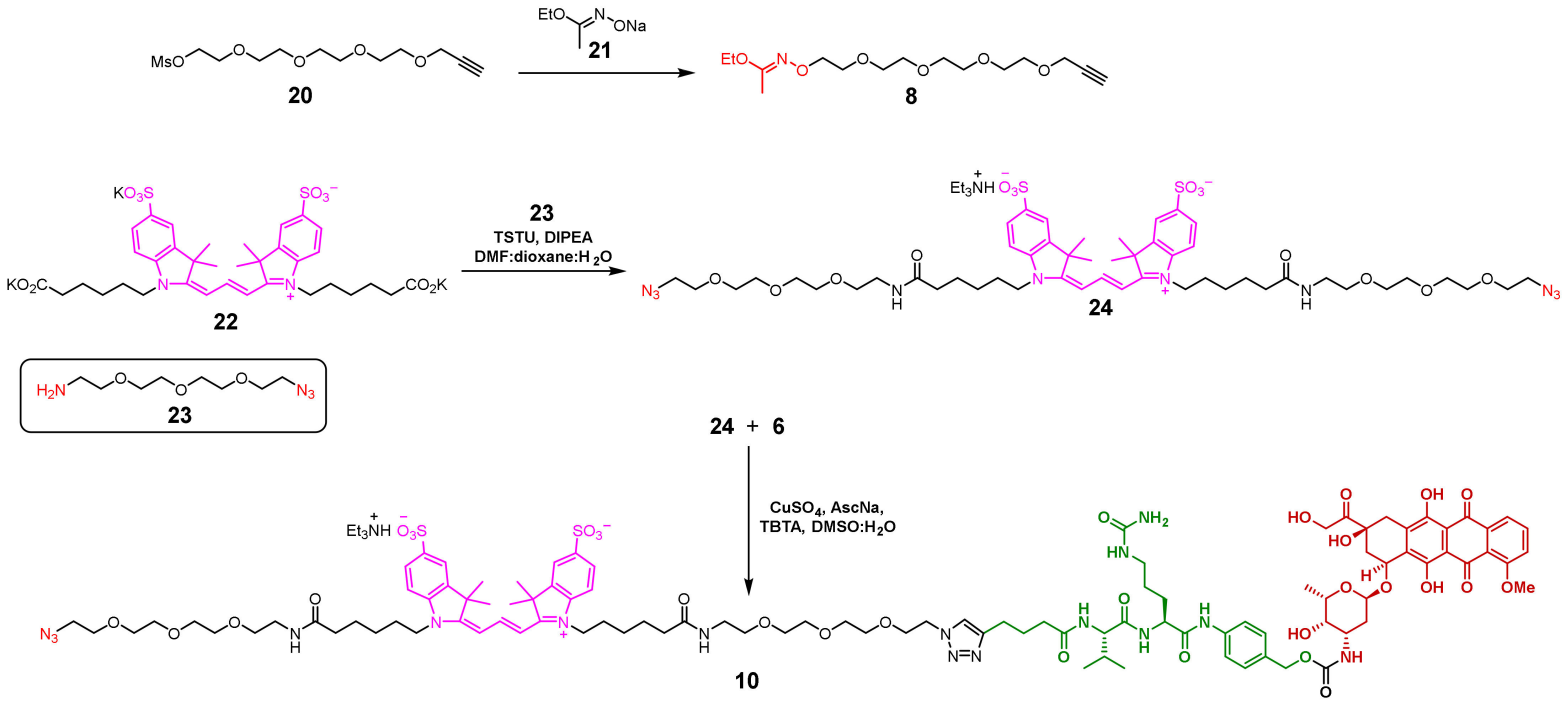
Synthesis of reagents for approach C.

To increase the solubility of the modifying reagent and achieve precise DAR control, we synthesized reagent **10**, which indeed turned out to be much more soluble than reagent **6** due to the hydrophilic nature of the sCy3 dye (Scheme 5). Oxidized antibodies were “blindly” modified with alkyne **8** (Scheme 5) in conditions as above (Scheme 1). At this stage, it was not known how many alkyne groups were introduced, but our hope was that the degree of labeling could be inferred from the results of antibody conjugation with sCy3 within approach B, given that the same conditions were used. Next, alkyne-containing “blind” antibody **9** was modified using reagent **10** by CuAAC (Scheme 1). The resulting conjugate could be directly spectrophotometrically examined to determine the DAR. The observed values of the drug–antibody ratio were close to 1. This was unusual, since in approach B, under similar conditions, oxime ligation yielded conjugate **5** with a degree of labeling of more than three. While developing approach D (described in Section 2.1.4), we noticed that, as a rule, after CuAAC, the absorbance of the antibody conjugates at 280 nm markedly increased (roughly twofold), and this increase could not be attributed to the addition of the dye and payload to the conjugate. While the reasons for that remained unclear, the inflation of the absorbance at 280 nm precluded us from accurately determining the DAR in approach C, as the measured value would inevitably be lower than the real one, and necessitated the use of another highly absorbing dye to determine the degree of modification of the antibody with alkyne groups, which is described in Section 2.1.4. (Approach D).

#### 2.1.4. Approach D

**Scheme 6:**
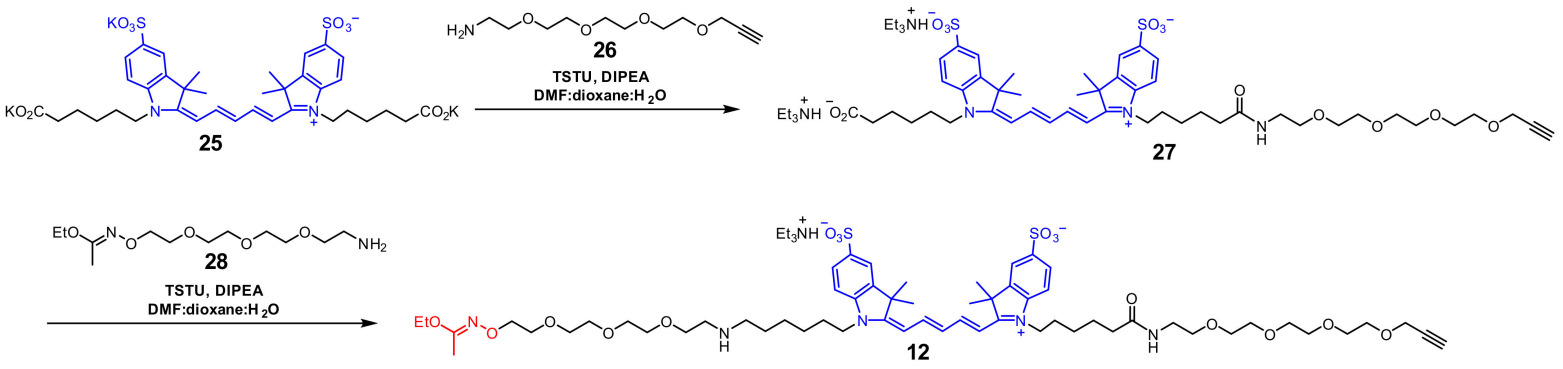
Synthesis of reagents for approach D.

**Figure 3:**
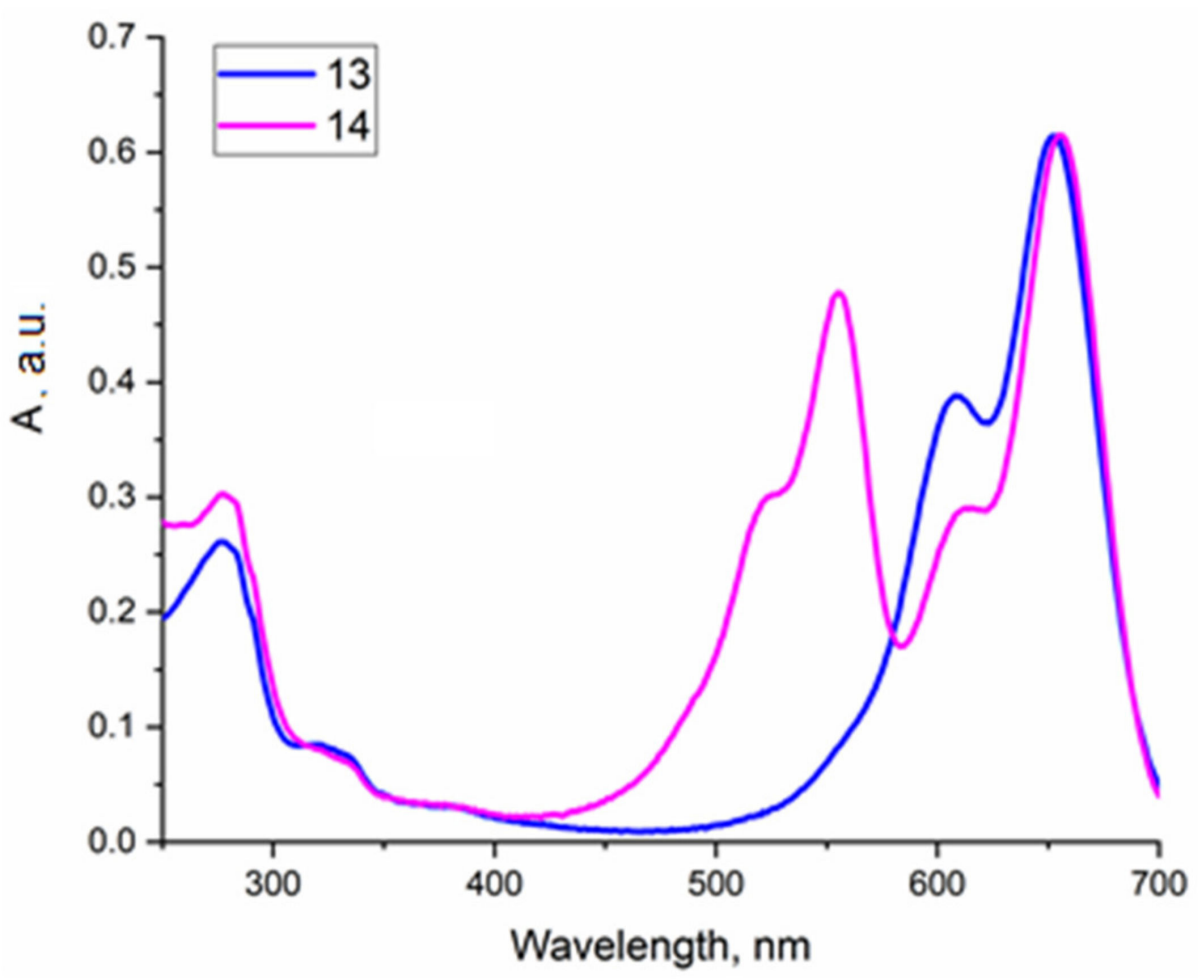
UV/Vis absorbance spectra of intermediate **13** and ADC **14** in PBS.

**Figure 4:**
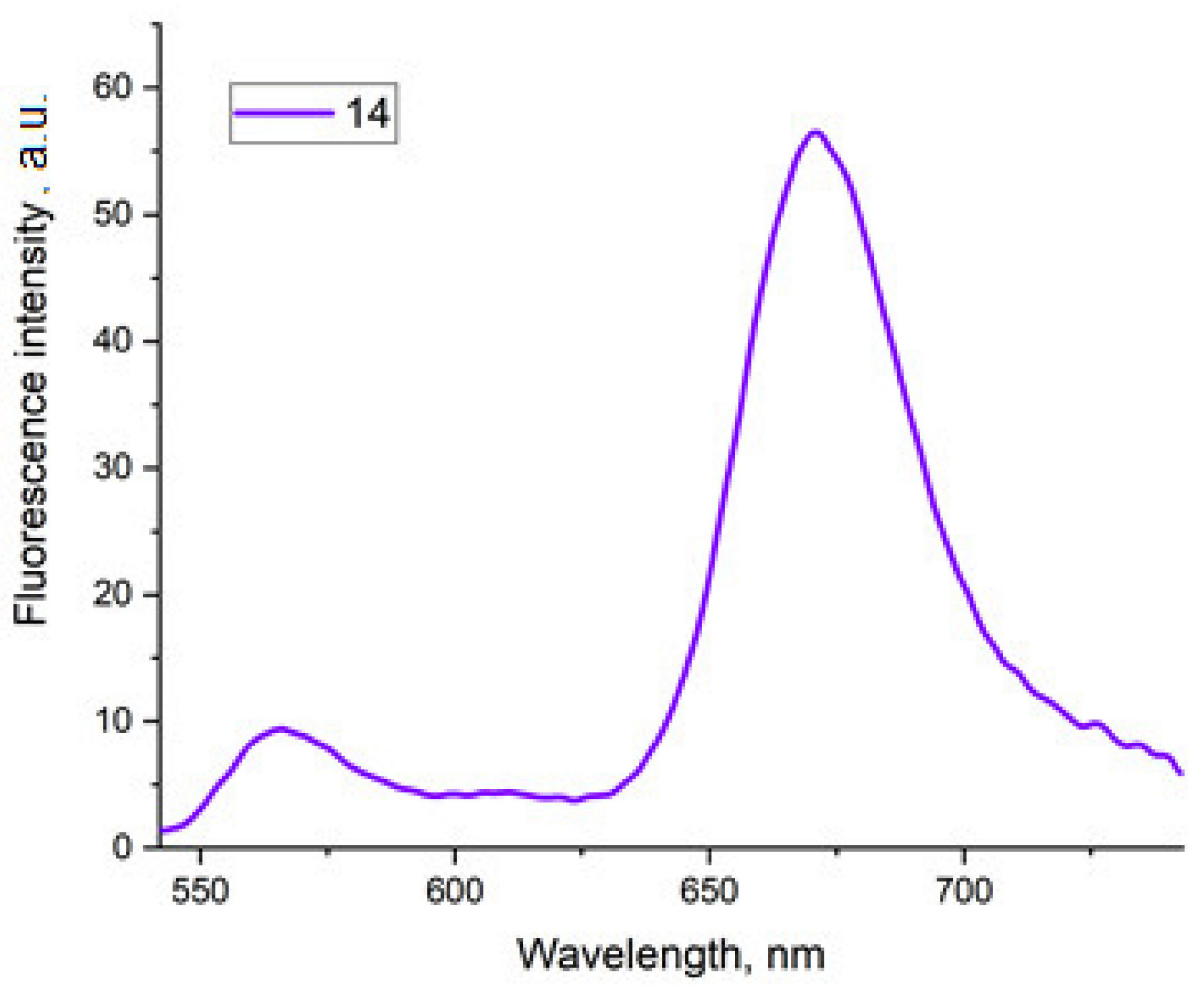
Fluorescence spectrum of ADC **14** (emission spectrum, excitation wavelength: 520 nm).

Although approach C made it possible to obtain a conjugate with all the desired parameters, it was not possible to accurately estimate the DAR due to the unfortunate effect of CuAAC on the absorbance at 280 nm. Therefore, the procedure was changed to use sCy5 bifunctional reagent **12** instead of alkyne-oxyamine **8** (Scheme 1 and Scheme 6). The dye absorbance allows the control of the stoichiometry of the alkyne modification of the antibody. Thus, we obtained the ability to carry out the process of assembling the conjugate with precise stoichiometry control at each stage. Upon modification of antibody **1**, conjugate **13** was obtained with a degree of labeling of 2.5. Subsequent click reaction with reagent **10** yielded conjugate **14** with a sCy3/sCy5 absorbance ratio at their λ_max_ corresponding to 84% yield of the click modification, the DAR thus being 2.1 (Figure 3). To additionally confirm the structure of conjugate **14**, we recorded its fluorescence emission spectrum (Figure 4). We observed the FRET effect for the sCy3-sCy5 pair, which indicates their proximity.

In summary, the advantages of approach D are excellent stoichiometry control on each step of modification of the oxime-linked ADC and the possibility of measuring the DAR spectrophotometrically in case the molar absorbance value at 280 nm is compromised.

#### 2.1.5. Synthesis of the 6H8-MMAE Conjugate

**Scheme 7:**
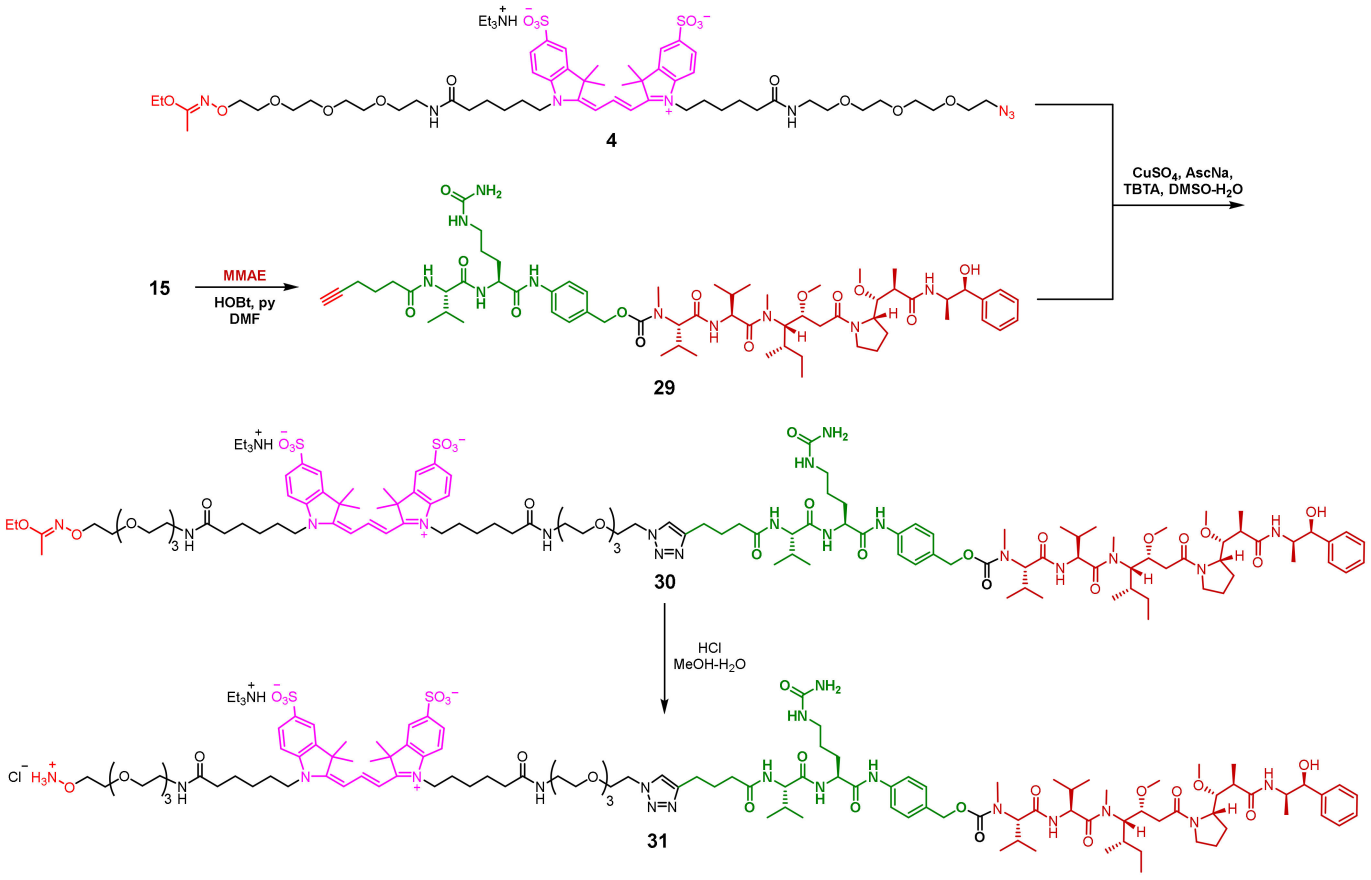
Synthesis of reagent **31**.

Seeking to increase the cytotoxicity of the anti-PRAME ADCs, we turned our attention to monomethyl auristatin E. Unlike doxorubicin, MMAE does not have reactive carbonyl groups and is thus compatible with oxime ligation. This renders it suitable for use in conjugation approach A. To this end, we synthesized the previously reported alkyne-bearing linker-drug **29** and coupled it with sCy3-based protected oxyamine **4** to obtain conjugate **30** (Scheme 7).

**Figure 5:**
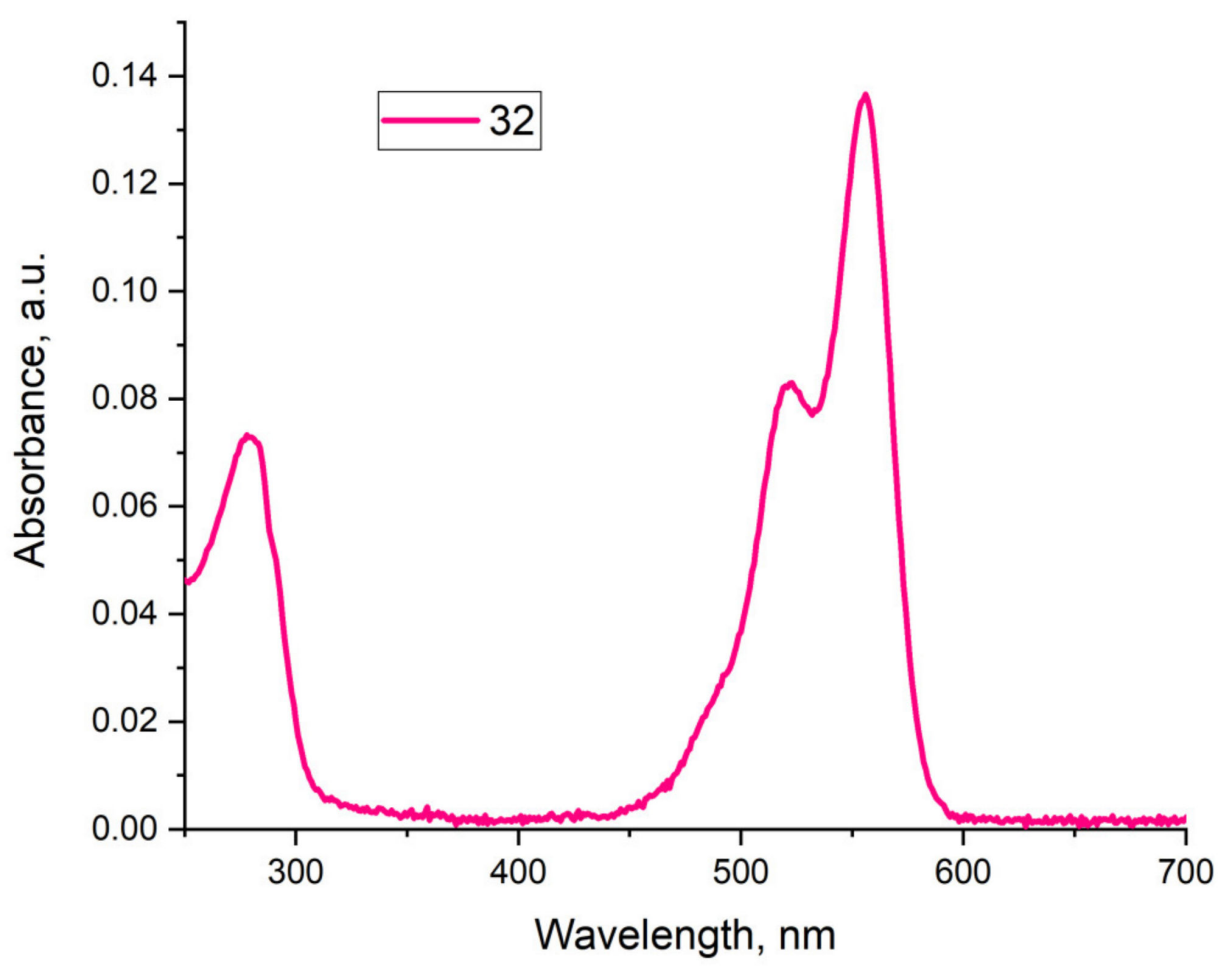
UV/Vis absorbance spectrum of conjugate **32**.

It should be noted that oxyamine deprotection in acidic aqueous buffers is considerably slower, which means that a large excess of protected oxyamine payload needs to be used in the ligation reaction with the oxidized antibody to produce a conjugate with a sufficient DAR. In order to reduce the number of equivalents of the payload required for conjugation and facilitate the subsequent purification, we removed the 1-ethoxyethylidene group in methanolic HCl. The reaction was complete within a few minutes, yielding deprotected linker-drug **31**. Upon its incubation with the oxidized 6H8 antibody, a conjugate with a DAR of 3.0 was obtained (Figure 5).

### 2.2. Affinity Determination

**Figure 6:**
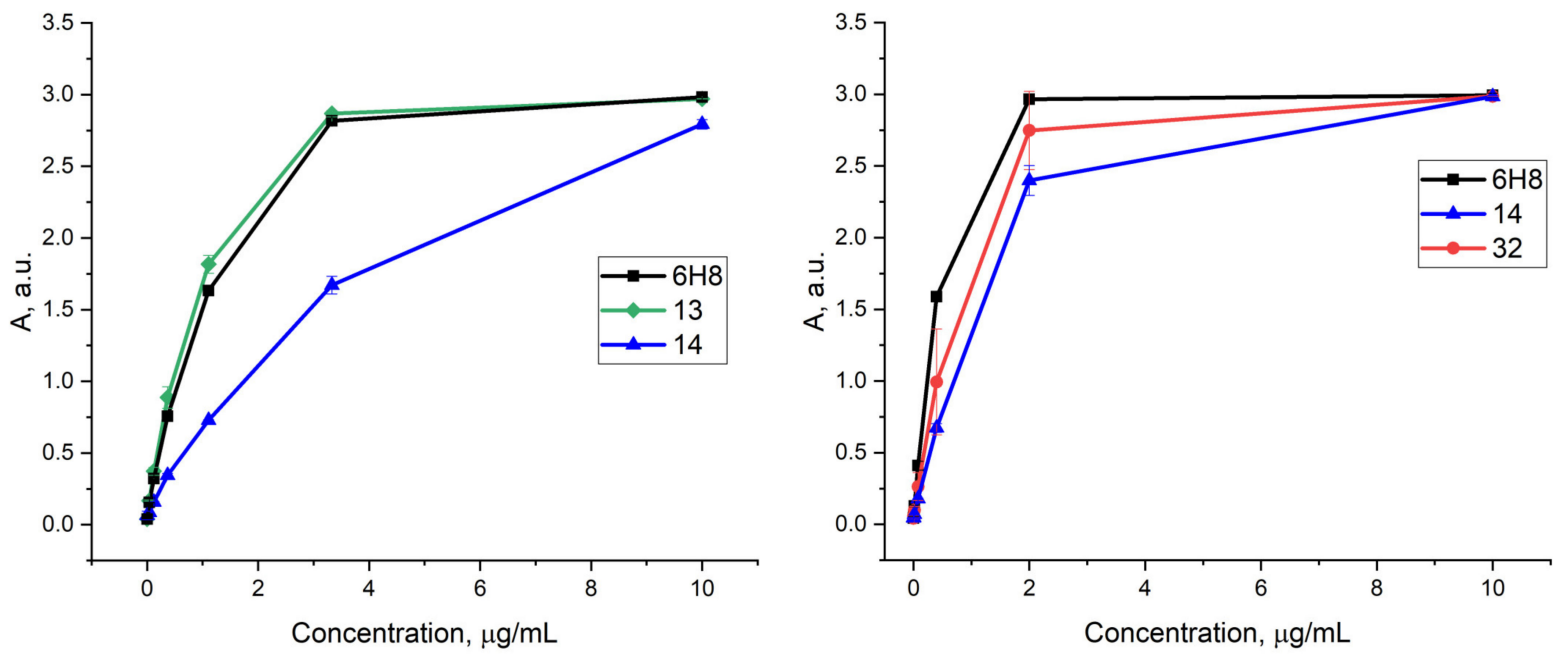
Affinity of conjugates **13**, **14**, and **32** analyzed by ELISA.

We performed an ELISA assay using immobilized recombinant PRAME protein to determine the impact of modification on the affinity of 6H8. It was found that oxime ligation-yielding conjugates **13** and **32** did not compromise antigen binding, while the subsequent CuAAC used to assemble ADC **14** led to a modest decrease in affinity (Figure 6).

### 2.3. Cell Viability Assay

**Figure 7:**
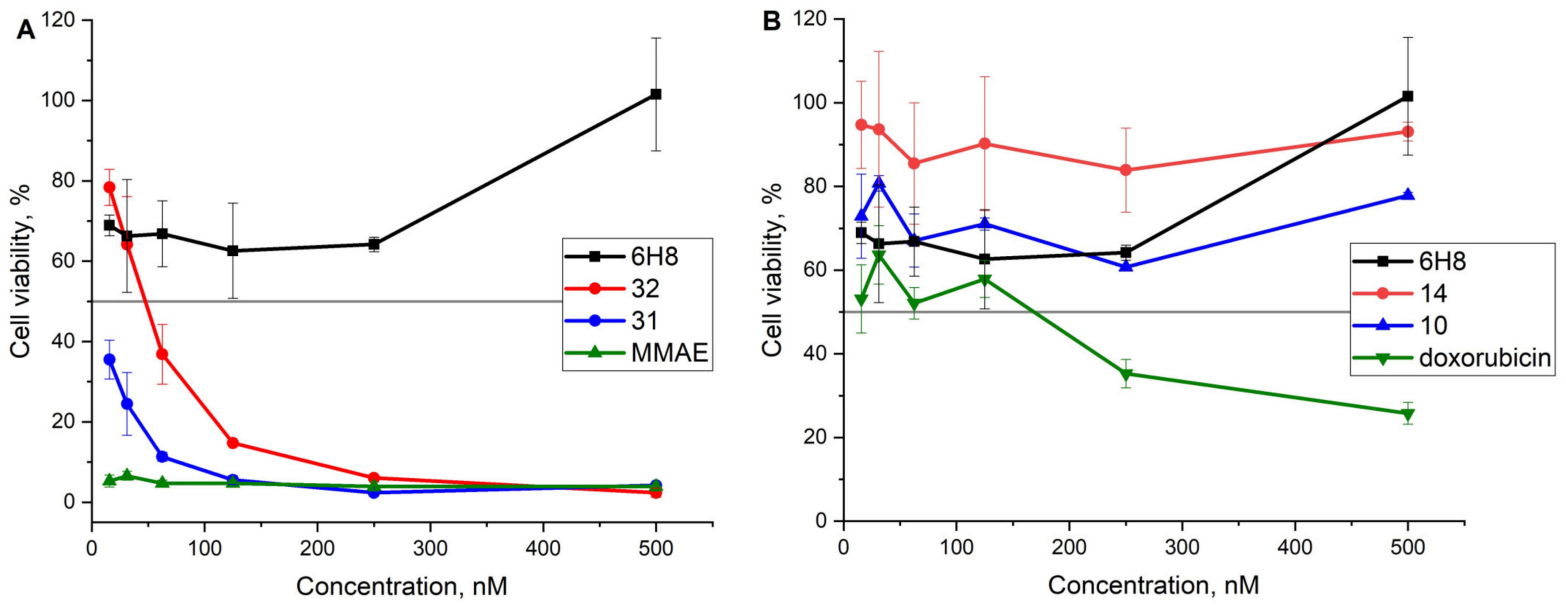
Viability of PRAME (+) human melanoma cell line MelP analyzed by MTT assay following 72 h incubation with **31**, **32**, MMAE, and 6H8 (**A**); **14**, **10**, doxorubicin, and 6H8 (**B**).

**Figure 8:**
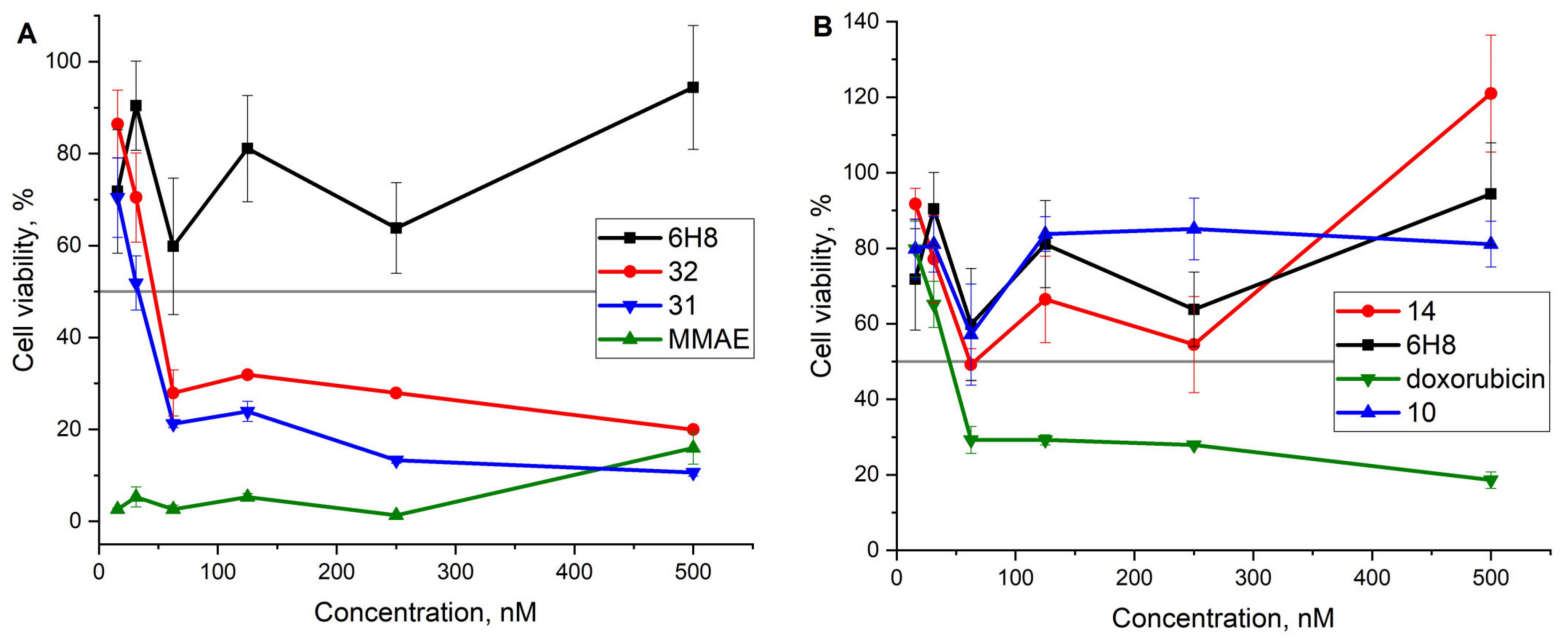
Viability of PRAME (+) human acute monocytic leukemia cell line THP-1 analyzed by MTT assay following 72 h incubation with **31**, **32**, MMAE, and 6H8 mAb (**A**); **14**, **10**, doxorubicin, and 6H8 (**B**).

**Figure 9:**
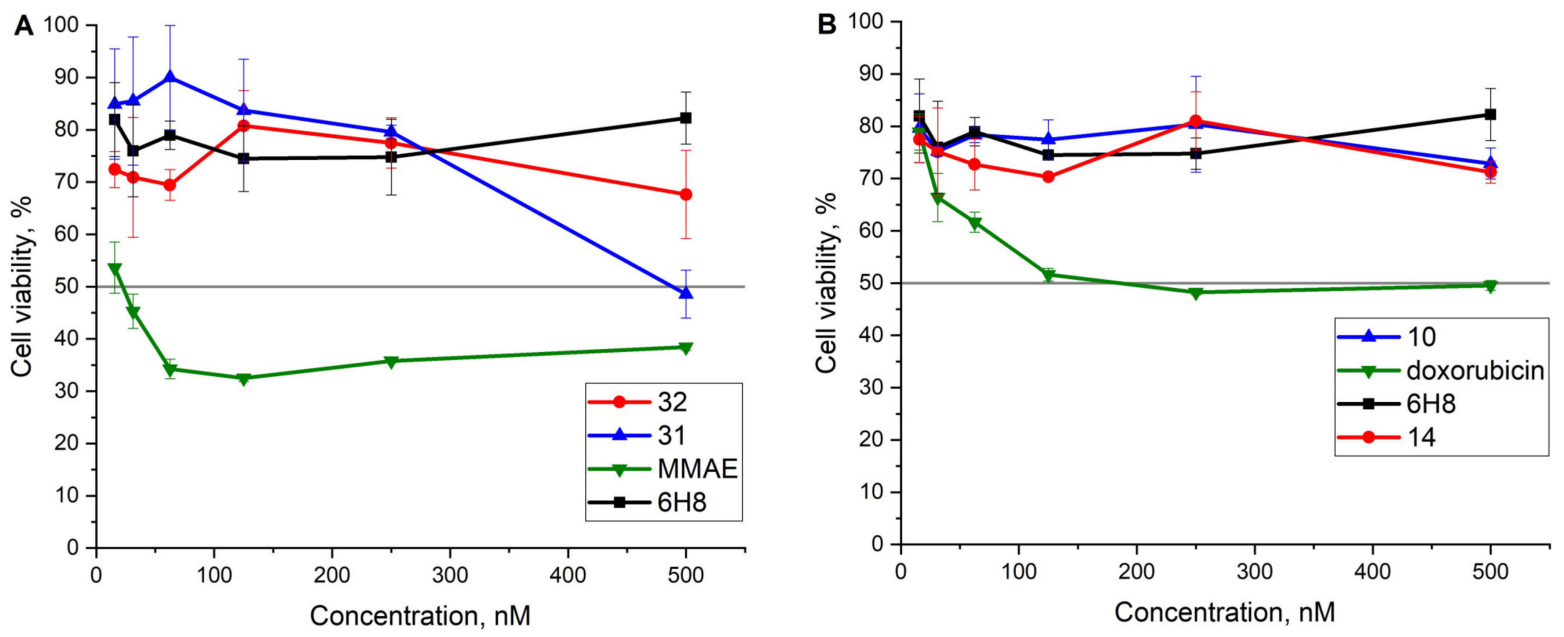
Viability of PRAME (-) murine colorectal carcinoma cell line CT26 analyzed by MTT assay following 72 h incubation with **31**, **32**, MMAE, and 6H8 mAb (**A**); **14**, **10**, doxorubicin, and 6H8 (**B**).

The cytotoxic effect of ADCs **14** and **32** was tested on PRAME-expressing cancer cell lines MelP [50] and THP-1 [51] and the PRAME-negative cancel cell line CT26 [52] in an MTT assay (Figure 7, Figure 8 and Figure 9). We chose only those positive cell lines that are known to express the membrane form of the protein [33,51]; in the PRAME-negative cell line, the antigen, including its cytosol form, is absent [52]. For the cytotoxicity study, ADC **14** with a DAR of 1.8 and ADC **32** with a DAR of 2.1 were employed.

MelP, a human melanoma cell line, is abundant in the membrane form of PRAME [33,53]. In the MTT assay, the viability of the cells treated with the MMAE-based conjugate **32** significantly decreased (IC_50_ = 47 nM), whereas the unmodified 6H8 antibody did not exhibit any toxicity (Figure 7A). Additionally, we tested the cytotoxicity of free MMAE and the sCy3-MMAE conjugate **31** (IC_50_ < 15.6 nM for both). Conjugate **31** exhibited a lower cytotoxicity compared to MMAE (Figure 7A), probably due to its decreased ability to penetrate the cell membrane because of the charged cyanine moiety. 

On the contrary, treatment of cells with doxorubicin conjugate **14** in concentrations of up to 500 nM did not result in any cell death, while the IC_50_ of free doxorubicin was determined to be 0.17 µM. Doxorubicin-dye conjugate **10** did not exhibit any toxic effects at the concentrations tested due to the added charge (Figure 7B).

Similar effects were observed in the case of the PRAME (+) THP-1 line. A notable reduction in cell viability was observed for conjugates **32** (IC_50_ = 46 nM) and **31** (IC_50_ = 33 nM), as well as free MMAE (IC_50_ < 15.6 nM) (Figure 8A). Doxorubicin-based conjugates **10** and **14** proved ineffectual, while free doxorubicin exhibited rather potent cytotoxicity (IC_50_ = 44 nM) (Figure 8B). 

In the case of the murine colorectal carcinoma CT26 cell line, (Figure 9A,B), which does not express PRAME in the membrane or cytosol form, no decrease in viability was observed after treatment with either conjugate **32** (Figure 9A) or **14** (Figure 9B). In this case, the only agents to exhibit cytotoxicity were MMAE (IC_50_ = 22.4 nM), doxorubicin (IC_50_ = 0.19 µM), and conjugate 31 (0.49 µM).

Thus, we observed specific activity of conjugate **32** against PRAME-positive cells at nanomolar concentrations. This result is consistent with previously described MMAE-based ADCs to other tumor antigens [47,54,55]. Importantly, doxorubicin conjugate **14** showed no activity against these cell lines at concentrations of up to 0.5 µM. Based on the fact that conjugates **32** and **14** had comparable affinity and DAR values, we can conclude that the lack of the cytotoxic effect is due to doxorubicin not being toxic enough for use in anti-PRAME ADC preparations.

## 3. Materials and Methods

### 3.1. General Methods

All reactions were carried out under an argon atmosphere and anhydrous conditions with dry solvents, unless otherwise noted. Dimethylformamide (DMF) was purified by distillation with benzene/water, followed by vacuum distillation over CaH_2_, and stored over 3 Å molecular sieves. Dichloromethane (DCM) and methanol (MeOH) were purified by distillation, dimethyl sulfoxide (DMSO) was purified by vacuum distillation and stored over 3 Å molecular sieves. Compounds **4** [35], **15** [56], **19** [35], **20** [57], **23** [58], **26** [57], **28** [31], and **29** [56] were prepared as described previously. Yields refer to chromatographically and spectroscopically (^1^H and ^13^C NMR) homogeneous materials, unless otherwise stated. Reagents were of the highest commercially available quality and were used without further purification, unless otherwise stated. Reactions were monitored by thin-layer chromatography (TLC) carried out on 0.20 mm Merck silica gel plates (60 F_254_) using UV light as the visualizing agent and an alkaline aqueous solution of KMnO_4_ and heat as developing agents. Merck Kieselgel 60 was used for flash column chromatography. Size exclusion chromatography was performed using Sephadex G10 and G50 (Pharmacia Fine Chemicals) and Biogel P100 (Bio-Rad). UV–Vis absorbance spectra were recorded on a Cary 100 UV–Visible spectrophotometer. NMR spectra were recorded on Bruker 700 and 800 MHz instruments and calibrated using residual solvent as the internal reference (for CDCl_3_: ^1^H, δ 7.26 ppm, and ^13^C, δ 77.16 ppm; for DMSO-*d*_6_: δ 2.50 ppm and ^13^C, δ 39.52 ppm), unless otherwise noted. The following abbreviations were used to explain the multiplicities: s = singlet, d = doublet, t = triplet, q = quartet, ABq = AB quartet, quint = quintet, m = multiplet, app = apparent, br = broad. High-resolution mass spectra (HRMS) were recorded on a Thermo Scientific Orbitrap Exactive mass spectrometer using ESI (Electrospray ionization). Supplementary Materials.

### 3.2. Synthetic Procedures 

#### 3.2.1. Diazide **24**

A solution of TSTU (106 mg, 0.353 mmol, 1.2 equiv) in a DMF–1,4-dioxane–H_2_O mixture (2:2:1 *v*/*v*/*v*; 6.6 mL) was added dropwise to a solution of salt **22** (244 mg, 0.294 mmol, 1.0 equiv) and DIPEA (76 mg, 0.102 mL, 0.588 mmol, 2.0 equiv) in DMF–1,4-dioxane–H_2_O (2:2:1 *v*/*v*/*v*; 6.6 mL). The reaction mixture was stirred at 23 °C for 3 h, then a solution of aminoazide **23** (96 mg, 0.440 mmol, 1.5 equiv) in DMF–1,4-dioxane–H_2_O (2:2:1 *v*/*v*/*v*; 6.6 mL) was added dropwise. The reaction mixture was stirred for 20 h, after which it was concentrated under reduced pressure until its volume was brought down to 8 mL. The mixture was then added dropwise to stirred EtOAc (80 mL), with an amorphous magenta precipitate forming. The resultant mixture was passed through a pad of Celite, the precipitate was washed with EtOAc, dried, eluted with MeOH (150 mL), and concentrated. The resultant residue was purified by flash column chromatography (silica gel, DCM–MeOH–H_2_O–Et_3_N, 283:17.5:2:3 → 184:17.5:2:2 → 85:17.5:2:1), then dissolved in H_2_O and purified on a Sephadex G10 column. The resultant solution was passed through a Dowex 50WX4 column in Et_3_NH^+^ form and concentrated. Diazide **24** was obtained as a dark magenta amorphous solid (50 mg, 14%). *R*_f_ 0.51 (silica gel, DCM–MeOH–H_2_O–Et_3_N, 85:15:1:4). ^1^H NMR (700 MHz, CDCl_3_) δ 9.99 (br s, 1 H), 8.39 (t, *J* = 13.3 Hz, 1H), 7.97–7.91 (m, 4H), 7.12 (d, *J* = 8.3 Hz, 2H), 6.80 (t, *J* = 5.6 Hz, 2H), 6.48 (d, *J* = 13.3 Hz, 2H), 4.14–4.02 (m, 4H), 3.67–3.56 (m, 20H), 3.52 (t, *J* = 5.6 Hz, 4H), 3.38 (q, *J* = 5.6 Hz, 4H), 3.35 (t, *J* = 5.0 Hz, 4H), 3.20 (q, *J* = 7.3 Hz, 6H), 2.20 (t, *J* = 7.4 Hz, 4H), 1.86–1.77 (m, 4H), 1.72–1.66 (m, 4H), 1.70 (s, 12H), 1.52–1.44 (m, 4H), 1.37 (t, *J* = 7.3 Hz, 9H). ^13^C NMR (201 MHz, CDCl_3_) δ 174.9, 173.2, 150.9, 144.4, 142.6, 140.3, 127.5, 120.7, 110.7, 103.8, 70.7, 70.7, 70.6, 70.3, 70.1, 69.8, 50.8, 49.5, 46.6, 44.7, 39.2, 36.1, 28.2, 27.3, 26.4, 25.3, 9.0. HRMS (ESI) calcd for C_51_H_75_N_10_O_14_S_2_^−^ [M−Et_3_NH]^−^ 1115.4911, found 1115.4904.

#### 3.2.2. Conjugate **6**

In a 1.5 mL polypropylene microcentrifuge tube with a screw cap, a solution of carbonate **15** (13.7 mg, 0.0215 mmol, 1.0 equiv) in dry DMF (215 µL) was added to doxorubicin hydrochloride (16.0 mg, 0.0276 mmol, 1.3 equiv). DIPEA (4.5 mg, 6.0 µL, 0.0345 mmol, 1.6 equiv) was then added, the tube was purged with argon, and the solution was vortexed vigorously until most of the doxorubicin hydrochloride dissolved. The reaction mixture was agitated on an orbital shaker at 23 °C for 15 h. The reaction mixture was diluted with EtOAc (2.8 mL), and the resultant red precipitate was separated by centrifugation. The precipitate was washed with EtOAc using sonication and centrifugation (2 × 2.8 mL). The EtOAc supernatants were stored at −18 °C for 30 min, at which point the additionally formed precipitate was separated by centrifugation. The combined precipitate was purified by flash column chromatography (silica gel, DCM–MeOH, 20:1 → 10:1 → 5:1) to afford conjugate **6** (10.4 mg, 46%) as a dark red solid. *R*_f_ 0.40 (silica gel, DCM–MeOH, 5:1). ^1^H NMR (700 MHz, DMSO-*d*_6_) δ 14.05 (s, 1H), 13.29 (s, 1H), 9.92 (s, 1H), 8.04 (d, *J* = 7.6 Hz, 1H), 7.96–7.90 (m, 2H), 7.84 (d, *J* = 8.6 Hz, 1H), 7.67 (dd, *J* = 7.4, 2.3 Hz, 1H), 7.55 (d, *J* = 8.2 Hz, 2H), 7.24 (d, *J* = 8.1 Hz, 2H), 6.79 (d, *J* = 8.0 Hz, 1H), 5.94 (t, *J* = 6.2 Hz, 1H), 5.45 (s, 1H), 5.37 (s, 2H), 5.22 (d, *J* = 3.7 Hz, 1H), 4.97 (app t, *J* = 4.6 Hz, 1H), 4.89 (s, 2H), 4.81 (t, *J* = 5.9 Hz, 1H), 4.67 (d, *J* = 5.7 Hz, 1H), 4.57 (d, *J* = 6.1 Hz, 2H), 4.38–4.34 (m, 1H), 4.18 (dd, *J* = 8.6, 6.8 Hz, 1H), 4.16–4.13 (m, 1H), 4.00 (s, 3H), 3.75–3.69 (m, 1H), 3.44 (m, 1H), 3.04–2.96 (m, 3H), 2.96–2.89 (m, 1H), 2.76 (t, *J* = 2.7 Hz, 1H), 2.26 (dq, *J* = 17.0, 7.2 Hz, 2H), 2.20 (dm, *J* = 14.3 Hz, 1H), 2.17–2.11 (m, 3H), 1.97 (app sextet, *J* = 6.8 Hz, 1H), 1.84 (td, *J* = 13.0, 4.0 Hz, 1H), 1.71–1.62 (m, 3H), 1.61–1.53 (m, 1H), 1.48 (dd, *J* = 12.3, 4.5 Hz, 1H), 1.46–1.38 (m, 1H), 1.38–1.31 (m, 1H), 1.12 (d, *J* = 6.4 Hz, 3H), 0.85 (d, *J* = 6.8 Hz, 3H), 0.82 (d, *J* = 6.8 Hz, 3H). ^13^C NMR (176 MHz, DMSO-*d*_6_) δ 213.8, 186.2, 186.1, 171.8, 171.2, 170.5, 160.7, 158.9, 156.0, 155.3, 154.5, 138.5, 136.0, 135.3, 134.4, 133.9, 131.8, 128.4, 119.8, 119.5, 118.9, 118.8, 110.6, 110.4, 100.3, 84.1, 74.9, 71.3, 69.7, 68.0, 66.7, 64.9, 63.7, 57.8, 56.5, 53.1, 47.1, 38.5, 36.4, 34.0, 32.0, 30.3, 29.8, 29.2, 26.7, 24.4, 19.2, 18.1, 17.4, 17.0. HRMS (ESI) calcd for C_52_H_63_N_6_O_17_^+^ [M+H]^+^ 1043.4244, found 1043.4246.

#### 3.2.3. Conjugate **2**

In a 1.5 mL polypropylene microcentrifuge tube with a screw cap, cyanine **4** (7.4 mg, 5.8 μmol, 1.0 equiv) and conjugate **6** (6.1 mg, 5.8 μmol, 1.0 equiv) were dissolved in dry DMSO (578 μL). Sodium ascorbate (284 mM in H_2_O, 102 μL, 28.9 μmol, 5.0 equiv) was added, and the solution was degassed by carefully bubbling argon through it for 1 min. Then, a solution containing CuSO_4_ and TBTA (DMSO–H_2_O 11:10, 10 mM CuSO_4_, 11 mM TBTA, 580 μL, 5.8 μmol, 1.0 equiv CuSO_4_) was added, the tube was purged with argon and closed, and the reaction mixture was vortexed vigorously and agitated on an orbital shaker at 23 °C for 23 h. After that, the mixture was poured into 11.5 mL of EtOAc, which resulted in a biphasic mixture. The layers were separated by centrifugation and decantation. The aqueous layer was washed with another 11.5 mL of EtOAc, yielding a dark magenta precipitate. The precipitate was washed with 11.5 mL EtOAc using sonication, separated by centrifugation, dried in vacuo and purified by flash column chromatography (silica gel, DCM–MeOH–H_2_O–Et_3_N, 283:17.5:2:3 → 184:17.5:2:2 → 85:17.5:2:1) to afford conjugate **2** (7.0 mg, 52%) as a dark magenta solid. *R*_f_ 0.44 (silica gel, DCM–MeOH–H_2_O–Et_3_N, 85:15:1:4). ^1^H NMR (700 MHz, DMSO-*d*_6_) δ 14.03 (s, 1H), 13.27 (br s, 1H), 9.92 (s, 1H), 8.35 (t, *J* = 13.4 Hz, 1H), 8.06 (d, *J* = 7.5 Hz, 2H), 7.93–7.86 (m, 2H), 7.83 (d, *J* = 8.5 Hz, 1H), 7.82–7.77 (m, 5H), 7.68 (dt, *J* = 8.1, 1.9 Hz, 2H), 7.66–7.63 (m, 1H), 7.55 (d, *J* = 8.0 Hz, 2H), 7.39 (d, *J* = 8.4 Hz, 2H), 7.23 (d, *J* = 8.1 Hz, 2H), 6.79 (d, *J* = 8.1 Hz, 1H), 6.51 (d, *J* = 13.2 Hz, 2H), 6.01–5.95 (m, 1H), 5.45 (s, 1H), 5.39 (s, 2H), 5.22 (d, *J* = 3.3 Hz, 1H), 4.95 (app t, *J* = 4.4 Hz, 1H), 4.88 (s, 2H), 4.82 (br s, 1H), 4.68 (d, *J* = 5.9 Hz, 1H), 4.57 (s, 2H), 4.44 (t, *J* = 5.3 Hz, 2H), 4.36 (q, *J* = 7.7 Hz, 1H), 4.20 (app t, *J* = 7.7 Hz, 1H), 4.15 (q, *J* = 6.6 Hz, 1H), 4.13–4.06 (m, 4H), 3.98 (s, 3H), 3.93 (q, *J* = 7.0 Hz, 2H), 3.91 (app t, *J* = 4.9 Hz, 2H), 3.77 (t, *J* = 5.4 Hz, 2H), 3.74–3.69 (m, 1H), 3.57 (app t, *J* = 4.9 Hz, 2H), 3.51–3.46 (m, 9H), 3.46–3.43 (m, 8H), 3.38–3.34 (m, 6H), 3.18–3.13 (m, 4H), 3.08–3.01 (m, 10 H), 3.01–2.96 (m, 3H), 2.95–2.89 (m, 1H), 2.58 (dd, *J* = 8.8, 6.4 Hz, 2H), 2.28–2.17 (m, 3H), 2.12 (dd, *J* = 14.1, 5.9 Hz, 1H), 2.09–2.03 (m, 4H), 1.97 (app sextet, *J* = 6.7 Hz, 1H), 1.85 (s, 3H), 1.83–1.77 (m, 3H), 1.75–1.65 (m, 17H), 1.62–1.51 (m, 5H), 1.48 (dm, *J* = 11.4 Hz, 1H), 1.46–1.40 (m, 1H), 1.40–1.32 (m, 5H), 1.19 (t, *J* = 7.1 Hz, 3H), 1.16 (t, *J* = 7.2 Hz, 15H), 1.12 (d, *J* = 6.5 Hz, 3H), 0.85 (d, *J* = 6.7 Hz, 3H), 0.82 (d, *J* = 6.8 Hz, 3H). ^13^C NMR (201 MHz, DMSO-*d*_6_) δ 213.7, 186.6, 186.5, 174.2, 172.1, 172.0, 171.2, 170.5, 161.6, 160.8, 158.8, 156.1, 155.3, 154.5, 149.9, 146.3, 145.83, 145.78, 141.8, 140.1, 138.5, 136.2, 135.6, 134.7, 134.1, 131.7, 128.5, 126.2, 122.1, 120.1, 119.83, 119.76, 119.0, 118.9, 110.8, 110.7, 102.9, 100.2, 75.0, 72.3, 69.8, 69.74, 69.69, 69.64, 69.56, 69.53, 69.50, 69.1, 68.7, 68.4, 68.0, 66.7, 64.9, 63.6, 61.8, 57.6, 56.6, 53.1, 49.1, 48.9, 47.1, 45.7, 43.8, 38.5, 38.4, 36.7, 35.0, 34.6, 32.1, 30.3, 29.8, 29.2, 27.4, 26.7, 25.73, 25.71, 25.4, 24.9, 24.6, 19.2, 18.1, 17.0, 14.2, 13.4, 8.8. HRMS (ESI) calcd for C_107_H_144_N_14_O_33_S_2_^2−^ [M−Et_3_NH−H]^2−^ 1108.4736, found 1108.4763.

#### 3.2.4. Conjugate **10**

In a 1.5 mL polypropylene microcentrifuge tube with a screw cap, diazide **24** (6.6 mg, 5.4 μmol, 2.5 equiv) was dissolved in dry DMSO (200 μL), and sodium ascorbate (284 mM in H_2_O, 38 μL, 11 μmol, 5.0 equiv) was added. In a separate tube, conjugate **6** (2.3 mg, 2.2 μmol, 1.0 equiv) was dissolved in dry DMSO (100 μL), and 20 μL of the solution were added to the reaction mixture. Then, a solution containing CuSO_4_ and TBTA (DMSO–H_2_O 11:10, 10 mM CuSO_4_, 11 mM TBTA, 217 μL, 2.2 μmol, 1.0 equiv CuSO_4_) was added, the reaction mixture was vortexed vigorously and agitated on an orbital shaker at 23 °C for 1 h. As TLC analysis indicated complete consumption of **6**, 20 μL of its DMSO solution were added to the reaction. After 20 min, the rest of the solution was added in portions (6×10 μL) over the course of 20 min. After additional 30 min, the mixture was poured into 6.4 mL of EtOAc, which resulted in a biphasic mixture. The layers were separated by centrifugation and decantation. The aqueous layer was washed with another 6.4 mL of EtOAc, which resulted in a dark magenta precipitate. The precipitate was washed with 6.4 mL EtOAc using sonication, separated by centrifugation, dried in vacuo, and purified by flash column chromatography (silica gel, DCM–MeOH–H_2_O–Et_3_N, 283:17.5:2:3 → 184:17.5:2:2) to afford conjugate **10** (2.2 mg, 44%) as a dark magenta solid. *R*_f_ 0.40 (silica gel, DCM–MeOH–H_2_O–Et_3_N, 85:15:1:4). ^1^H NMR (700 MHz, DMSO-*d*_6_) δ 14.02 (s, 1H), 13.27 (s, 1H), 9.92 (s, 1H), 9.23 (br s, 1H), 8.35 (t, *J* = 13.4 Hz, 1H), 8.06 (d, *J* = 7.6 Hz, 1H), 7.94–7.88 (m, 2H), 7.83 (d, *J* = 8.5 Hz, 1H), 7.82–7.76 (m, 5H), 7.68 (dt, *J* = 8.2, 1.9 Hz, 2H), 7.67–7.63 (m, 1H), 7.55 (d, *J* = 8.2 Hz, 2H), 7.39 (d, *J* = 8.2 Hz, 2H), 7.23 (d, *J* = 8.2 Hz, 2H), 6.79 (d, *J* = 8.0 Hz, 1H), 6.51 (d, *J* = 13.3 Hz, 2H), 5.98 (t, *J* = 5.6 Hz, 1H), 5.44 (s, 1H), 5.39 (s, 2H), 5.22 (d, *J* = 3.6 Hz, 1H), 4.95 (app t, *J* = 4.6 Hz, 1H), 4.89 (ABq, Δδ_AB_ = 0.00, *J*_AB_ = 13.0 Hz, 2H), 4.82 (t, *J* = 5.9 Hz, 1H), 4.68 (d, *J* = 5.6 Hz, 1H), 4.57 (d, *J* = 5.8 Hz, 2H), 4.44 (t, *J* = 5.3 Hz, 2H), 4.37 (app q, *J* = 7.7 Hz, 1H), 4.20 (dd, *J* = 8.5, 6.7 Hz, 1H), 4.15 (q, *J* = 6.7 Hz, 1H), 4.11 (br t, *J* = 6.9 Hz, 4H), 3.98 (s, 3H), 3.77 (t, *J* = 5.3 Hz, 2H), 3.75–3.69 (m, 1H), 3.58 (app t, *J* = 5.0 Hz, 2H), 3.55–3.47 (m, 10H), 3.47–3.42 (m, 7H), 3.39–3.34 (m, 6H), 3.16 (app quint, *J* = 5.9 Hz, 4H), 3.08 (q, *J* = 7.3 Hz, 9H), 3.03–2.95 (m, 3H), 2.95–2.89 (m, 1H), 2.58 (dd, *J* = 8.8, 6.5 Hz, 2H), 2.28–2.17 (m, 3H), 2.12 (dd, *J* = 14.2, 5.6 Hz, 1H), 2.07 (t, *J* = 7.3 Hz, 2H), 2.06 (t, *J* = 7.4 Hz, 2H), 1.97 (app sextet, *J* = 6.7 Hz, 1H), 1.87–1.77 (m, 3H), 1.76–1.64 (m, 17H), 1.62–1.52 (m, 5H), 1.48 (dd, *J* = 11.8, 3.7 Hz, 1H), 1.46–1.40 (m, 1H), 1.36 (s, 7H), 1.18 (t, *J* = 7.3 Hz, 14H), 1.12 (d, *J* = 6.4 Hz, 3H), 0.85 (d, *J* = 6.8 Hz, 3H), 0.83 (d, *J* = 6.8 Hz, 3H). ^13^C NMR (201 MHz, DMSO-*d*_6_) δ 213.7, 186.6, 186.5, 174.2, 172.1, 171.95, 171.94, 171.2, 170.5, 160.8, 158.8, 156.1, 155.3, 154.5, 149.9, 146.3, 145.84, 145.79, 141.80, 141.78, 140.1, 138.5, 136.2, 135.6, 134.7, 134.1, 131.7, 128.5, 126.2, 122.1, 120.1, 119.84, 119.75, 119.0, 118.9, 110.8, 110.7, 102.9, 100.2, 75.0, 69.8, 69.74, 69.72, 69.64, 69.57, 69.51, 69.50, 69.2, 69.113, 69.105, 68.7, 68.0, 66.7, 64.9, 63.6, 57.64, 56.58, 53.1, 50.0, 49.1, 48.9, 47.1, 45.7, 43.8, 38.5, 38.4, 36.7, 35.0, 34.6, 32.1, 30.3, 29.8, 29.2, 27.4, 26.7, 25.7, 25.4, 24.9, 24.6, 19.2, 18.1, 17.0, 8.6. HRMS (ESI) calcd for C_103_H_136_N_16_O_31_S_2_^2−^ [M−Et_3_NH−H]^2−^ 1078.4505, found 1078.4528.

#### 3.2.5. Imidate **8**

Mesylate **20** (1.60 g, 5.16 mmol, 1.0 equiv) was dissolved in a mixture of i-PrOH and t-BuOH (1:1 *v*/*v*, 240 mL), and sodium salt of ethyl N-hydroxyacetimidate (3.23 g, 25.8 mmol, 5.0 equiv) was added in portions. The mixture was refluxed for 2 h, then cooled to 23 °C. The resultant precipitate was filtered off and the solvent was removed under reduced pressure. The residue was purified by flash column chromatography (silica gel, DCM–MeOH, 50:1). Imidate **8** was isolated as a colorless oil (600 mg, 37%). *R*_f_ 0.40 (silica gel, DCM–MeOH, 20:1). ^1^H NMR (700 MHz, CDCl_3_) δ 4.19 (d, *J* = 2.4 Hz, 2H), 4.03 (app t, *J* = 4.9 Hz, 1H), 3.99 (q, *J* = 7.0 Hz, 2H), 3.71–3.63 (m, 14H), 2.41 (t, *J* = 2.4 Hz, 1H), 1.93 (s, 1H), 1.25 (t, *J* = 7.1 Hz, 2H). ^13^C NMR (176 MHz, CDCl_3_) δ 162.8, 79.8, 74.6, 72.9, 70.79, 70.78, 70.74, 70.72, 70.6, 69.6, 69.3, 62.3, 58.5, 14.5, 13.9. HRMS (ESI) calcd for C_15_H_28_NO_6_^+^ [M+H]^+^ 318.1911, found 318.1912.

#### 3.2.6. Alkyne **27**

A solution of TSTU (104 mg, 0.346 mmol, 1.2 equiv) in a DMF:1,4-dioxane:H_2_O mixture (2:2:1 *v*/*v*/*v*; 6.4 mL) was added dropwise to a solution of salt **25** (246 mg, 0.287 mmol, 1.0 equiv) and DIPEA (74 mg, 100 µL, 0.575 mmol, 2.0 equiv) in DMF–1,4-dioxane:H_2_O (2:2:1 *v*/*v*/*v*; 6.4 mL). The reaction was stirred at 23 °C for 1 h, then a solution of aminoalkyne **26** (100 mg, 0.433 mmol, 1.5 equiv) in DMF–1,4-dioxane–H_2_O (2:2:1 *v*/*v*/*v*; 6.4 mL) was added dropwise. The reaction mixture was stirred for 2.5 h, after which it was concentrated under reduced pressure until its volume was brought down to 8 mL. The mixture was then added dropwise to stirred EtOAc (80 mL), upon which an amorphous blue precipitate was formed. The resultant mixture was passed through a pad of Celite, the precipitate was washed with EtOAc, dried, eluted with MeOH (150 mL), and concentrated. The resultant residue was purified by flash column chromatography (silica gel, DCM–MeOH–H_2_O–Et_3_N, 283:17.5:2:3 → 184:17.5:2:2 → 85:17.5:2:1), then dissolved in H_2_O and purified on a Sephadex G10 column. The resultant solution was passed through a Dowex 50WX4 column in Et_3_NH^+^ form and concentrated. Alkyne **27** was obtained as a dark blue amorphous solid (142 mg, 43%). *R*_f_ 0.26 (silica gel, DCM–MeOH–H_2_O–Et_3_N, 85:15:1:4). ^1^H NMR (700 MHz, DMSO-*d*_6_) δ 8.35 (t, *J* = 13.0 Hz, 2H), 7.83–7.79 (m, 3H), 7.64 (dd, *J* = 8.1, 1.6 Hz, 2H), 7.32 (dd, *J* = 8.3, 5.1 Hz, 2H), 6.60 (t, *J* = 12.3 Hz, 1H), 6.30 (dd, *J* = 13.8, 4.4 Hz, 2H), 4.11–4.04 (m, 4H), 3.55–3.46 (m, 16H), 3.39 (t, *J* = 2.4 Hz, 1H), 3.37 (t, *J* = 6.0 Hz, 2H), 3.17 (q, *J* = 5.9 Hz, 2H), 3.05 (q, *J* = 7.2 Hz, 12H), 2.13 (t, *J* = 7.3 Hz, 2H), 2.06 (t, *J* = 7.3 Hz, 2H), 1.73–1.65 (m, 16H), 1.53 (quint, *J* = 7.4 Hz, 4H), 1.40–1.30 (m, 4H), 1.17 (t, *J* = 7.2 Hz, 18H). ^13^C NMR (201 MHz, DMSO-*d*_6_) δ 174.3, 172.98, 172.96, 171.9, 154.2, 145.28, 145.25, 141.95, 141.93, 140.48, 140.46, 135.2, 126.0, 125.9, 119.9, 116.2, 110.1, 103.5, 103.4, 80.3, 77.0, 71.0, 69.72, 69.69, 69.53, 69.46, 69.1, 69.0, 68.5, 57.5, 48.9, 45.7, 43.42, 43.38, 38.4, 35.0, 33.5, 27.1, 26.6, 25.7, 25.6, 24.8, 24.2, 8.8. HRMS (ESI) calcd for C_48_H_63_N_3_O_13_S_2_^2−^ [M−2Et_3_NH]^2−^ 476.6907, found 476.6909.

#### 3.2.7. Imidate **12**

To a solution of **27** (81 mg, 0.070 mmol, 1.0 equiv) and DIPEA (23 mg, 31 µL, 0.18 mmol, 2.5 equiv) in DMF (1.4 mL), TSTU (28 mg, 0.094 mmol, 1.3 equiv) was added. The reaction mixture was stirred for 20 min, then a solution of aminoimidate **28** (29 mg, 0.10 mmol, 1.5 equiv) in DMF (0.30 mL) was added. The reaction mixture was stirred for 2 h, then the mixture was added dropwise to stirred EtOAc (20 mL), upon which an amorphous blue precipitate was formed. The resultant mixture was passed through a pad of Celite, the precipitate was washed with EtOAc, dried, eluted with MeOH (50 mL) and concentrated. The resultant residue was purified by flash column chromatography (silica gel, DCM–MeOH–H_2_O–Et_3_N, 283:17.5:2:3 → 184:17.5:2:2), then dissolved in H_2_O and purified on a Sephadex G10 column. The solution was passed through a Dowex 50WX4 column in Et_3_NH^+^ form and concentrated. The desired compound **12** was obtained as a dark blue amorphous solid (49 mg, 53%). *R*_f_ 0.25 (silica gel, DCM–MeOH–H_2_O–Et_3_N, 85:17.5:2:1). ^1^H NMR (700 MHz, CDCl_3_) δ 10.15 (s, 1H), 7.97–7.87 (m, 6H), 7.08 (dd, *J* = 8.4, 4.0 Hz, 2H), 6.72–6.66 (m, 1H), 6.66–6.57 (m, 2H), 6.60 (t, *J* = 11.4 Hz, 1H), 6.11 (d, *J* = 13.4 Hz, 2H), 4.17 (d, *J* = 2.4 Hz, 1H), 4.01 (app t, *J* = 8.1 Hz, 2H), 4.00–3.95 (m, 4H), 3.97 (q, *J* = 7.0 Hz, 2H), 3.69–3.66 (m, 4H), 3.66–3.56 (m, 18H), 3.53 (t, *J* = 5.5 Hz, 4H), 3.40 (q, *J* = 5.5 Hz, 4H), 3.20 (q, *J* = 7.4 Hz, 6H), 2.45 (t, *J* = 2.4 Hz, 1H), 2.21 (app t, *J* = 7.2 Hz, 4H), 1.90 (s, 3H), 1.82–1.73 (m, 4H), 1.80–1.72 (m, 4H), 1.75 (s, 12H), 1.45 (t, *J* = 7.3 Hz, 9H), 1.24 (t, *J* = 7.1 Hz, 2H). ^13^C NMR (176 MHz, CDCl_3_) δ 173.5, 173.0, 172.9, 162.7, 153.6, 144.2, 142.6, 140.7, 127.3, 120.7, 117.2, 110.2, 103.8, 79.7, 74.8, 72.8, 70.61, 70.58, 70.5, 70.4, 70.2, 69.8, 69.4, 69.2, 62.2, 58.4, 49.5, 46.6, 44.5, 39.2, 36.0, 28.1, 27.1, 26.6, 25.1, 14.5, 13.8, 8.9. HRMS (ESI) calcd for C_60_H_88_N_5_O_17_S_2_^−^ [M−Et_3_NH]^−^ 1214.5622, found 1214.5613.

#### 3.2.8. Imidate **30**

In a 1.5 mL polypropylene microcentrifuge tube with a screw cap, cyanine **4** (2.9 mg, 2.3 μmol, 1.2 equiv) and compound **29** (2.3 mg, 1.9 μmol, 1.0 equiv) were dissolved in dry DMSO (300 μL). An aqueous solution of the CuSO_4_·THPTA 1:1 complex (0.099 mM, 9.5 μL, 0.95 μmol, 0.5 equiv) was added, followed by sodium ascorbate (404 mM in H_2_O, 24 μL, 9.5 μmol, 5.0 equiv). The reaction mixture was vortexed vigorously and agitated on an orbital shaker. After 30 min, TLC indicated low conversion, and additional sodium ascorbate was introduced (404 mM in H_2_O, 24 μL, 9.5 μmol, 5.0 equiv). Then, 10 min later, TLC indicated substantial conversion; more sodium ascorbate was added (404 mM in H_2_O, 24 μL, 9.5 μmol, 5.0 equiv), and the reaction was agitated on an orbital shaker at 23 °C for 20 h. After that, the product was precipitated by treating 100 μL aliquots of the reaction mixture with 1.80 mL EtOAc. The resultant residue was redissolved in 50 μL of DMSO and precipitated with 1.80 mL EtOAc. The precipitate was washed with 1.80 mL EtOAc, dried in air and dissolved in 1 mL of 4:1 (*v*/*v*) DCM-MeOH mixture to form a turbid solution. Pale pink precipitate (presumably sodium ascorbate stained with product) was separated by centrifugation, and the solution was loaded onto a silica gel column. Purification by flash column chromatography (silica gel, DCM–MeOH–H_2_O–Et_3_N, 283:17.5:2:3 → 184:17.5:2:2) yielded a solid residue, which was then dissolved in H_2_O and purified on a Sephadex G10 column. The resultant solution was passed through a Dowex 50WX4 column in Et_3_NH^+^ form and concentrated. Compound **30** (1.9 mg, 40%) was obtained as a dark magenta solid. *R*_f_ 0.48 (silica gel, DCM–MeOH–H_2_O–Et_3_N, 85:15:1:4). ^1^H NMR (700 MHz, DMSO-*d*_6_) δ 9.98–9.91 (m, 1H), 8.36 (t, *J* = 13.4 Hz, 1H), 8.28–8.21 (m, 0.4H), 8.08 (d, *J* = 7.5 Hz, 1H), 8.02–7.95 (m, 0.4H), 7.88–7.76 (m, 6H), 7.68 (ddd, *J* = 8.1, 2.7, 1.6 Hz, 2H), 7.61–7.55 (m, 2.5H), 7.39 (d, *J* = 8.3 Hz, 2H), 7.35–7.23 (m, 6H), 7.19–7.13 (m, 1H), 6.51 (d, *J* = 13.4 Hz, 2H), 6.01–5.94 (m, 1H), 5.42–5.36 (m, 2.5H), 5.32 (d, *J* = 4.9 Hz, 0.5H), 5.13–4.93 (m, 2H), 4.73 (br s, 0.5H), 4.63 (br s, 0.4H), 4.49 (t, *J* = 5.4 Hz, 1H), 4.44 (t, *J* = 5.3 Hz, 2H), 4.46–4.36 (m, 2H), 4.36–4.30 (m, 1.6H), 4.26 (dd, *J* = 19.9, 10.8 Hz, 1H), 4.21 (dd, *J* = 8.6, 6.7 Hz, 1H), 4.11 (br t, *J* = 7.5 Hz, 4H), 4.09–4.05 (m, 1H), 4.04–3.95 (m, 2H), 3.93 (q, *J* = 7.0 Hz, 2H), 3.92–3.90 (m, 2H), 3.77 (t, *J* = 5.4 Hz, 2H), 3.80–3.76 (m, 0.5H), 3.63–3.53 (m, 3H), 3.52–3.41 (m, 20H), 3.38–3.34 (m, 3H), 3.24 (s, 2H), 3.23 (s, 2H), 3.20 (s, 2H), 3.19–3.14 (m, 8H), 3.11 (s, 2H), 3.06 (br q, *J* = 7.3 Hz, 9H), 3.03–2.90 (m, 2H), 2.89–2.82 (m, 3H), 2.60–2.57 (m, 2H), 2.31–2.17 (m, 3H), 2.15–2.03 (m, 2H), 2.07 (t, *J* = 7.3 Hz, 2H), 2.06 (t, J = 7.3 Hz, 2H), 2.02–1.92 (m, 2H), 1.85 (s, 3H), 1.83–1.77 (m, 4H), 1.77–1.65 (m, 19H), 1.63–1.40 (m, 8H), 1.40–1.27 (m, 6H), 1.19 (t, *J* = 7.1 Hz, 3H), 1.17 (t, *J* = 7.3 Hz, 13H), 1.06 (t, *J* = 7.0 Hz, 4H), 1.05–0.97 (m, 6H), 0.90–0.71 (m, 25H). HRMS (ESI) calcd for C_119_H_186_N_18_O_29_S_2_^2+^ [M−Et_3_N+2H]^2+^ 1197.6532, found 1197.6523.

#### 3.2.9. Conjugate **31**

In a 2 mL polypropylene microcentrifuge tube, to a solution of 0.5 mg of conjugate **30** in MeOH (50 μL), a solution of HCl in MeOH–H_2_O (5.5 μL, 0.12 M, 99:1 *v*/*v*) was added. The reaction mixture was vortexed and agitated on an orbital shaker for 10 min at 23 °C, after which the product was precipitated by the addition of 1.80 mL of Et_2_O. Upon centrifugation and an additional wash with 1.80 mL of Et_2_O, the product was dried in vacuo and used without further purification. *R*_f_ 0.39 (silica gel, DCM–MeOH–H_2_O–Et_3_N, 85:15:1:4). HRMS (ESI) calcd for C_115_H_180_N_18_O_28_S_2_ [M+2H]^2+^ 1162.6322, found 1162.6324.

### 3.3. Synthesis of Antibody Conjugates

#### 3.3.1. General Procedure A

To 200 μL of IgG solution in 20 mM acetate buffer (pH 5.0, 150 mM NaCl), aqueous NaIO_4_ (12 μL, 360 mM) was added. The mixture was agitated on an orbital shaker for 40 min at 23 °C in the dark, then aqueous glycerol (12 μL, 20% m/v) was added, and the reaction mixture was immediately loaded onto a Sephadex G-50 column equilibrated in 100 mM citrate buffer (pH 3.0, 150 mM NaCl); the target fraction was 500 μL. The concentration of oxidized antibody substance was measured spectrophotometrically, and ethoxyethylidene-protected oxyamine (20 equiv, 5–20 μL, solution in DMSO) was added to the antibody solution. The reaction was agitated on an orbital shaker for 1 h at 23 °C, after which the conjugate was purified on a Sephadex G-50 column equilibrated in PBS (pH 7.4); the target fraction volume was 1000 μL. The yield and stoichiometry of the conjugate were determined spectrophotometrically.

##### Conjugate **5**

**Figure 10:**
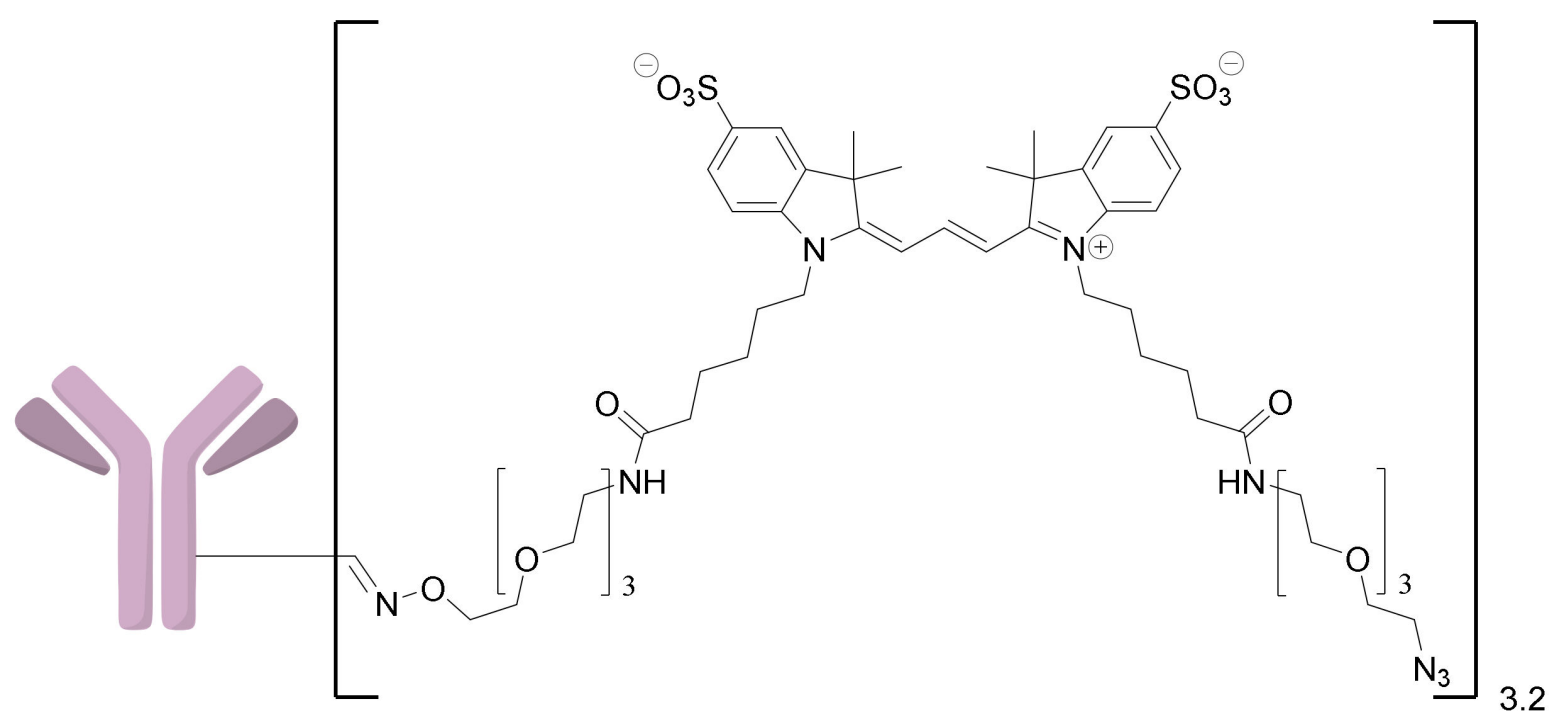
The structure of conjugate **5**.

The 6H8 antibody (1.50 mg, 10.0 nmol) was modified with reagent **4** according to general procedure A; 0.49 mg (3.3 nmol, 33%) of conjugate **5** (Figure 10) with an average degree of labeling of 3.2 were obtained.

##### Conjugate **9**

**Figure 11:**
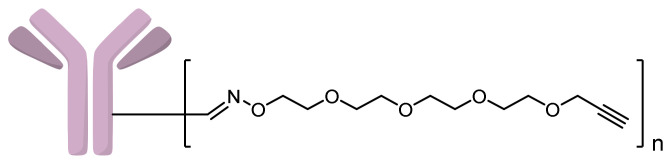
The structure of conjugate **9**.

The 6H8 antibody (0.90 mg, 6.0 nmol) was modified with reagent **8** according to general procedure A; 0.46 mg (3.1 nmol, 51%) of conjugate **9** (Figure 11) were obtained.

##### Conjugate **13**

**Figure 12:**
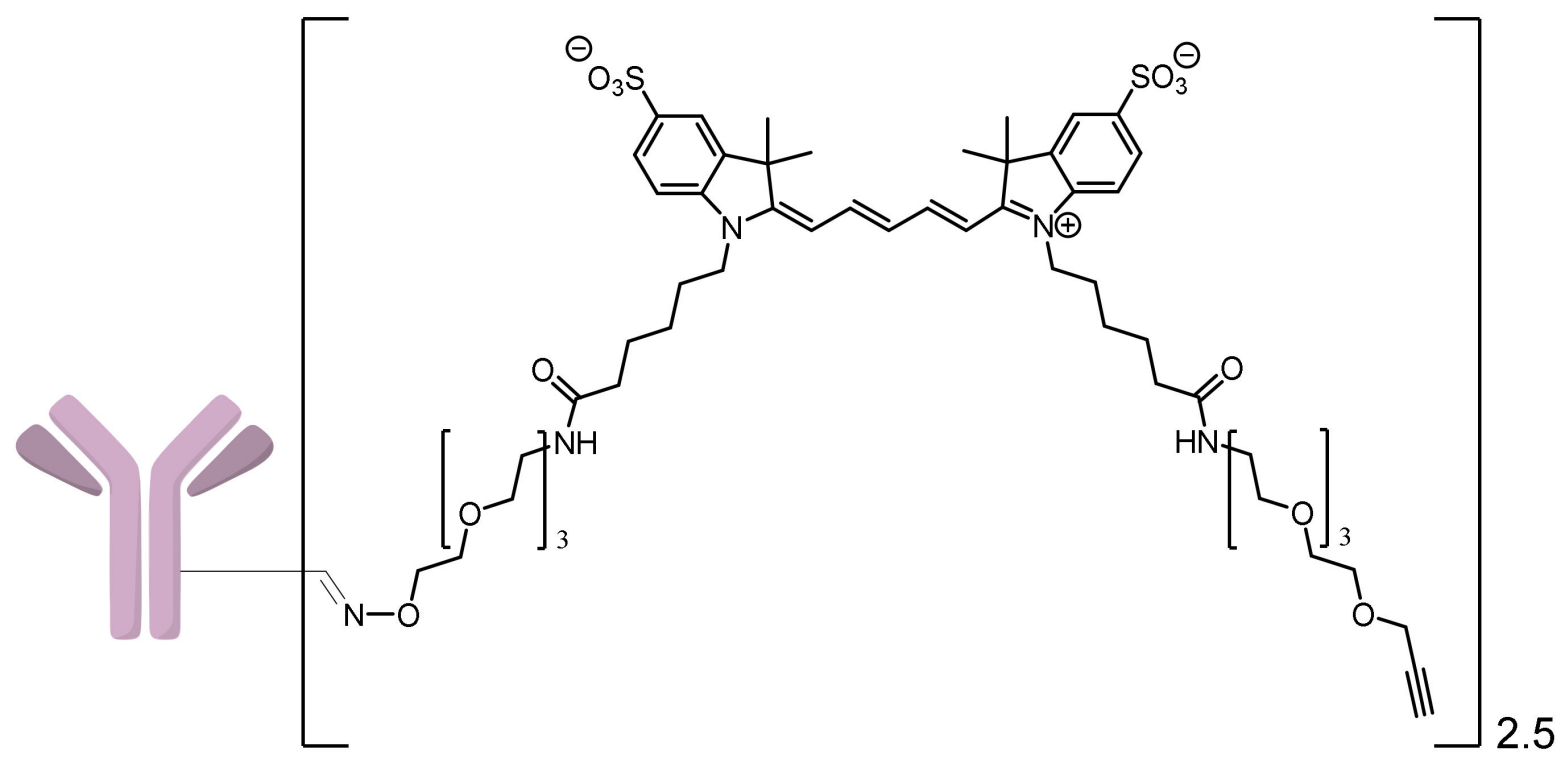
The structure of conjugate **13**.

The 6H8 antibody (1.07 mg, 7.1 nmol) was modified with reagent **12** according to general procedure A; 0.25 mg (1.7 nmol, 23%) of conjugate **13** (Figure 12) with an average degree of labeling of 2.5 were obtained.

##### Conjugate **3**

**Figure 13:**
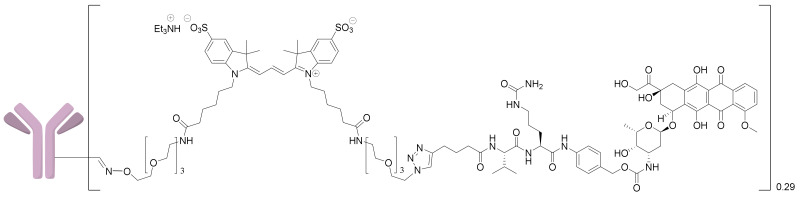
The structure of conjugate **3**.

The 6H8 antibody (1.79 mg, 11.9 nmol) was modified with reagent **2** according to modified general procedure A: reagent **2** was added in 8 portions of 0.5 equiv. every 10 min (total reaction time of 80 min); 0.54 mg (3.6 nmol, 30%) of conjugate **3** (Figure 13) were obtained with an average degree of labeling of 0.29.

##### Conjugate **32**

**Figure 14:**
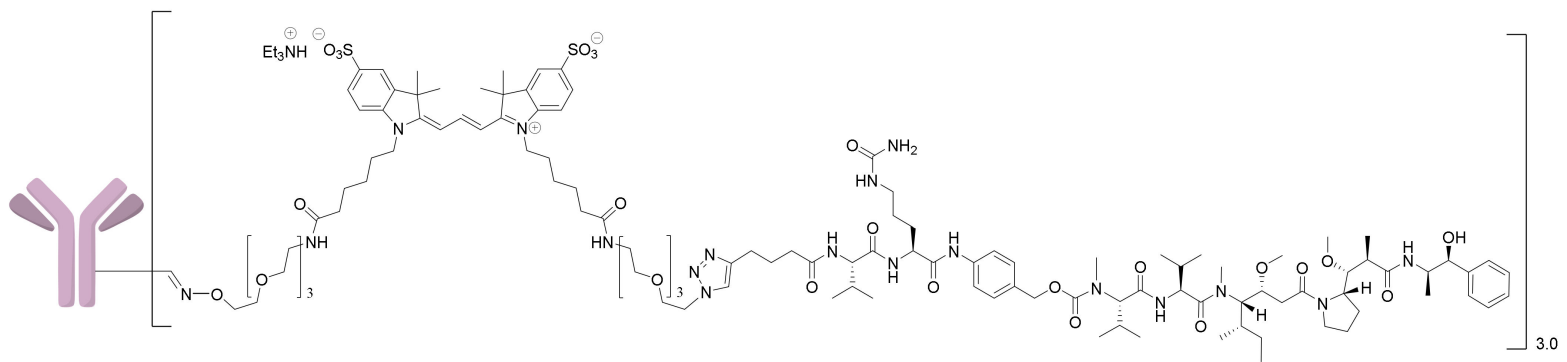
The structure of conjugate **32**.

The 6H8 antibody (1.09 mg, 7.25 nmol) was modified with reagent **2** according to modified general procedure A: after oxidation, the antibody was buffer-exchanged to a 20 mM acetate buffer (pH 5.0, 150 mM NaCl) and treated with reagent **31** (38.1 nm, 5.3 equiv.) A total of 0.64 mg (4.3 nmol, 59%) of conjugate **33** (Figure 14) was obtained with an average degree of labeling of 3.0.

#### 3.3.2. General Procedure B

In a 1.5 mL polypropylene microcentrifuge tube, a solution of CuSO_4_ and THPTA in PBS (10.5 µL, 1.1 mM CuSO_4_, 5.5 mM THPTA), a solution of the CuAAC component (alkyne or azide) in DMSO (14.5 μL, reagent **10**—0.39 mM, reagent **15**—1.15 mM), and an aqueous sodium ascorbate solution (6 μL, 100 mM) were mixed. The reaction mixture was agitated on an orbital shaker for 15 min, then 200 μL of antibody solution in PBS were added, and the reaction mixture was then agitated for 1 h. The conjugate was purified on a Biogel P100 column equilibrated in PBS (pH 7.4); the target fraction was 500 μL. The yield and degree of labeling of the conjugate were determined spectrophotometrically.

##### Conjugate **18**

**Figure 15:**
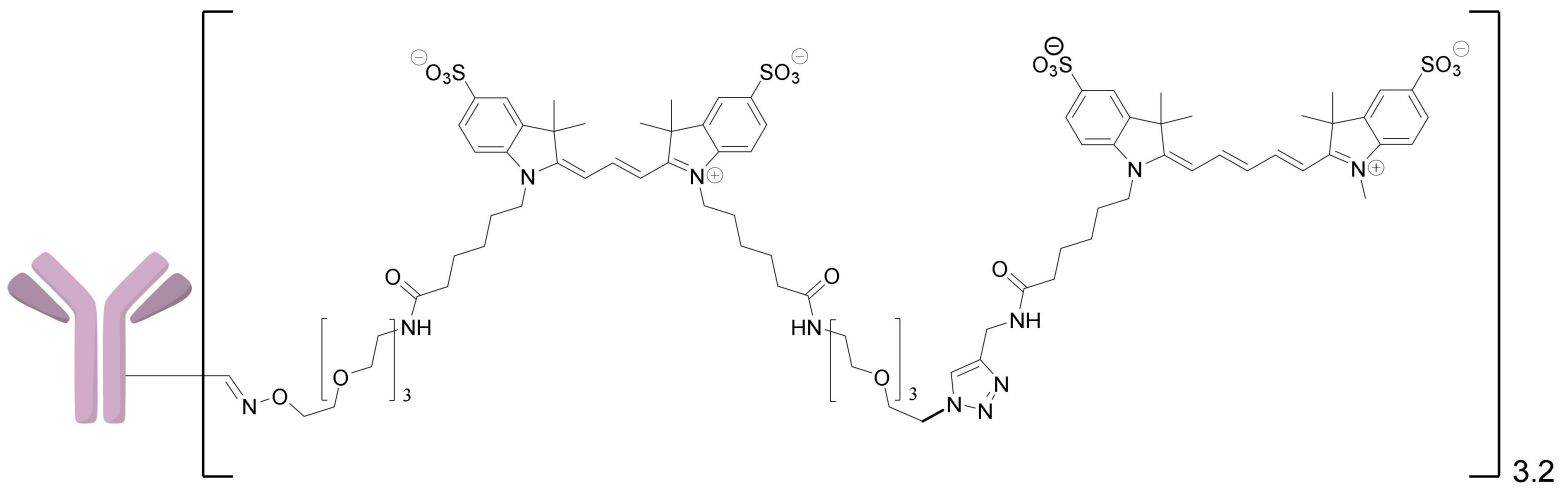
The structure of conjugate **18**.

Conjugate **5** (62 μg, 0.41 nmol) with a degree of labeling of 3.2 was modified with reagent **17** according to general procedure B; 23 μg (0.15 nmol, 37%) of conjugate **24** (Figure 15) were obtained (quantitative yield of CuAAC modification).

##### Conjugate **11**

**Figure 16:**
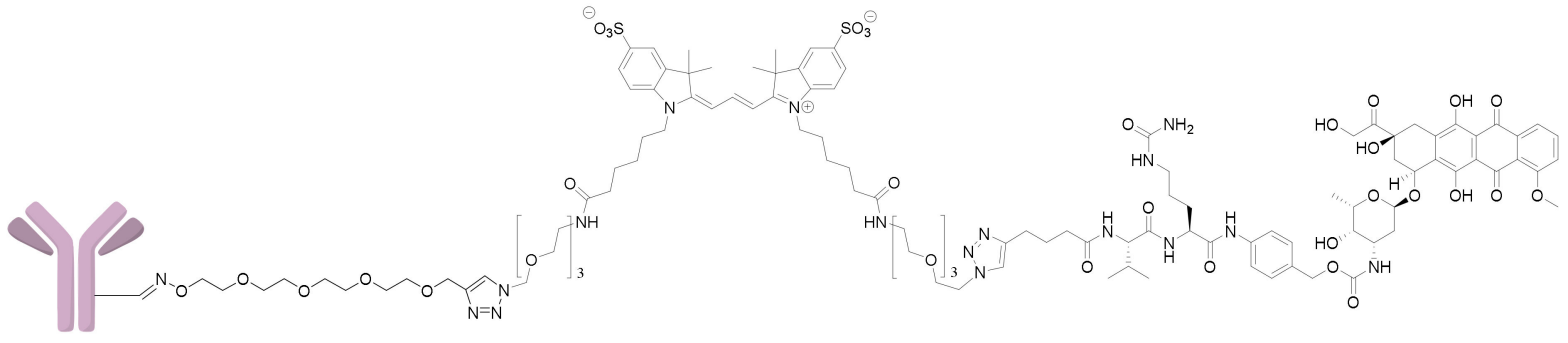
The structure of conjugate **11**.

Conjugate **9** (77 μg, 0.51 nmol) was modified with reagent **10** according to general procedure B; 22 μg (0.15 nmol, 29%) of conjugate **11** (Figure 16) were obtained, the degree of labeling measured by sCy3 absorbance was 1.0.

##### Conjugate **14**

**Figure 17:**
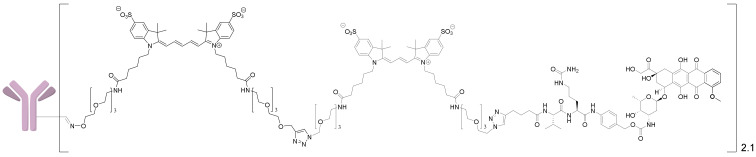
The structure of conjugate **14**.

Conjugate **13** (20 μg, 0.13 nmol) with an average degree of labeling of 2.5 was modified with reagent **10** according to general procedure B; 9.1 μg (0.059 nmol, 46%) of conjugate **14** (Figure 17) were obtained, the degree of modification was 84%.

### 3.4. ELISA Assay

The analysis was performed as described in [12]. A high protein-binding capacity 96-well ELISA plate was coated with recombinant PRAME protein at a concentration of 100 ng in 100 μL of PBS per well. The wells were triple-washed with PBST, and the 6H8 antibody and its derivatives (100 μL per well in PBS with 1% BSA) were added in duplicate at different concentrations. Following incubation on an orbital shaker for 1 h at 23°C and three PBST washes, anti-mouse Fc-specific HRP-labeled antibodies (100 μL per well in PBS with 1% BSA) were added to the wells. After incubation on an orbital shaker for 1 h at 23 °C and triple-washing with PBST, a solution of OPD (100 μL per well) was added. The reaction was stopped by adding 10% sulfuric acid, and optical density (OD) was measured at 490 nm on a Packard SpectraCount BS10000 (PerkinElmer, Waltham, MA, USA) microplate reader.

### 3.5. MTT Assay

Cell viability was analyzed by the MTT assay as described previously [59]. Briefly, cells were cultured in 96-well tissue culture plates (3 × 10^3^ cells/plate) with serial dilutions of ADCs **14** and **31**, the 6H8 antibody, doxorubicin, MMAE, and conjugates **10** and **30** under standard conditions for 72 h. After that, a solution of MTT was added, and incubation was performed for 2 h, after which the medium was discarded and the formazan precipitate was dissolved in DMSO. Optical density (OD) was measured at 540 nm, and cell viability was calculated as:

(OD_treated cells_ − OD_blank_)/(OD_control cells_ − OD_blank_) × 100%

## 4. Conclusions

In summary, we developed a straightforward procedure for the assembly of site-specific ADCs from a periodate-oxidized PRAME antibody and cytotoxic payloads using in situ oxime ligation optionally followed by CuAAC click conjugation, with precise dye-mediated stoichiometry control at each step. The approach seems to be general, suitable for any natively glycosylated monoclonal IgG antibody and any payload with an amino group. The only apparent limitation of the methodology is the dependence of the antibody glycan composition on the expression system, which could lead to variable DAR values. Using the developed methodology, we synthesized the first anti-PRAME antibody–drug conjugate, which demonstrated specific activity in vitro. Further prospects of this approach are: branched linkers; other payloads, including dual ones; other biorthogonal click reactions in the second step, e.g., SPAAC or IEDDA; and variations in the tumor targets of monoclonal IgGs. These studies are in progress, and their results will be reported in due course.

## Data Availability

Not applicable.

## Supplementary Materials

The following supporting information can be downloaded at: https://www.mdpi.com/article/10.3390/ijms24065134/s1.
