# Supplementary material for: Aminooxy Click Modification of a Periodate-Oxidized Immunoglobulin G: A General Approach to Antibody–Drug Conjugates with Dye-Mediated Expeditious Stoichiometry Control
Source: Int J Mol Sci. 2023 Mar 7;24(6):5134. doi: 10.3390/ijms24065134 (PMC10049567; doi:10.3390/ijms24065134)

Supporting Information

Aminooxy click modification of a periodate-oxidized immunoglobulin G: a general approach to antibody-drug conjugates with dye-mediated expeditious stoichiometry control

Ksenia A. Sapozhnikova [1,†], Evgeny L. Gulyak [1,†], Vladimir A. Brylev [1,2], Vsevolod A. Misyurin [3], Sergey D. Oreshkov [1,4], Anastasiya V. Alexeeva [5], Dmitry Yu. Ryazantsev [1], Maria A. Simonova [1], Ekaterina V. Ryabukhina [1], Galina P. Popova [1], Nataliya A. Tikhonova [5], Natalia A. Lyzhko [5], Alexander E. Barmashov [3], Andrey V. Misyurin [5], Alexey V. Ustinov [1,2], Vera A. Alferova [1] and Vladimir A. Korshun [1,*]

[1] Shemyakin-Ovchinnikov Institute of Bioorganic Chemistry, Miklukho-Maklaya 16/10, 117997 Moscow, Russia;
[2] Lumiprobe RUS Ltd., Kotsyubinskogo 4, 121351 Moscow, Russia;
[3] N.N. Blokhin National Medical Cancer Research Center Ministry of Health of Russia, Kashirskoye sh. 24, 115478 Moscow, Russia;
[4] Department of Chemistry, Lomonosov Moscow State University, Leninskie Gory 1/3, 119991 Moscow, Russia;
[5] GeneTechnology LLC, Profsoyuznaya 104, 117485 Moscow, Russia

Table of Contents

NMR spectra S2
HRMS spectra S20

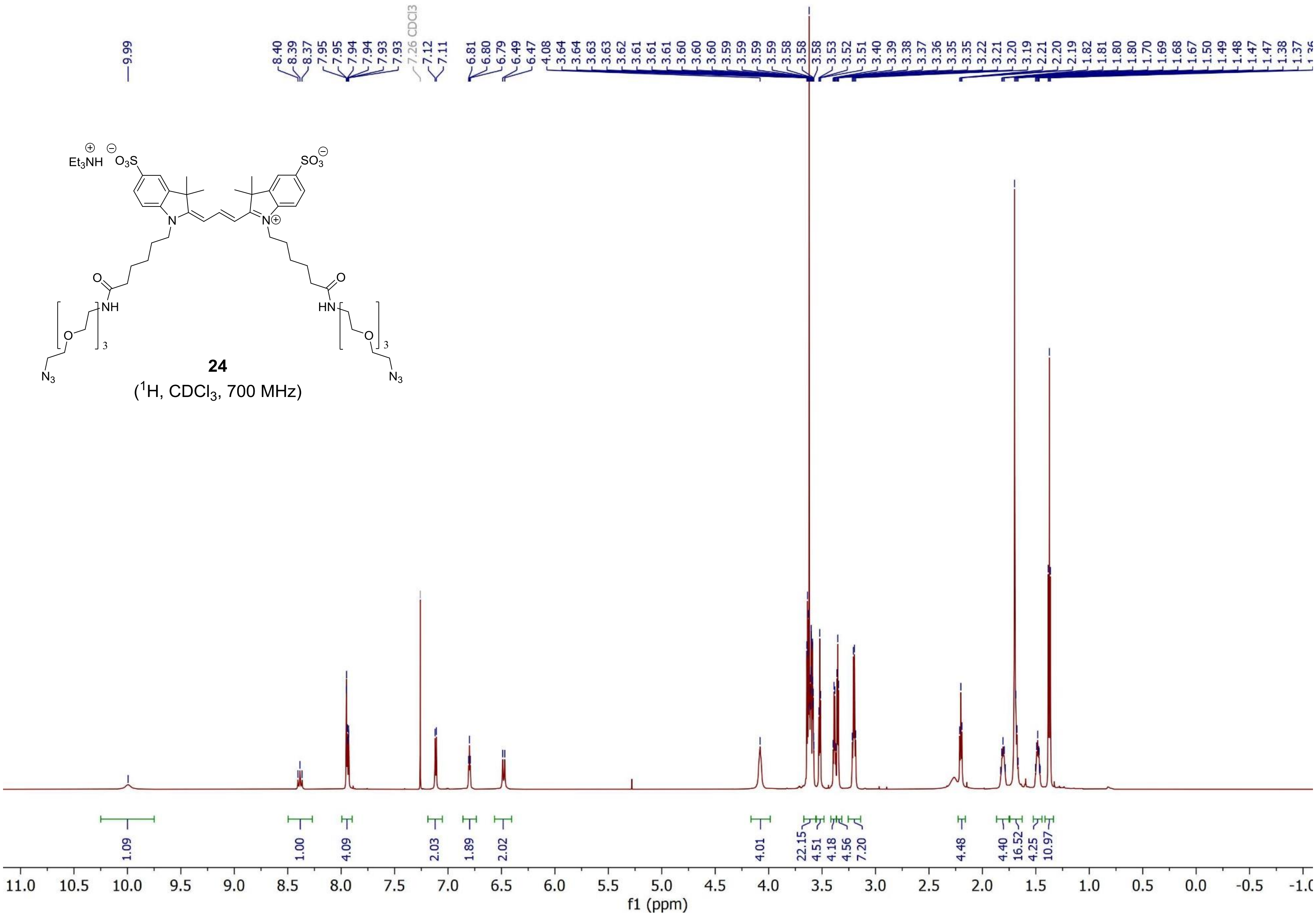
9.99
8.40 8.39 8.37 7.95 7.95 7.94 7.94 7.93 7.93 7.26 CDCl3 7.12 7.11 6.81 6.80 6.79 6.49 6.47 4.08 3.64 3.64 3.63 3.63 3.62 3.61 3.61 3.61 3.60 3.60 3.60 3.59 3.59 3.59 3.59 3.58 3.58 3.58 3.53 3.52 3.51 3.40 3.39 3.38 3.37 3.36 3.35 3.35 3.22 3.21 3.20 3.19 2.21 2.20 2.19 1.82 1.81 1.80 1.80 1.70 1.69 1.68 1.67 1.50 1.49 1.48 1.47 1.38 1.37
Et3NH
O3S
SO3
N
N
O
NH
O
HN
O
O
3
3
N3
N3
24
(1H, CDCl3, 700 MHz)
1.09 1.00 4.09 2.03 1.89 2.02 4.01 22.15 4.51 4.18 4.56 7.20 4.48 4.40 16.52 4.25 10.97
11.0 10.5 10.0 9.5 9.0 8.5 8.0 7.5 7.0 6.5 6.0 5.5 5.0 4.5 4.0 3.5 3.0 2.5 2.0 1.5 1.0 0.5 0.0 -0.5
f1 (ppm)

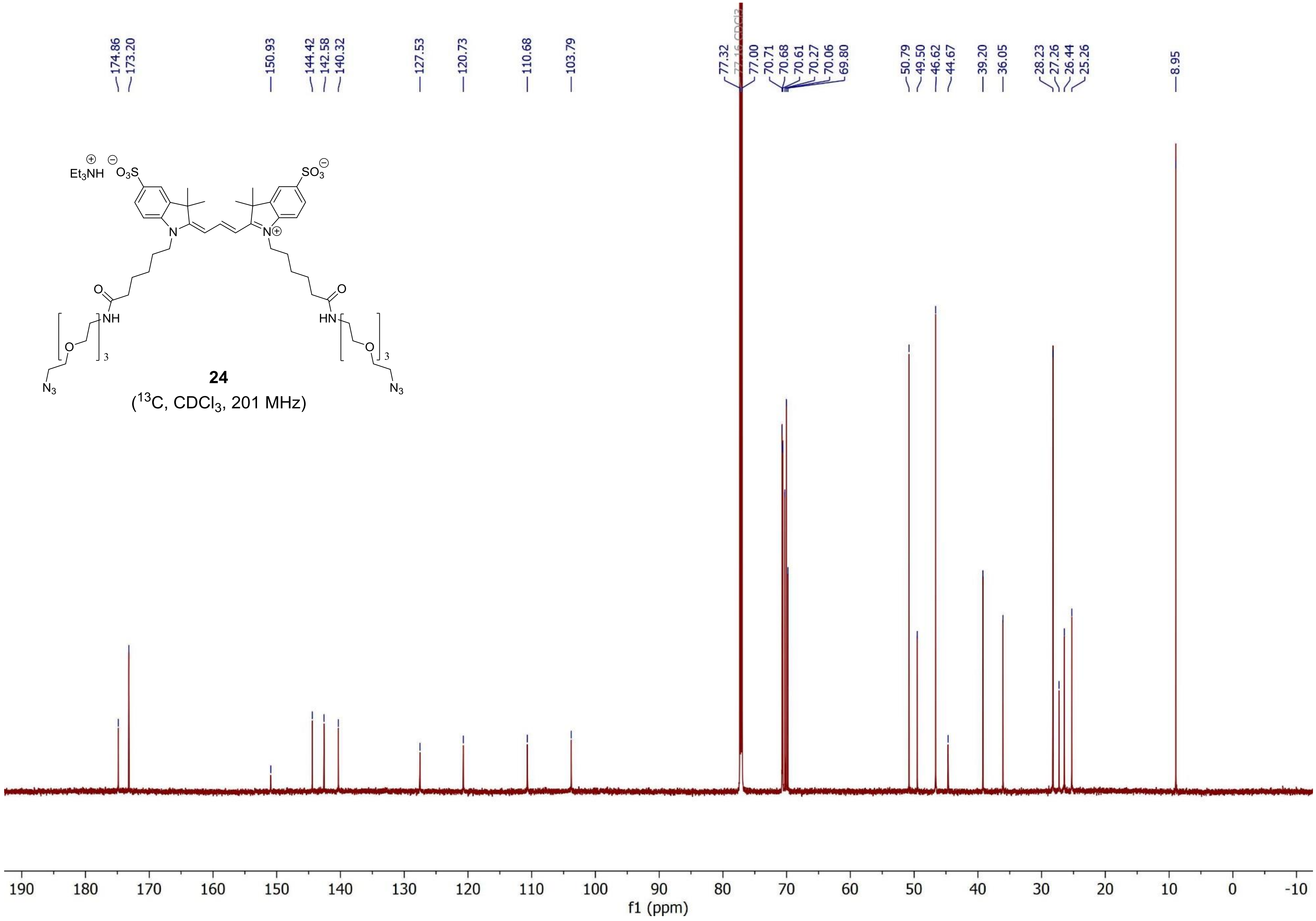

174.86
173.20
150.93
144.42
142.58
140.32
127.53
120.73
110.68
103.79
77.32
77.16 CDCl3
77.00
70.71
70.68
70.61
70.27
70.06
69.80
50.79
49.50
46.62
44.67
39.20
36.05
28.23
27.26
26.44
25.26
8.95
Et3NH
O3S
SO3
N
N
O
NH
HN
O
O
O
3
3
N3
N3
24
(13C, CDCl3, 201 MHz)
190 180 170 160 150 140 130 120 110 100 90 80 70 60 50 40 30 20 10 0 -10
f1 (ppm)

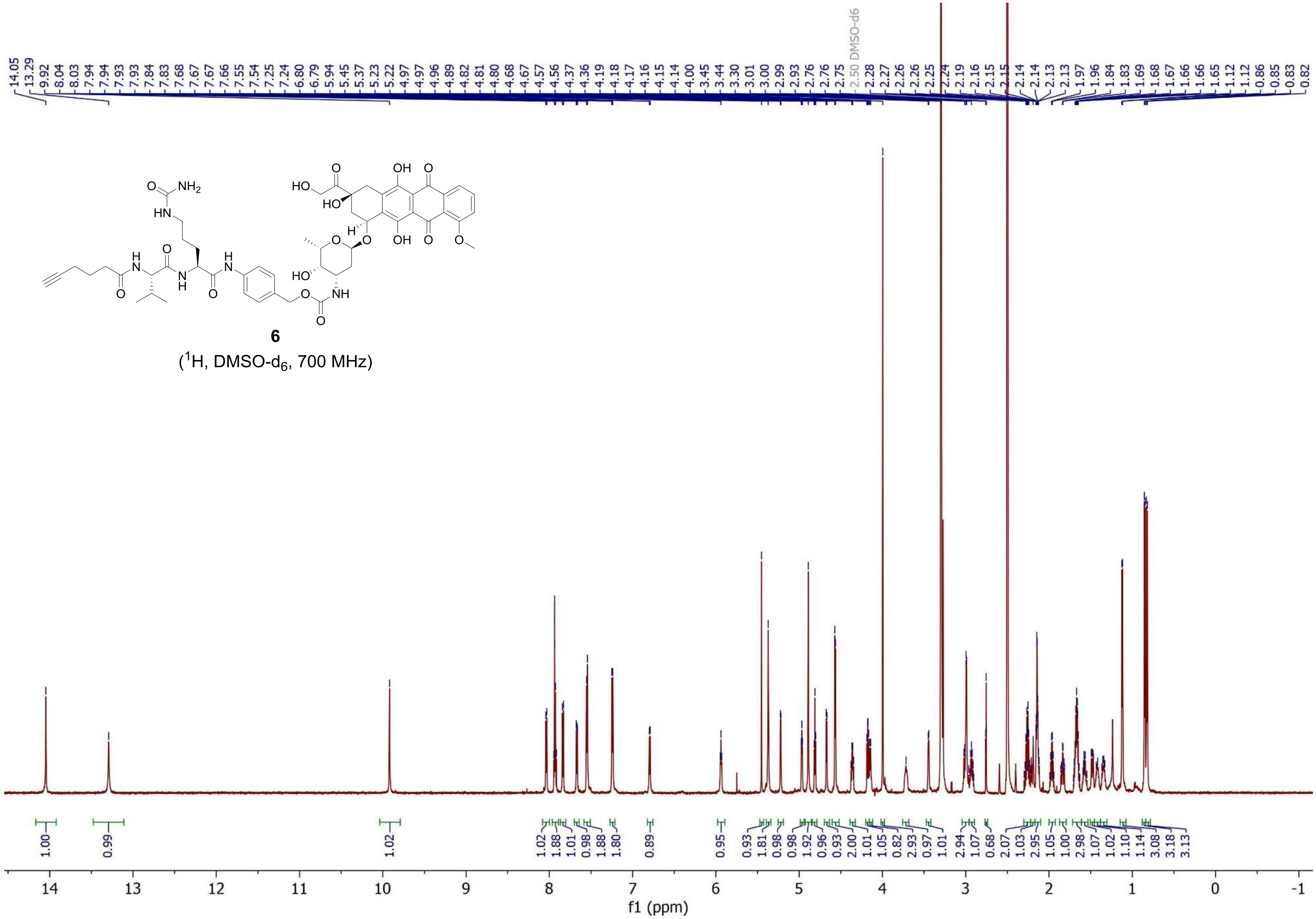
6
(^{1}H, DMSO-d$_6$, 700 MHz)
f1 (ppm)

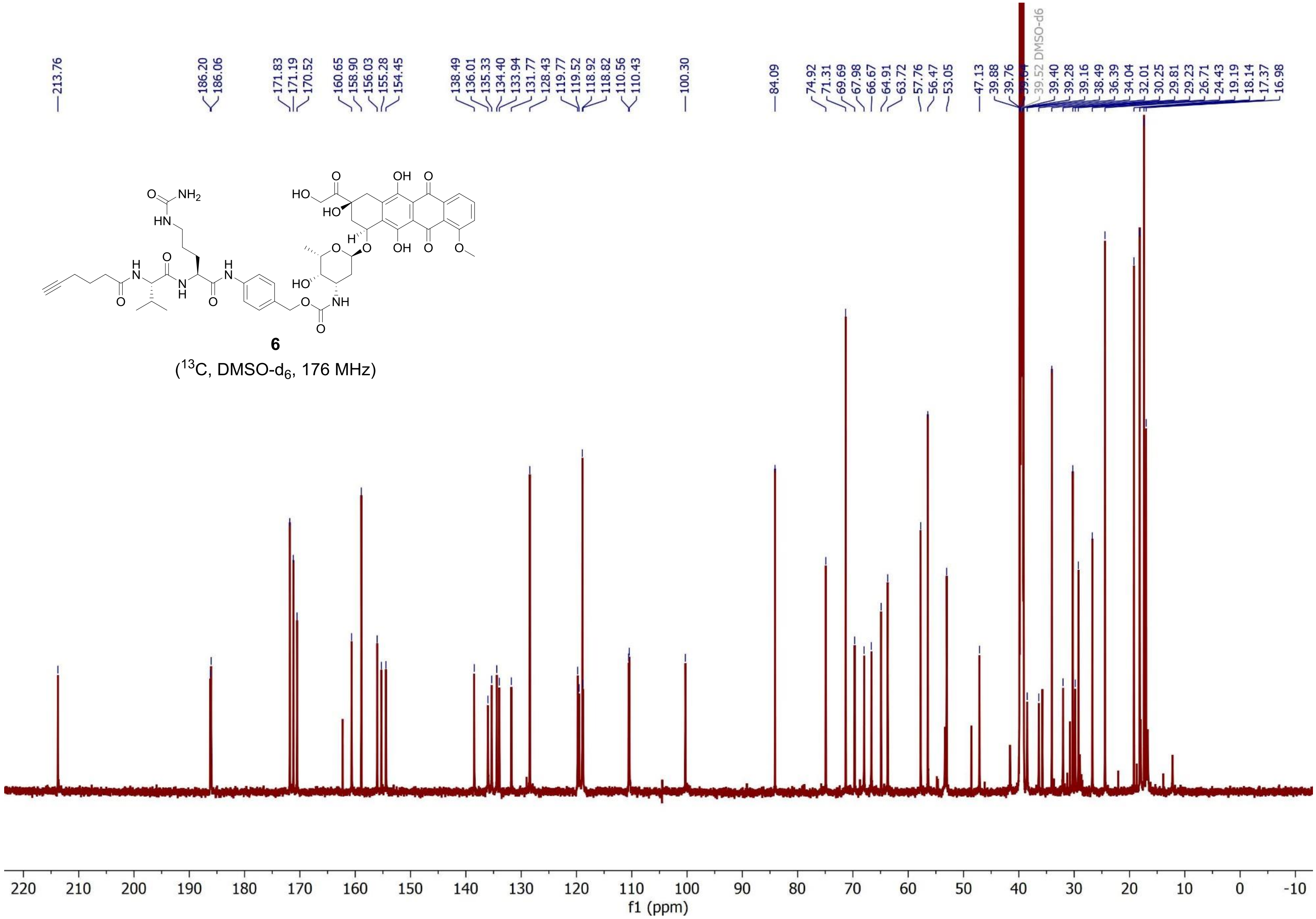

6

(^{13}C, DMSO-d$_6$, 176 MHz)

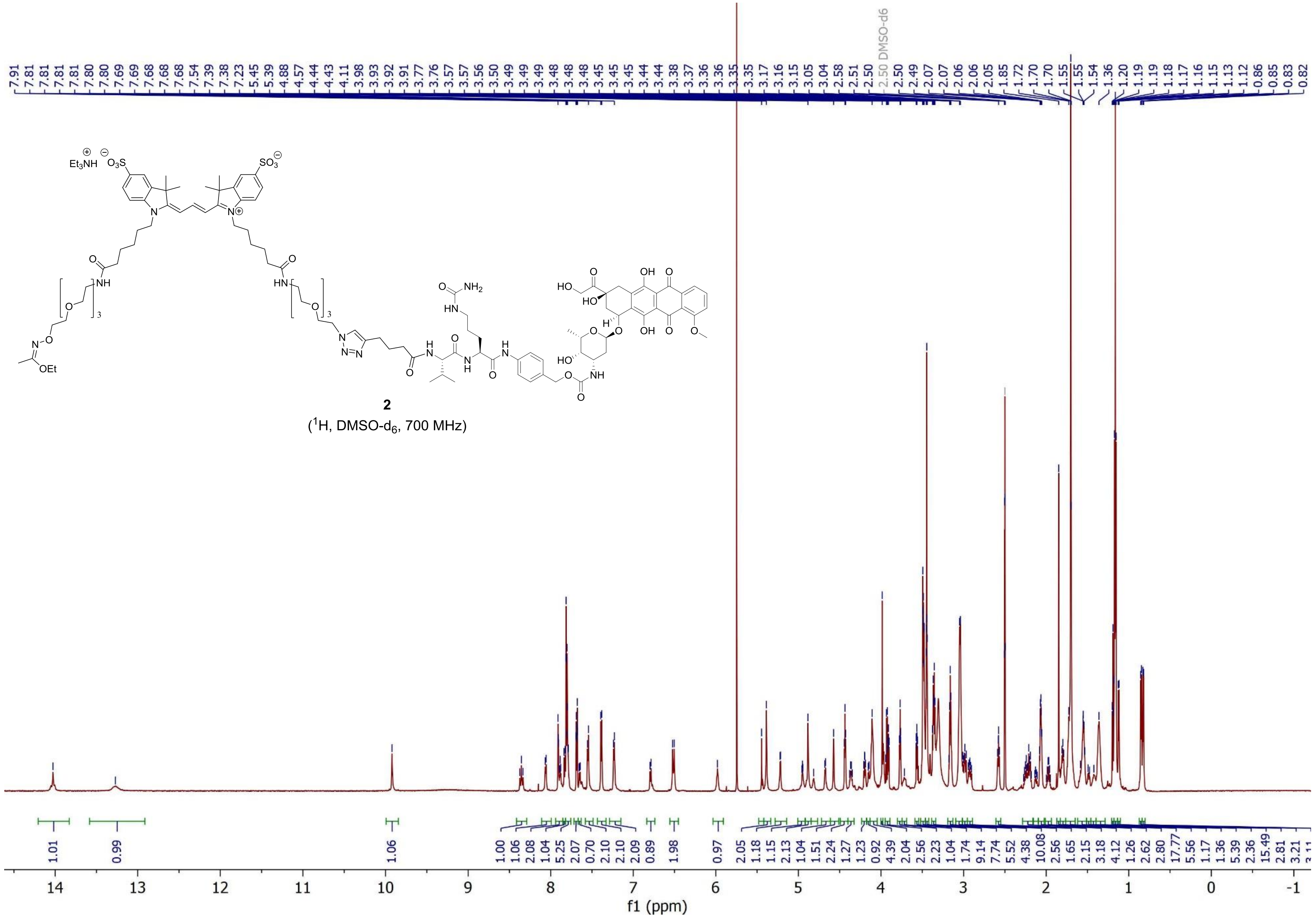

2

(^{1}H, DMSO-d_6, 700 MHz)

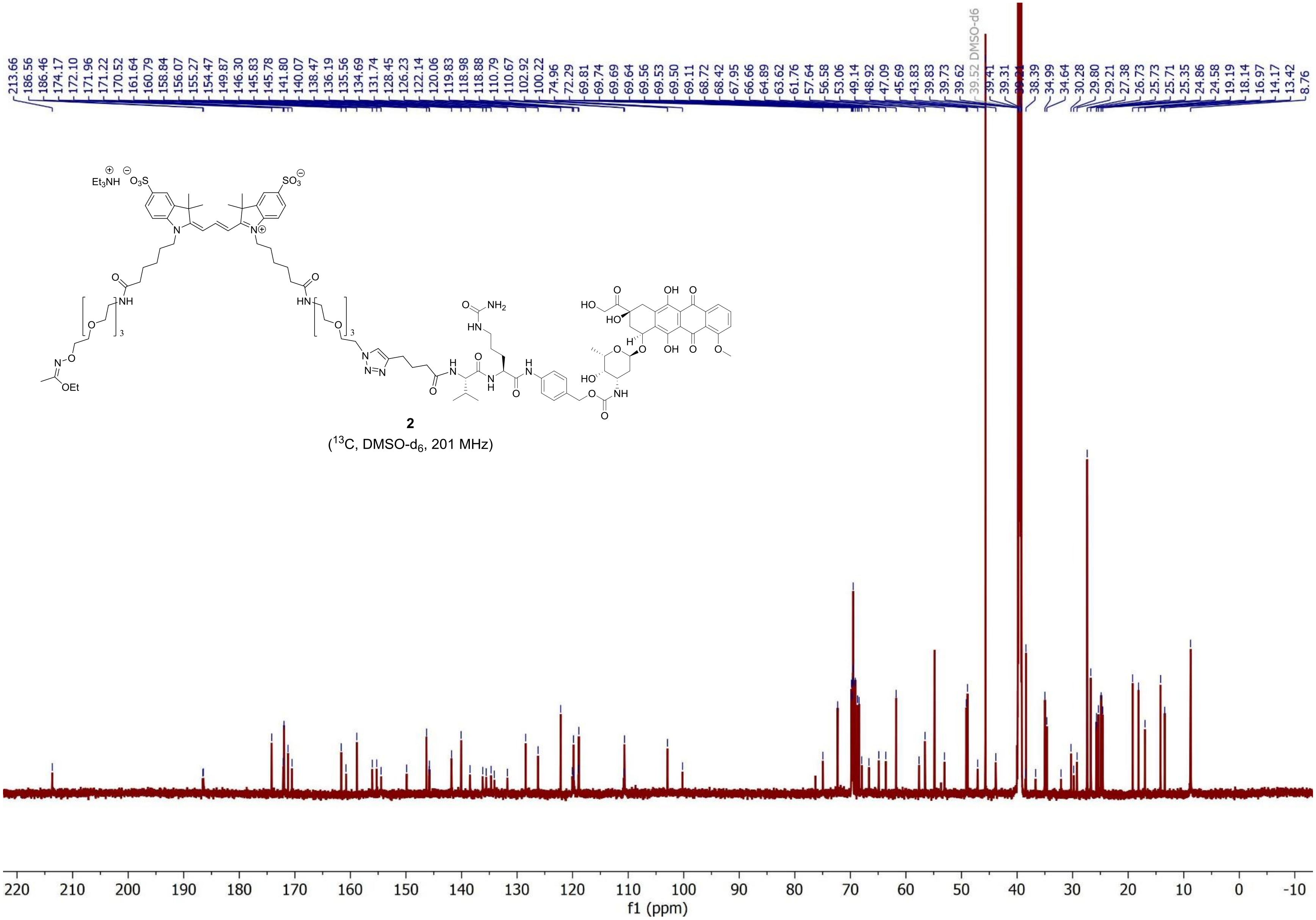

2
(^{13}C, DMSO-d$_6$, 201 MHz)

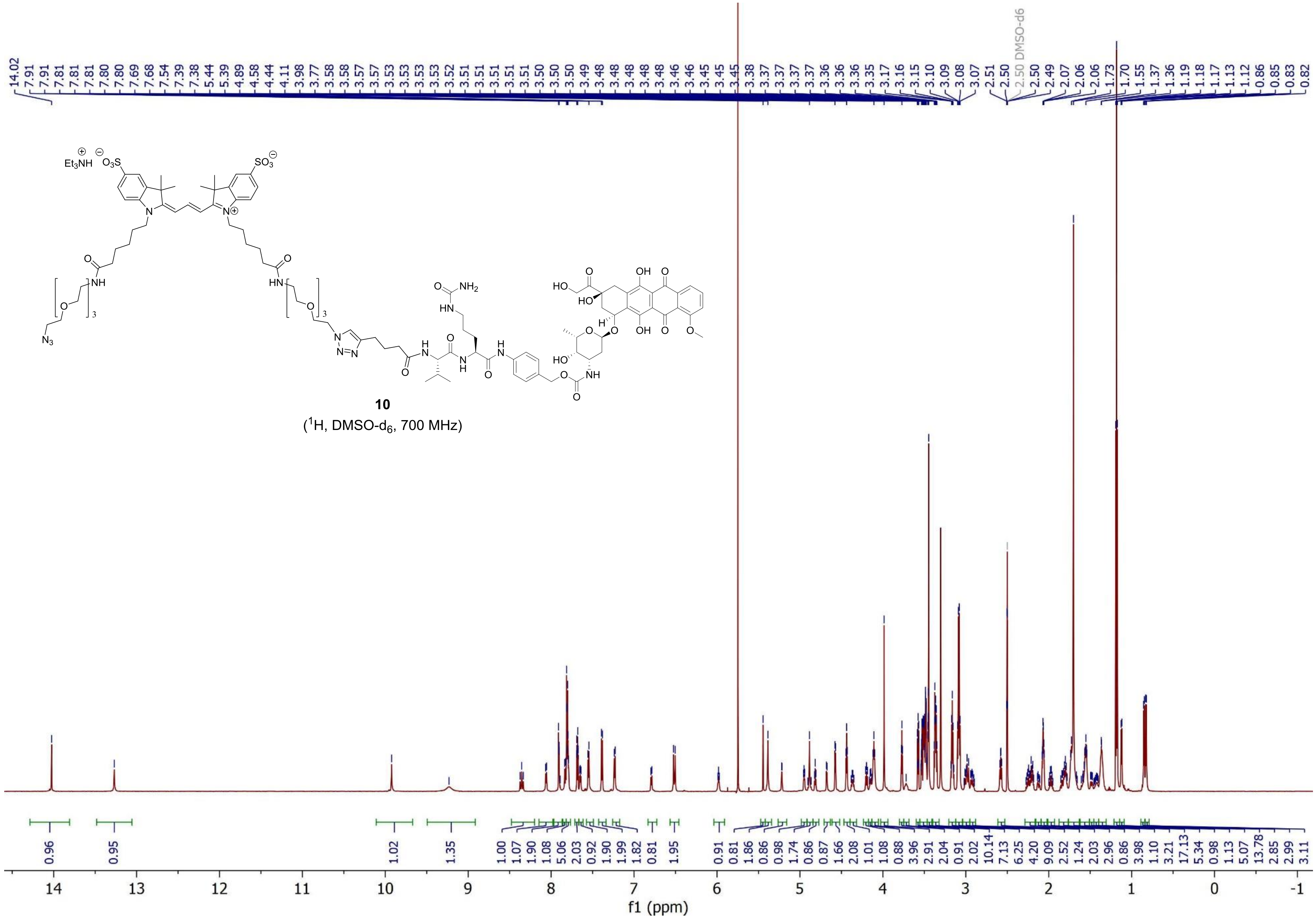

10

(^{1}H, DMSO-d_6, 700 MHz)

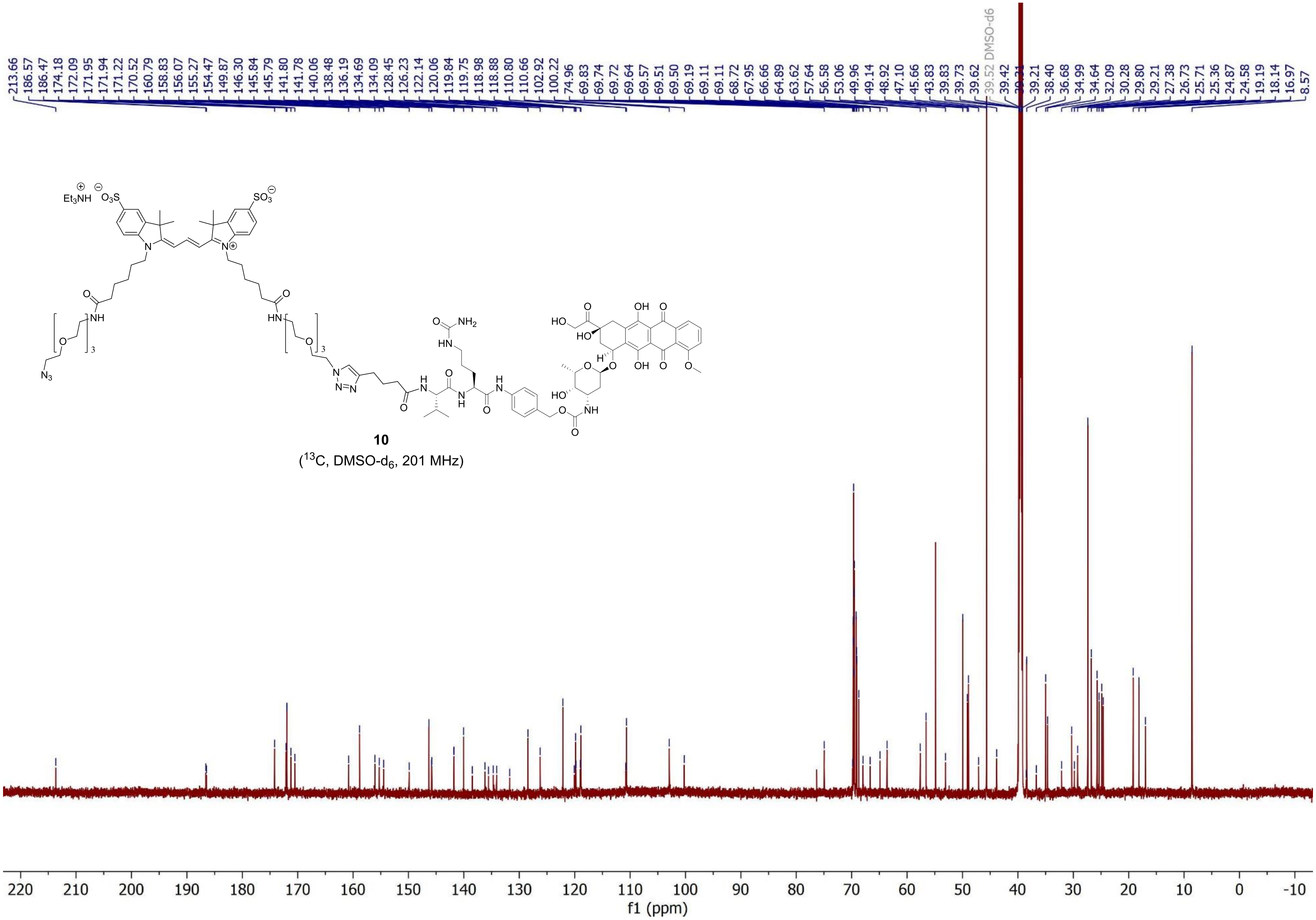

10

(^{13}C, DMSO-d_6, 201 MHz)

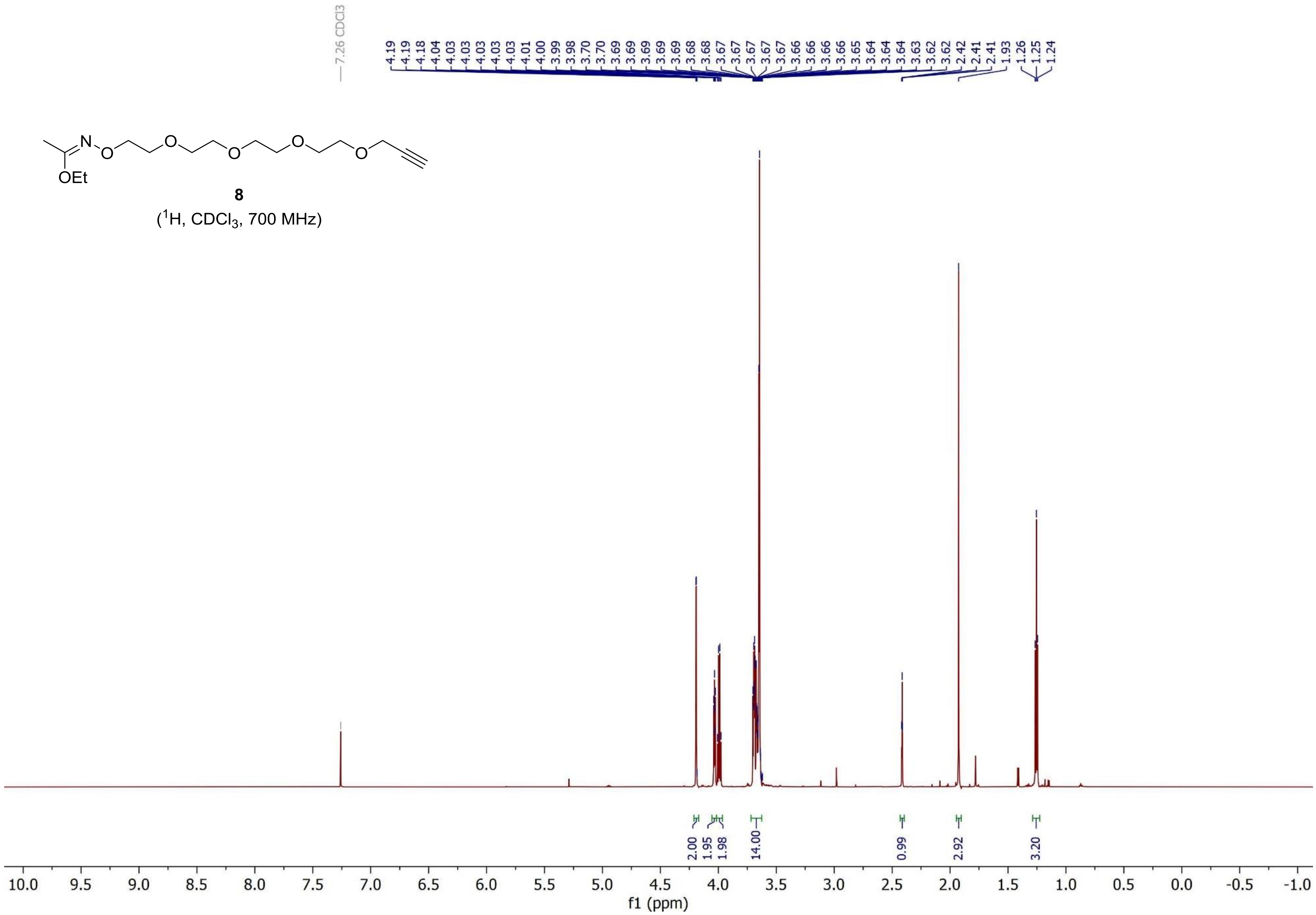
OEt
N
O
8
(¹H, CDCl₃, 700 MHz)
7.26 CDCl3
4.19 4.19 4.18 4.04 4.03 4.03 4.03 4.03 4.01 4.00 3.99 3.98 3.70 3.70 3.69 3.69 3.69 3.69 3.68 3.68 3.67 3.67 3.67 3.67 3.67 3.66 3.66 3.66 3.65 3.64 3.64 3.63 3.62 3.62 2.42 2.41 2.41 1.93 1.26 1.25 1.24
2.00 1.95 1.98 14.00 0.99 2.92 3.20
10.0 9.5 9.0 8.5 8.0 7.5 7.0 6.5 6.0 5.5 5.0 4.5 4.0 3.5 3.0 2.5 2.0 1.5 1.0 0.5 0.0 -0.5 -1.0
f1 (ppm)

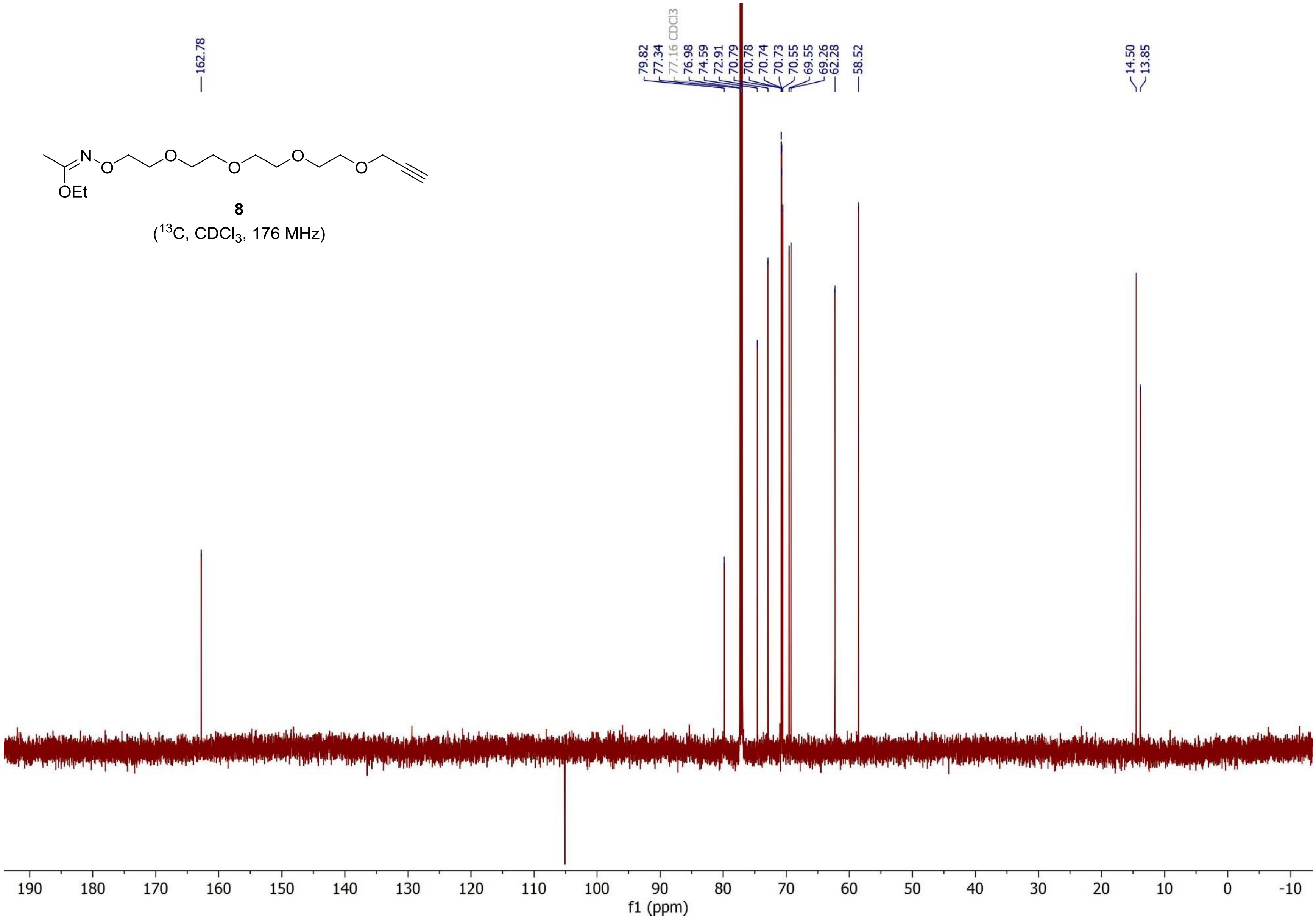

8

(^{13}C, $CDCl_3$, 176 MHz)

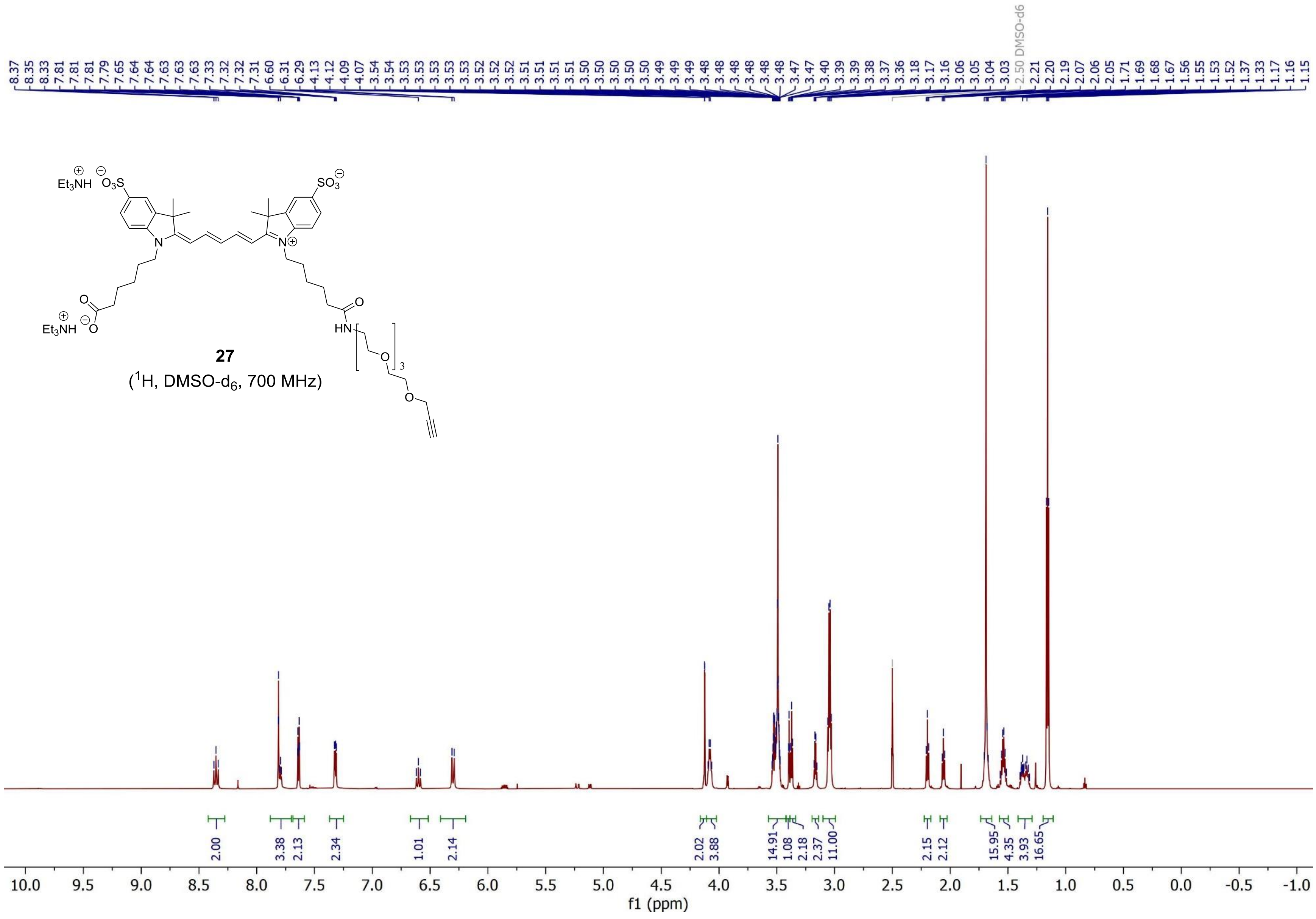

27

(^{1}H, DMSO-d$_6$, 700 MHz)

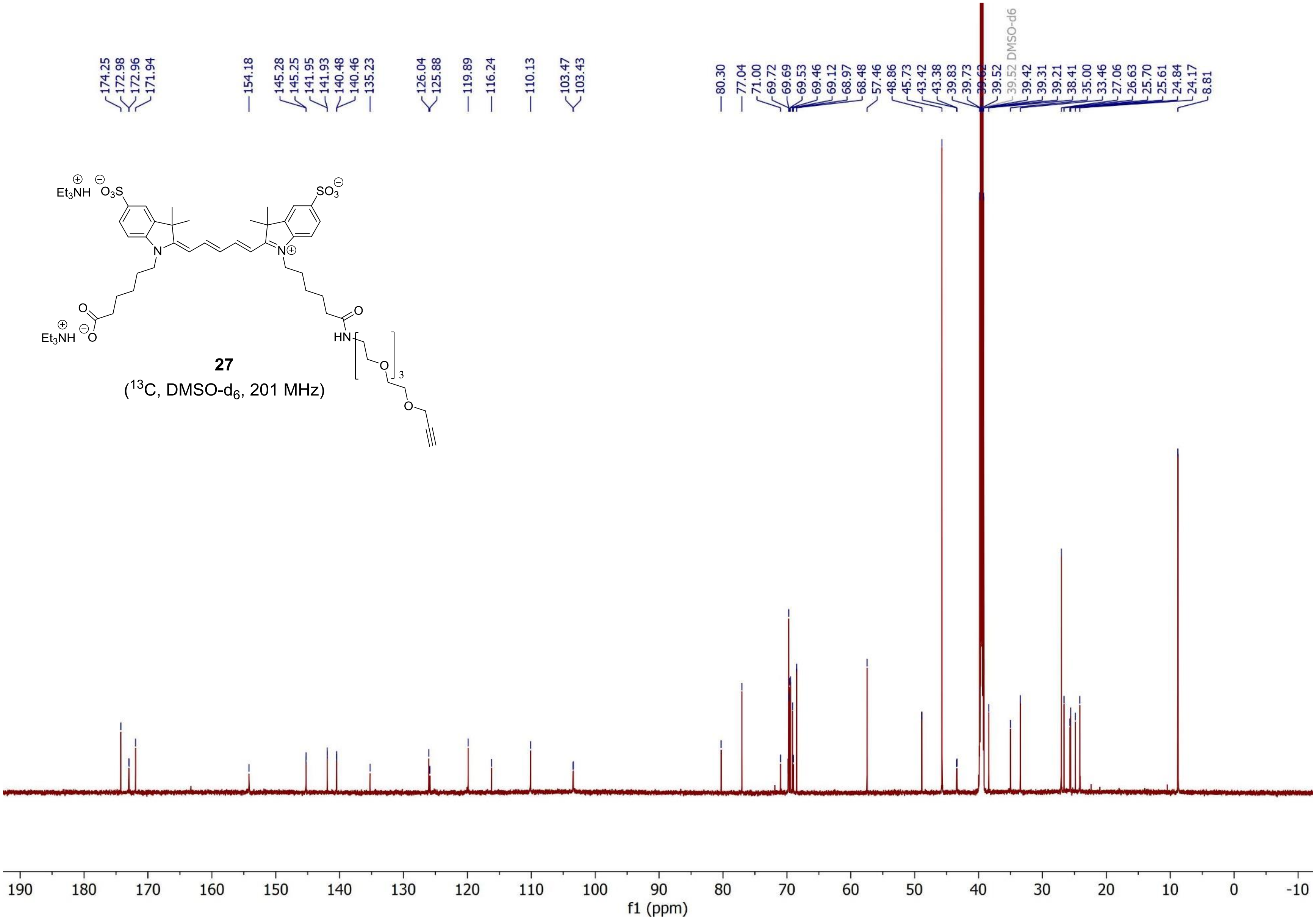

27

(^{13}C, DMSO-d$_6$, 201 MHz)

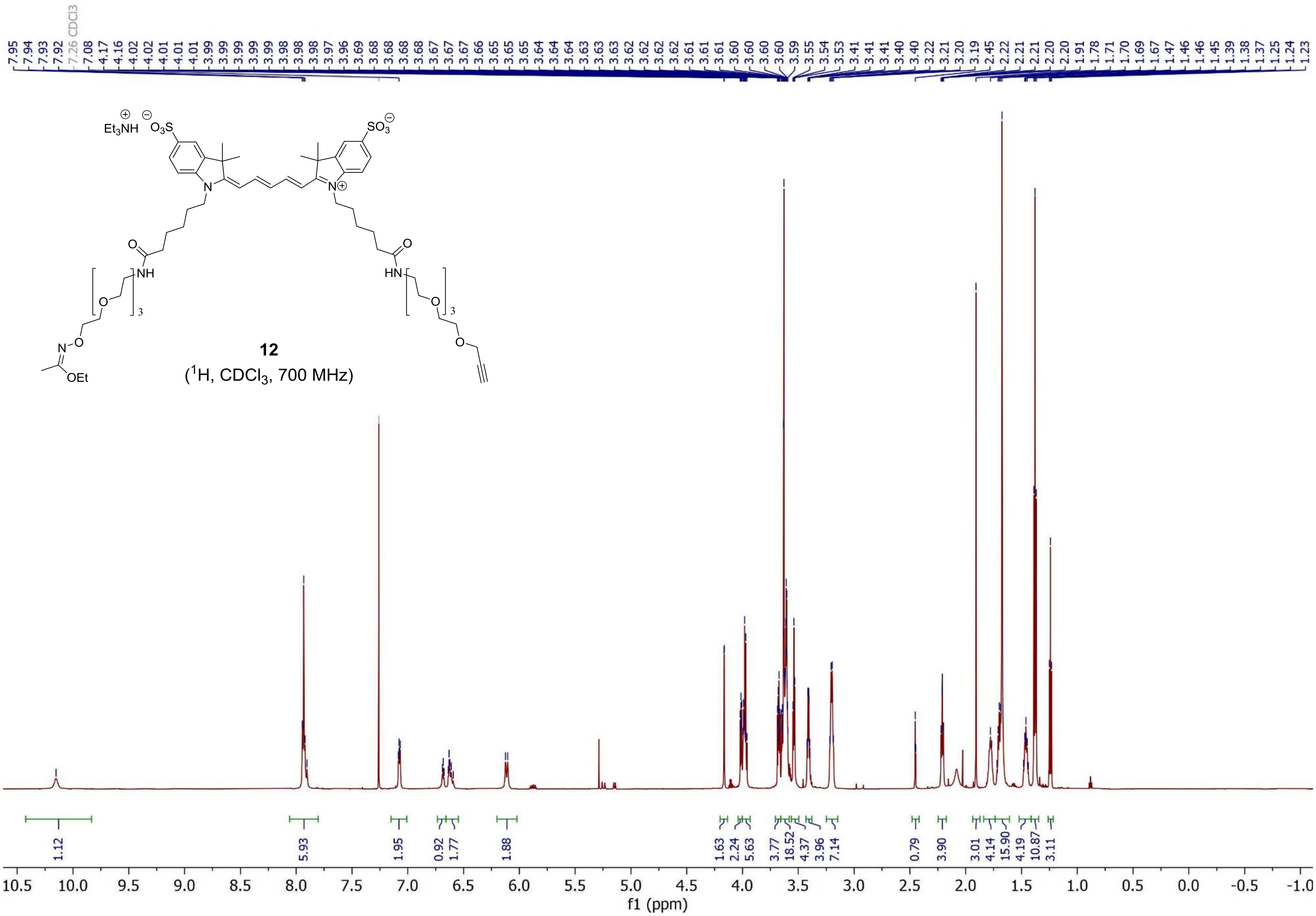
Et3NH O3S
SO3
N
N
O
NH
HN
O
3
3
O
O
N
O
OEt
O
12
(1H, CDCl3, 700 MHz)
f1 (ppm)

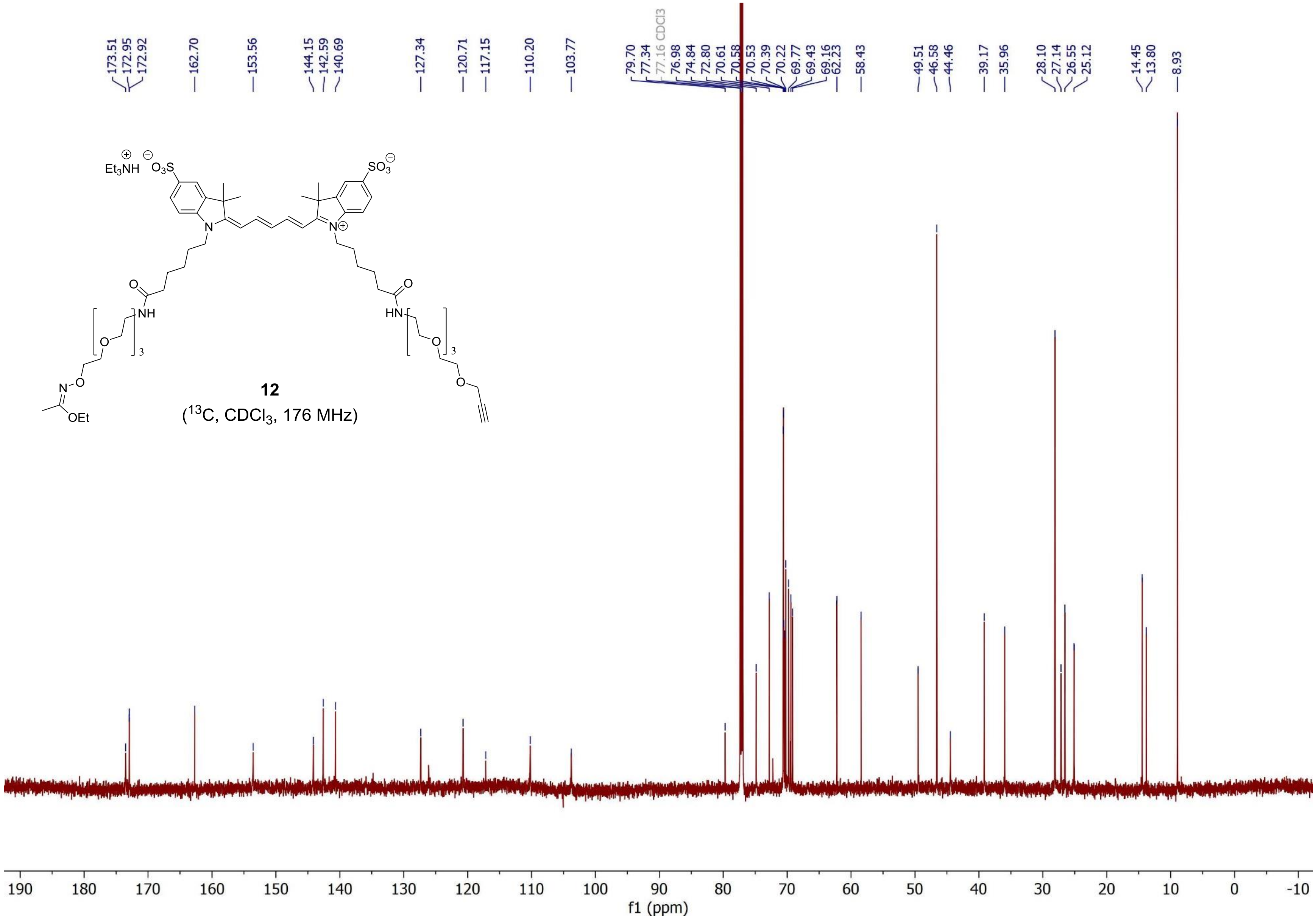
173.51
172.95
172.92
162.70
153.56
144.15
142.59
140.69
127.34
120.71
117.15
110.20
103.77
79.70
77.34
77.16 CDCl3
76.98
74.84
72.80
70.61
70.58
70.53
70.39
70.22
69.77
69.43
69.16
62.23
58.43
49.51
46.58
44.46
39.17
35.96
28.10
27.14
26.55
25.12
14.45
13.80
8.93
Et3NH
O3S
SO3
N
N
O
NH
HN
O
3
3
N
O
OEt
12
(13C, CDCl3, 176 MHz)
f1 (ppm)

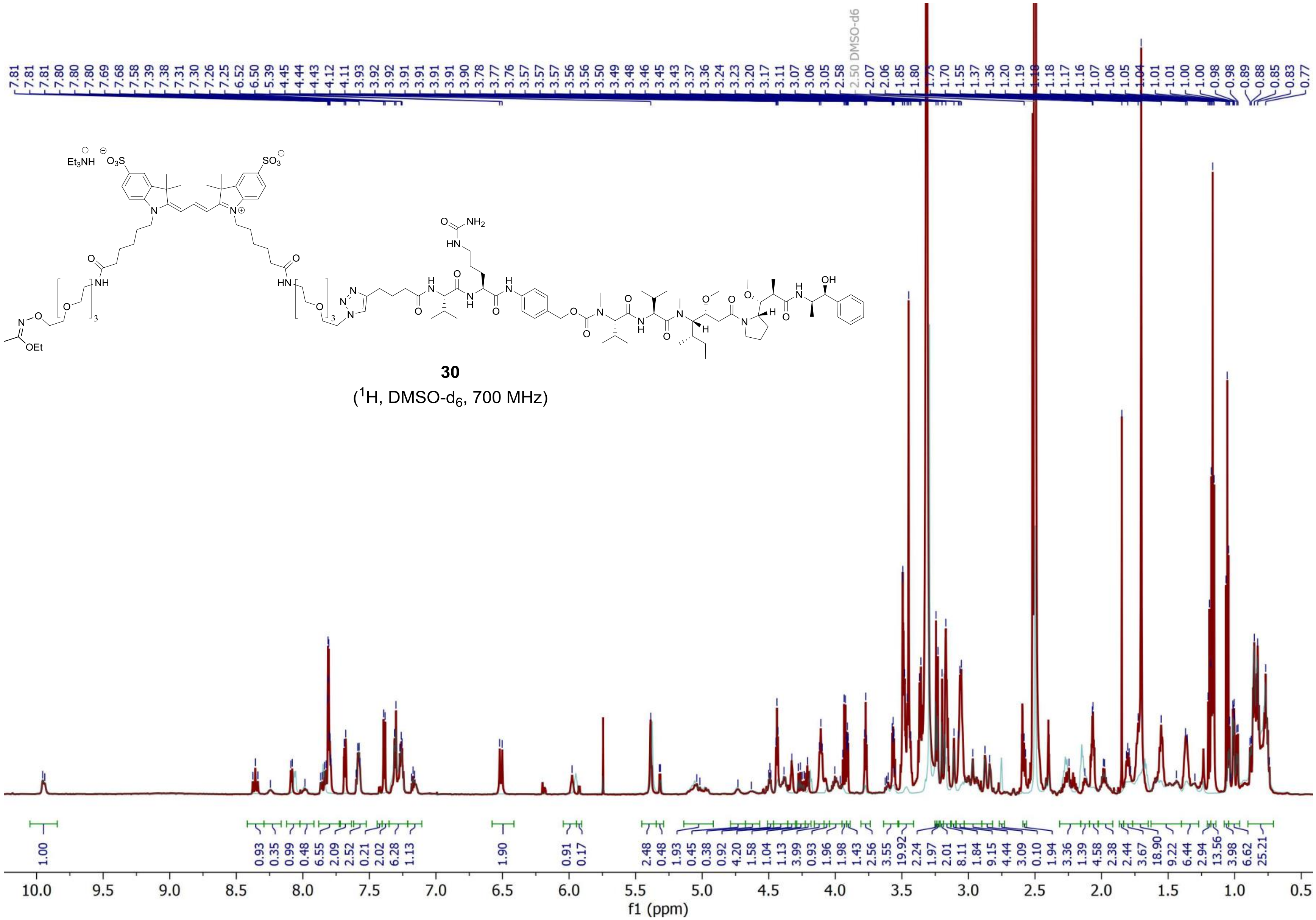

30

(^{1}H, DMSO-d$_6$, 700 MHz)

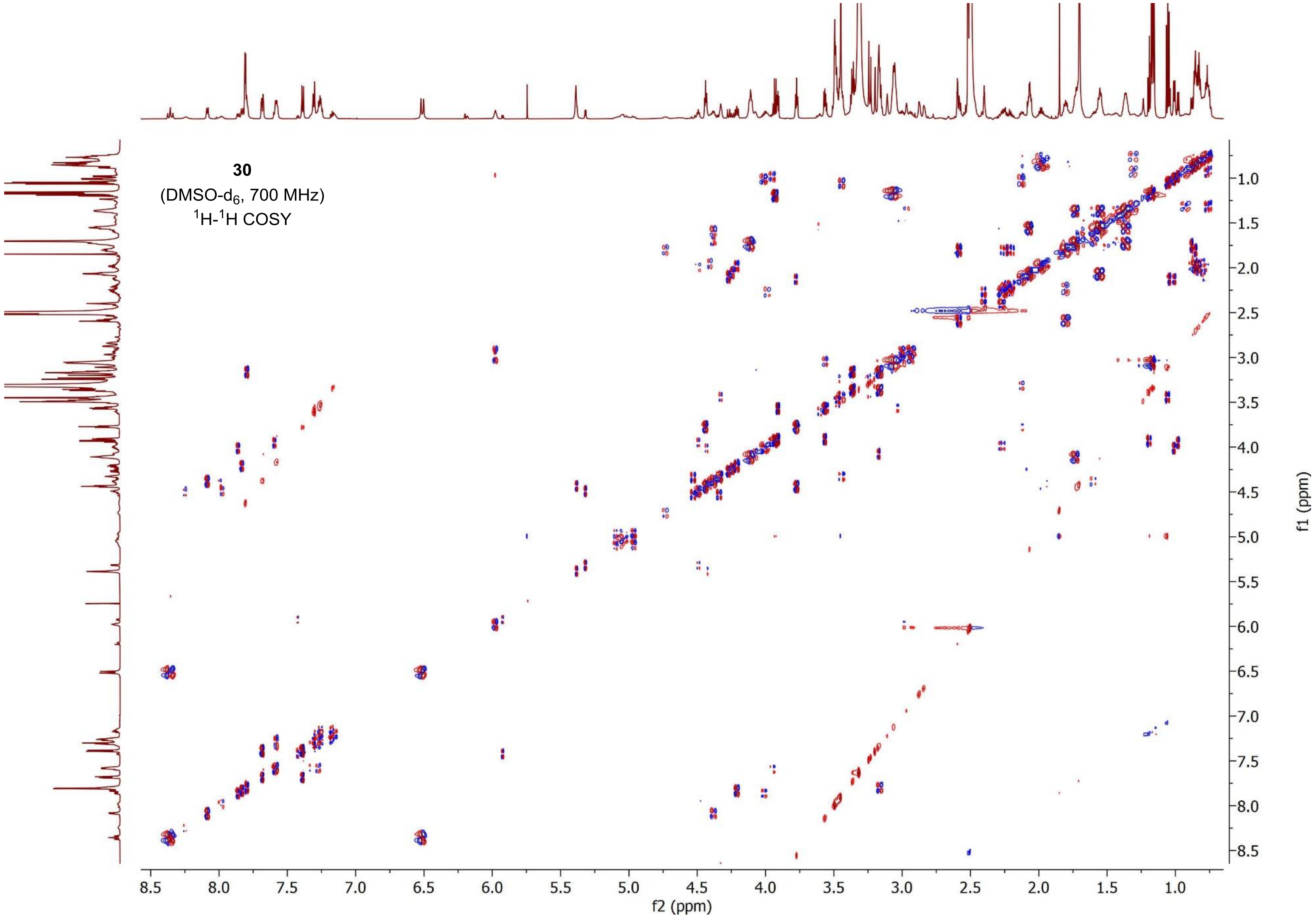
30
(DMSO-d6, 700 MHz)
1H-1H COSY
f2 (ppm)
f1 (ppm)

30
(DMSO-d_6, 700 MHz)
^1H-^{13}C HSQC

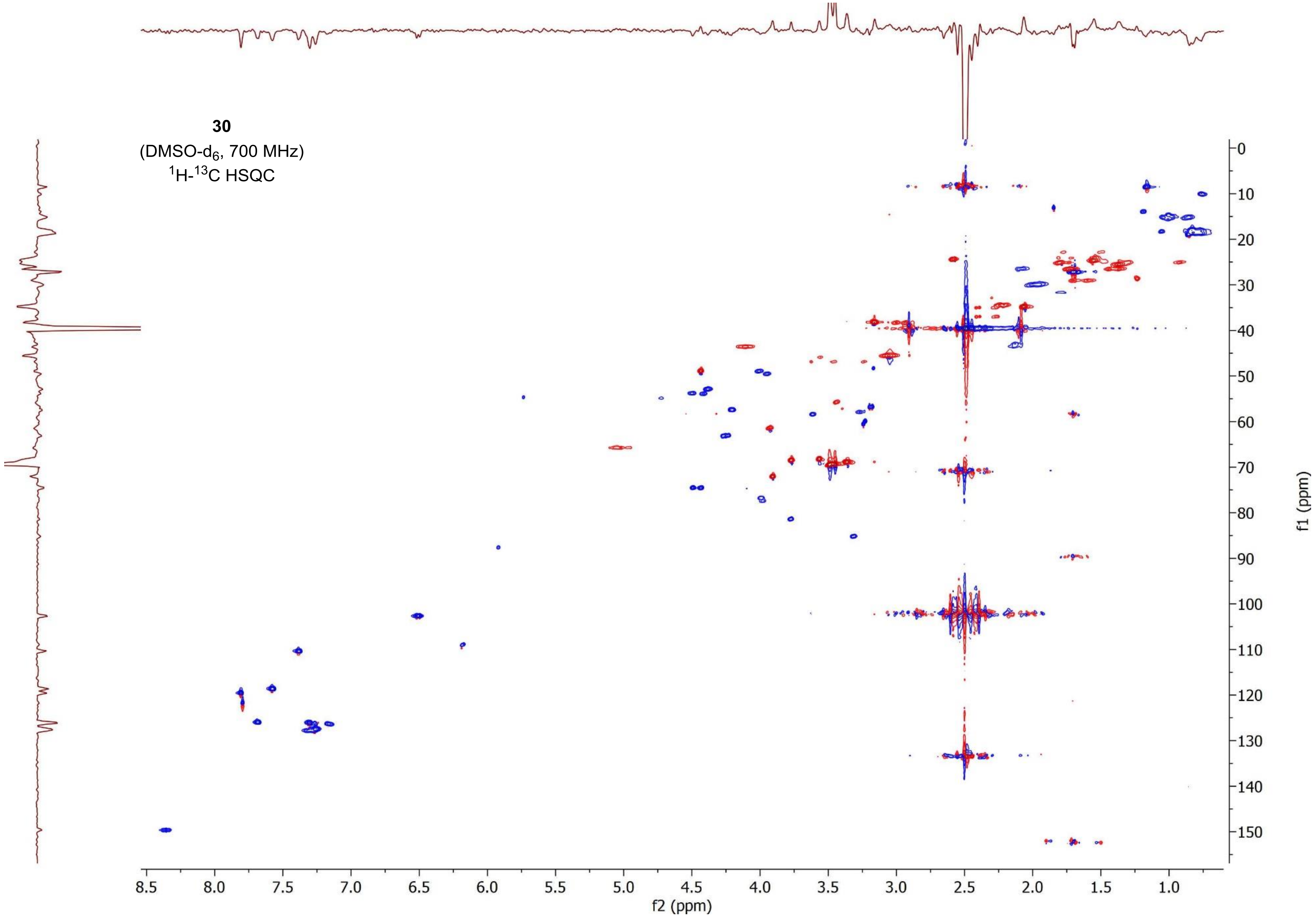

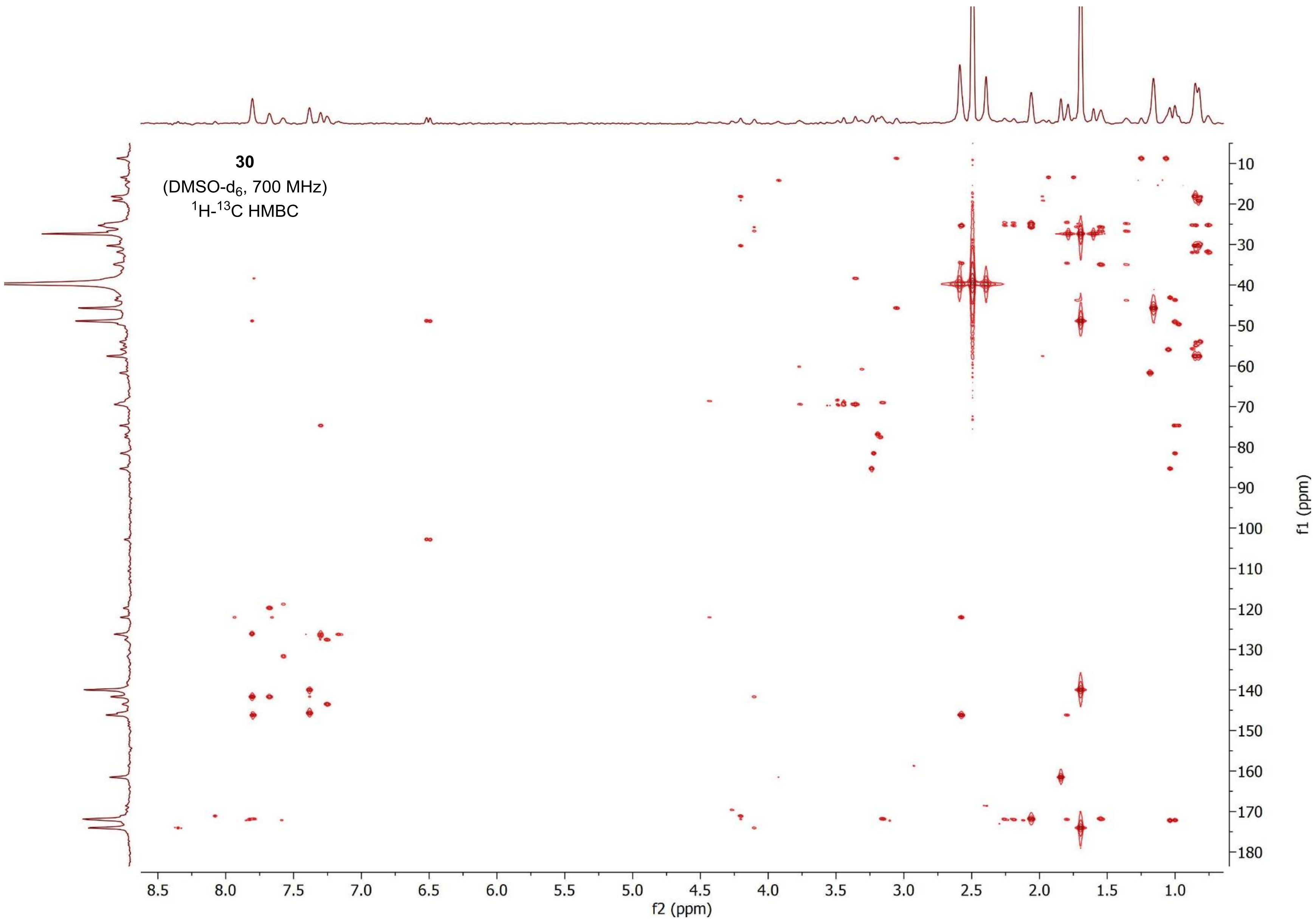
30
(DMSO-d6, 700 MHz)
1H-13C HMBC
f2 (ppm)
f1 (ppm)
8.5
8.0
7.5
7.0
6.5
6.0
5.5
5.0
4.5
4.0
3.5
3.0
2.5
2.0
1.5
1.0
10
20
30
40
50
60
70
80
90
100
110
120
130
140
150
160
170
180

HRMS for diazide 24, negative mode.

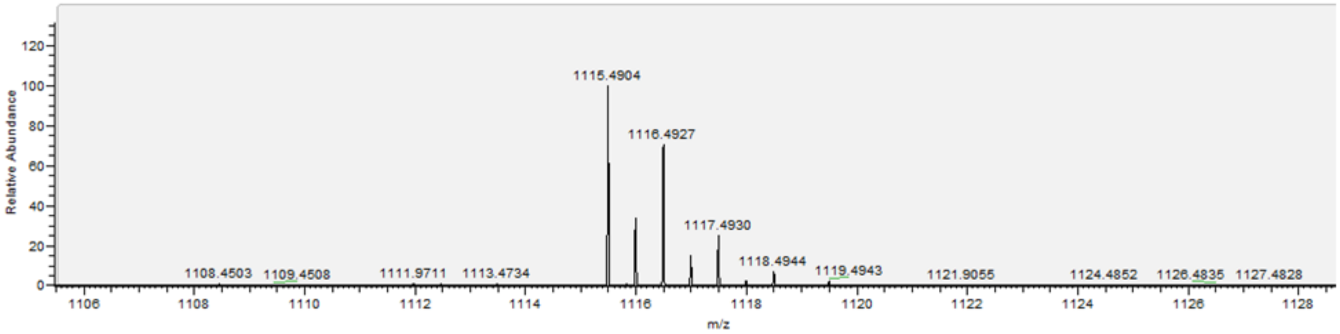

HRMS for conjugate 6, positive mode.

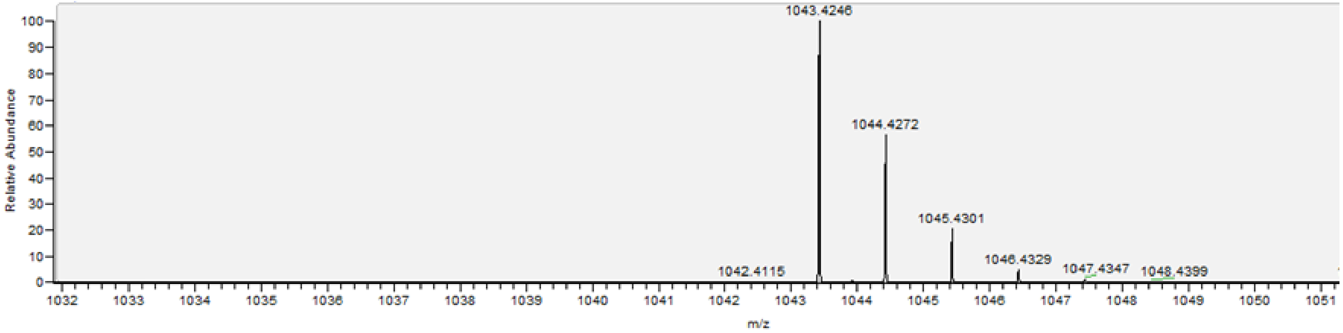

HRMS for conjugate 2, negative mode.

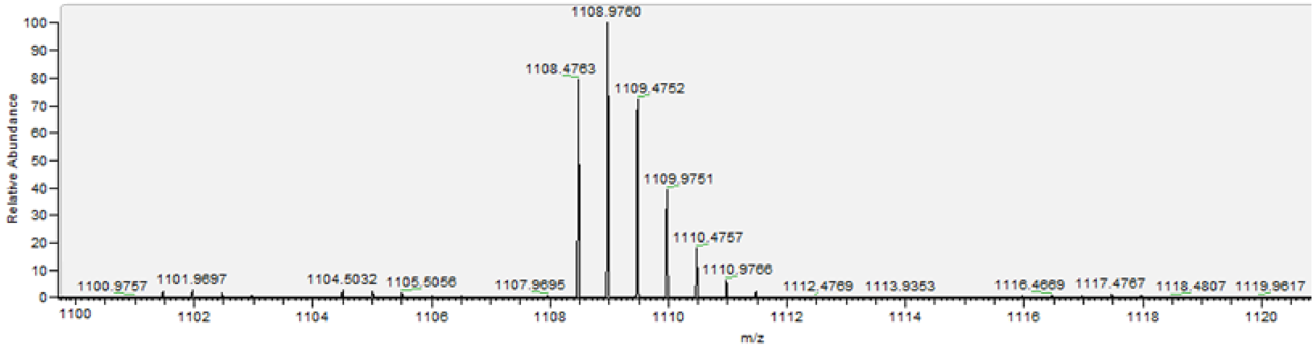

HRMS for conjugate 10, negative mode.

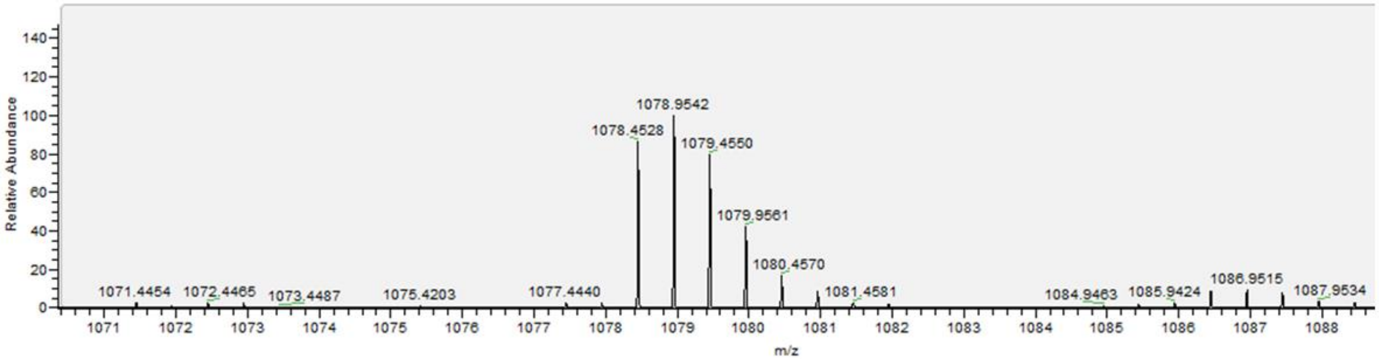

HRMS for imidate 8, positive mode.

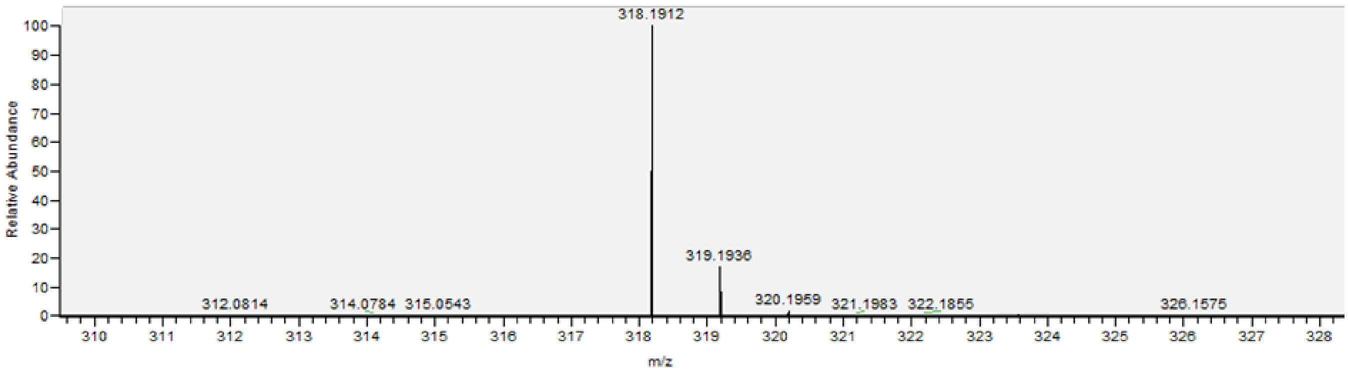

HRMS for alkyne 27, negative mode.

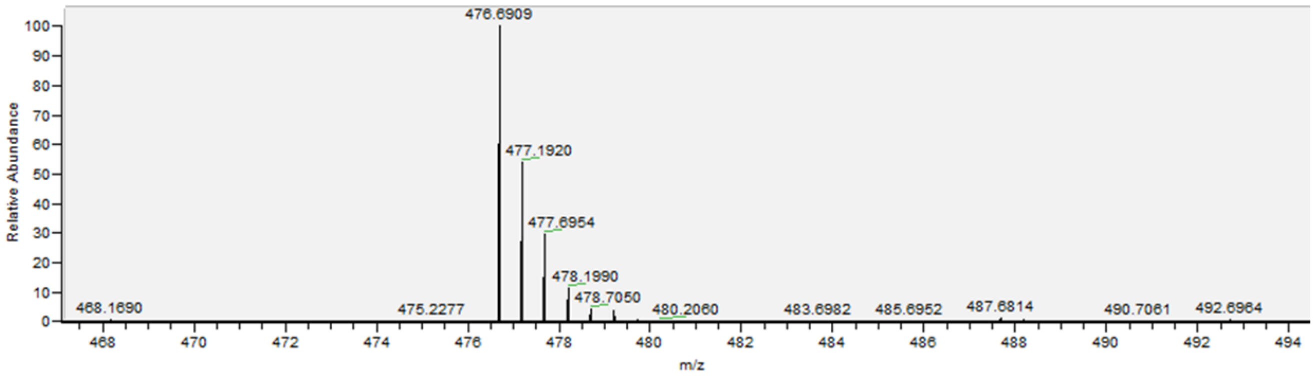

HRMS for imidate 12, negative mode.

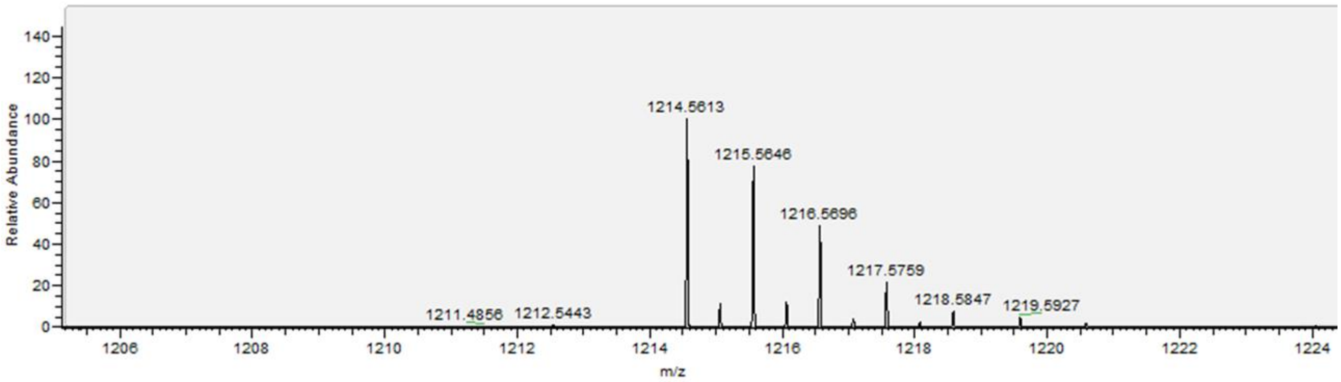

HRMS for conjugate 30, positive mode.

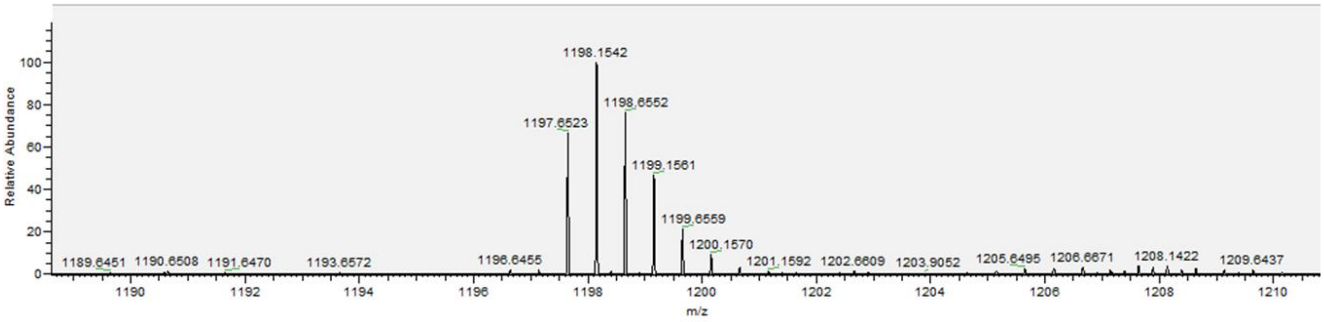

HRMS for conjugate 31, positive mode.

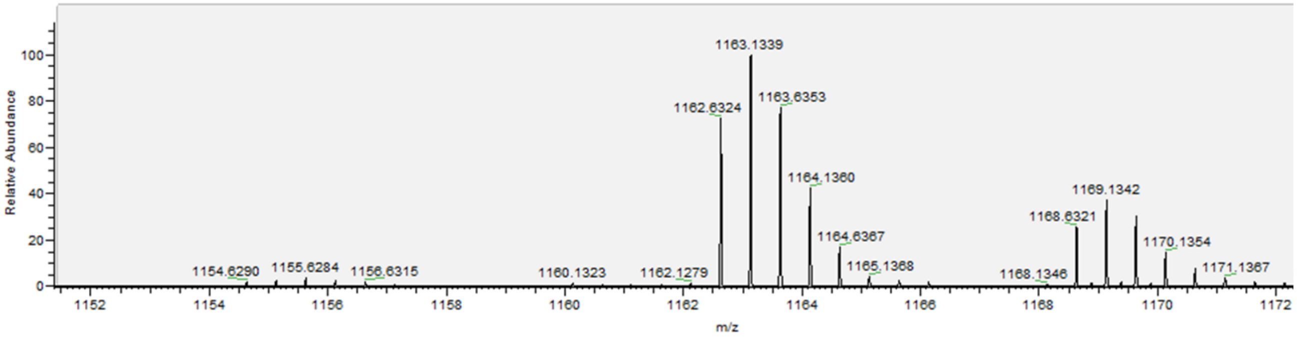

Supplement: Supplementary file 1 [file ijms-24-05134-s001.zip › ijms-2205133-supplementary.pdf]
